# Dynamic Expression of Membrane Type 1-Matrix Metalloproteinase (Mt1-mmp/Mmp14) in the Mouse Embryo

**DOI:** 10.3390/cells10092448

**Published:** 2021-09-17

**Authors:** Emma Muñoz-Sáez, Natalia Moracho, Ana I. R. Learte, Alicia G. Arroyo, Cristina Sánchez-Camacho

**Affiliations:** 1 Department of Health Science, School of Biomedical Sciences, Universidad Europea de Madrid, 28670 Villaviciosa de Odón, Spain; emma.munoz@universidadeuropea.es; 2 Department of Medicine, School of Biomedical Sciences, Universidad Europea de Madrid, 28670 Villaviciosa de Odón, Spain; natalia.moracho@universidadeuropea.es; 3 Department of Dentistry, School of Biomedical Sciences, Universidad Europea de Madrid, 28670 Villaviciosa de Odón, Spain; anaisabel.rodriguez@universidadeuropea.es; 4 Vascular Pathophysiology Department, Centro Nacional de Investigaciones Cardiovasculares (CNIC-CSIC), 28029 Madrid, Spain; agarroyo@cib.csic.es; 5 Molecular Biomedicine Department, Centro de Investigaciones Biológicas Margarita Salas (CIB-CSIC), 28040 Madrid, Spain

**Keywords:** Mt1-mmp, MMP14, metalloproteinases, embryo development, angiogenesis, expression pattern, cardiovascular development, brain, limb, dorsal aorta

## Abstract

MT1-MMP/MMP14 belongs to a subgroup of the matrix metalloproteinases family that presents a transmembrane domain, with a cytosolic tail and the catalytic site exposed to the extracellular space. Deficient mice for this enzyme result in early postnatal death and display severe defects in skeletal, muscle and lung development. By using a transgenic line expressing the LacZ reporter under the control of the endogenous Mt1-mmp promoter, we reported a dynamic spatiotemporal expression pattern for Mt1-mmp from early embryonic to perinatal stages during cardiovascular development and brain formation. Thus, Mt1-mmp shows expression in the endocardium of the heart and the truncus arteriosus by E8.5, and is also strongly detected during vascular system development as well as in endothelial cells. In the brain, LacZ reporter expression was detected in the olfactory bulb, the rostral cerebral cortex and the caudal mesencephalic tectum. LacZ-positive cells were observed in neural progenitors of the spinal cord, neural crest cells and the intersomitic region. In the limb, Mt1-mmp expression was restricted to blood vessels, cartilage primordium and muscles. Detection of the enzyme was confirmed by Western blot and immunohistochemical analysis. We suggest novel functions for this metalloproteinase in angiogenesis, endocardial formation and vascularization during organogenesis. Moreover, Mt1-mmp expression revealed that the enzyme may contribute to heart, muscle and brain throughout development.

## 1. Introduction

Extracellular matrix (ECM) is a dynamic structure present in all tissues that continuously undergoes remodeling mediated by specific enzymes. Amongst them, matrix metalloproteinases (MMPs) are well-known zinc-dependent endopeptidases with important roles in several physiological processes including angiogenesis, organogenesis and inflammation as well as in pathological conditions such as cancer progression, rheumatoid arthritis or Alzheimer’s disease [1,2,3]. MMPs are divided into soluble MMPs, secreted into the extracellular milieu by cells including fibroblasts, vascular smooth muscle cells (VSMC) and leukocytes among others, or expressed on the cell surface as a membrane-tethered form: membrane type-MMPs (MT-MMPs) [2,4]. The latter includes a subgroup of transmembrane MT-MMPs formed by MT1-, MT2-, MT3- and MT5-MMP that act altering the pericellular microenvironment of the cells expressing them [1,2,3]. MT1-MMP contains several domains conserved with other MMP family members: a signal peptide, a pro-domain and a catalytic domain. In addition, other domains are added to this basic core structure in MT1-MMP: a hinge region, a hemopexin-like domain, a linker, a hydrophobic membrane anchoring domain and a short cytoplasmic domain for intracellular interactions and signaling [3,5]. MT1-MMP is synthesized as zymogen and, thus, it is activated following a furin-dependent mechanism. The furin recognizes a sequence (RRKR^111^) located at the C-terminal end of the pro-domain and cleaves it for activation. This leads to the mature form of the enzyme, which later is expressed on the cell surface [1,2,3,5]. Either by shedding cell surface molecules or by producing biologically functional fragments from extracellular matrix, MT1-MMP is also known to act on other molecules as well as MMPs like pro-MMP-2 [6,7]. Regulation of its activity relies on its endogenous inhibition through, amongst others, TIMP-2 and the tumor suppressor RECK [3,5,8].

While the physiological functions of MT1-MMP are not yet fully understood, it has been intensively studied because its collagenolytic activity in the context of several pathological processes such as arthritis, cardiovascular disease and cancer progression [2,9]. MT1-MMP is the only MMP family member whose global deletion in mice results in early postnatal death. Due to the variety of the organs affected in MT1-MMP-targeted mice, identification of the essential functions of the proteinase during development has been a challenge. Previous studies have highlighted its pleiotropic functions, especially in the regulation of developmental events requiring ECM remodeling, and only a few studies have suggested its role in angiogenesis and cell polarity and migration in the embryo [10,11,12,13,14]. MT1-MMP localizes at the lamellipodia and invadopodia of migrating cells and, thus, it is a relevant promoter of angiogenesis through stimulation of endothelial cell invasion in the context of gastric, colon, head and neck cancer, what makes this proteinase an interesting therapeutic target [3,15,16]. However, despite that MT1-MMP plays critical roles in pathogenesis due to its pericellular proteolytic activity, little is known about its expression pattern during embryonic development and its functional relevance in this context apart from the skeletal, kidney and lung tissues [5,10,11,12,14,17,18,19,20].

A single pioneer report showed that MT1-MMP was mostly expressed in VSMCs as well as in cartilage during mouse embryonic development [17]. Later in the context of muscle development, MT1-MMP transcripts were observed in myoblasts, and it was identified as a major contributor to complete myoblast cell fusion and to promote skeletal muscle repair [21,22]. Evidence so far shows that MT1-MMP is also expressed distinctively in neural crest cells (NCCs) and its function is paramount during epithelial-mesenchymal transition and migration [23]. It is known that inhibition of metalloproteinase activity blocks NCCs migration, and also that MT1-MMP contributes to the formation of NCC derivative structures during embryogenesis [23]. Regarding the latter, it is known that loss of MT1-MMP function leads to severe skeletal abnormalities as well as impaired dental eruption and root formation [13,24,25,26]. Hence, it plays a relevant role in the formation of the head, teeth and their associated connective tissue by means of its collagenolytic activity. However, its detailed contribution to the formation of these structures during development remains unclear.

Another gap in the knowledge of the role of MT1-MMP during embryogenesis is its expression in the nervous system. It is known that its activity is instrumental to Central Nervous System (CNS) development, plasticity and repair, but no detailed analysis has been described yet. Expression of MT1-MMP has been reported in the olfactory bulb and the rostral migratory stream. On the other hand, its loss of function has been recently related to developmental abnormalities in the ventricular system, mainly with fewer and disorganized cilia in the ependymal cells [27]. Further, hypothalamic expression of MT1-MMP in the arcuate nucleus is required to establish the right neuronal connections in the center for appetite [28]. Besides, MT1-MMP has been reported during axonal degeneration and in the adult vertebrate eye [29,30,31,32]. As it is the case for other angiogenesis related genes, in the mouse retina its expression correlates with inner retinal vascularization [29]. In fact, defective MT1-MMP functioning has been linked to several neurodegenerative disorders due to its role in neovascularization [33]. However, despite our previous work about the expression of the subfamily member MT4-MMP during retinal development, MT1-MMP localization has not been reported in the embryonic eye [34].

MT1-MMP proteolytic activity controls branching events through degradation of ECM, but also acts as a hub for signal transduction cascades relevant for the control of cell motility [35,36,37,38,39,40]. Its function in regulation of branching morphogenesis may be relevant in several tissues like the submandibular and mammary glands [14,40]. In this context, MT1-MMP has been detected in the mammary branching ductal tree as well as in the surrounding mesenchyme [40,41]. However, rather than exert its proteolytic activity in remodeling during branching morphogenesis, it seems to play an unexpected role in its stroma, where it regulates adipose tissue differentiation [40]. Moreover, in the intestinal epithelium, MT1-MMP labeling has been observed in the postnatal development of rats and chickens, with no evidence so far of its expression pattern in the mouse embryo [42]. MT1-MMP regulation of branching morphogenesis is also well known to be of relevance in the case of renal and gonad development. Interestingly, its transcripts were found within developing kidney epithelial structures as well as in the mesenchymal cells around the bulbourethral gland [43]. Indeed, it is known that MT1-MMP activity is required by the primordial germ cells to reach the urogenital ridges, although the mechanism by which MT1-MMP exerts its function is still unclear [44,45]. MT1-MMP expression has also been found in both male and female gonads [43,44]. In the case of the female vertebrate gonads, MT1-MMP is constitutively expressed during the corpus luteum development as well as through life, playing important roles in the angiogenesis and tissue remodeling processes and also in the tissue degradation during luteal regression [46,47]. On the other hand, in the male gonad MT1-MMP is required for maturation and the migration of the gubernaculum in the developing testis [48,49].

In this study, we have analyzed MT1-MMP expression by a LacZ reporter transgene under its endogenous promoter at different stages of embryonic mouse development. Expression was also confirmed by immunohistochemistry and Western blot analysis. We report a dynamic expression pattern in the cardiovascular and nervous systems as well as in the limbs. This novel information may help understanding further the function of MT1-MMP in physiological and pathological conditions.

## 2. Materials and Methods

### 2.1. Animals

Mt1-mmp^LacZ/+^ mice in a C57BL/6 genetic background expressing LacZ reporter under the control of the endogenous MT1-MMP promoter were generated as described and provided by Prof. Motoharu Seiki [50]. Wild type and Mt1-mmp^LacZ/+^ littermate embryos from pregnant mice were collected between embryonic stages E8.5–17.5 (E0.5 correspond to the day of the vaginal plug). Mice were housed in the Centro Nacional de Investigaciones Cardiovasculares (CNIC) Animal Facility under pathogen-free conditions and in strict accordance with the institutional guidelines. Animal studies were approved by the CNIC Animal Experimentation Ethics Committee and by the Community of Madrid (Ref. PROEX 34/13). All animal procedures conformed to EU Directive 2010/63EU and Recommendation 2007/526/EC regarding the protection of animals used for experimental and other scientific purposes, enforced in Spanish law under Real Decreto 1201/2005.

### 2.2. β-Galactosidase Staining

MT1-MMP expression was analyzed by using X-gal histochemistry in Mt1-mmp^LacZ/+^ embryos as previously described [51]. Small mouse embryos, from E8.5 to E12.5, were fixed by immersion in 0.125% glutaraldehyde in phosphate buffer saline 1X pH 7.4 (PBS) for 3 h at room temperature and processed for in toto LacZ staining. Briefly, the whole embryo was incubated in X-gal buffer (5 mM potassium ferrocyanide, 5 mM potassium ferricyanide, 2 mM MgCl_2_, 0.01% deoxycholate acid, 0.02% NP-40 and 0.1% X-gal) at 37 °C overnight. Following PBS washing, embryos were fixed in 4% paraformaldehyde (PFA), and then paraffin-embedded and sectioned in the frontal plane at 7 µm thickness. Sections were counterstained with Fast Red for nuclear labeling that helps to define different embryonic structures. For those embryos older than E12.5 and postnatal stages, animals were transcardially perfused with 0.125% glutaraldehyde in PBS and post-fixed for 2–12 h at 4 °C. Then, they were cryoprotected in 30% sucrose solution in PBS, embedded in OCT (Thermo Fisher Scientific, Waltham, MA, USA) and sectioned in the frontal plane with a CM 1750 cryostat (Leica, Heidelberg, Germany). Coronal sections of 20 µm thickness were collected and then processed for LacZ staining as described above. A minimum of three mouse embryos per stage were used in the analysis of Mt1-mmp expression.

### 2.3. Protein Extraction and Western Blot Analysis

Protein extracts from embryonic mouse tissues (olfactory bulb, mesencephalon, cerebral cortex, spinal cord, forelimb and heart) were obtained from E14.5 wildtype (WT), heterozygous (HT), and knock-out (KO) mice. All the tissues were homogenized in 60 µL of cold lysis buffer (2% NP40, 20 mM HEPES pH 7.4, 100 mM NaCl, 100 mM NaF, 1 mM NaVO_4_, 5 mM EDTA) including protease inhibitors (P8340, Sigma, St. Louis, MO, USA). Protein concentration was measured by the BCA protein assay (Pierce TM BCA Protein Assay Kit, Thermo Fisher Scientific). Western blotting was performed with 50 µg of each protein extract resolved by 10% SDS-PAGE and transferred onto nitrocellulose membranes (0.45 µm, BioRad, Hercules, CA, USA). Membranes were blocked with 5% non-fat milk in PBS. Polyclonal antibodies against MT1-MMP MT-loop (EP1264Y, ab51074, abcam, Cambridge, UK) and mouse monoclonal anti-β-actin (A5441, Sigma-Aldrich) were used at 1:2500 and 1:1000 dilution in 1% powder non-fat milk in tris buffer saline (Tween-20 0.1%, NaCl 150 mM, Tris 20 mM pH 7.5) overnight, respectively. Then, membranes were developed using a 1:2500 dilution of both goat anti-rabbit HRP (111-035-003, Jackson Immunoresearch, PA, USA) and goat anti-mouse HRP (115-035-003, Jackson Immunoresearch) antibodies. After the membranes were rinsed, a chemiluminescent detection system (Pierce™ ECL Western Blotting Substrate, 32106, Thermo Fisher Scientific) was used for protein detection by ChemiDoc XRS+ (BioRad) using Image Lab 5.2.1 software.

### 2.4. Immunohistochemistry

For immunohistochemical procedures, E11.5 embryos were fixed in 4% PFA in PBS 1× pH 7.4 by immersion or perfusion depending on the developmental stage, cryoprotected in a 30% sucrose solution, and then embedded in OCT. Sections at 15 µm thickness were obtained in the transverse plane in the CM1950 cryostat (Leica). Afterwards, immunohistochemical staining was performed following standard protocols. Sections were permeabilized with 0.3% Triton X-100 in PBS for 15 min at room temperature and then incubated with a blocking solution containing 1% bovine serum albumin (BSA) and 0.1% Triton X-100 in PBS for 1 h at room temperature with gentle rolling. After washing the sections three times for 5 min each in PBS at room temperature, they were incubated with the primary antibodies in blocking solution for 24 h at 4 °C. Primary antibodies used included: hamster polyclonal anti-CD31 (1:1000; MAB1398Z, Millipore, Billerica, MA, USA), rabbit anti-*β*-galactosidase (1:1000; ab4761, abcam, Cambridge, UK), mouse anti-Nkx6.1 (1:100; gift from Dr. AV Morales, CSIC), rabbit anti-ERG antibody EPR3864 Alexa Fluor^®^ 647 (1:500; ab196149, abcam), and the mouse anti-MT1-MMP monoclonal antibody, LEM 2/15 (1:1) [52]. Sections were incubated with the primary antibody diluted in PBS containing 0.1% Triton X-100 and 1% bovine serum albumin (BSA), for 24 h at 4 °C. Subsequently, the sections were rinsed in PBS and incubated for 2 h at room temperature with 488 or 594-Alexa Fluor®-conjugated fluorescent antibodies (1:500; Thermo Fisher Scientific). Sections were counterstained with Hoechst (1:1000; Molecular Probes, Eugene, OR, USA) for 5 min at room temperature to visualize nuclei and then mounted in Fluoromount-G (0100-01, SouthernBiotech, Birmingham, AL, USA). Images were acquired on an inverted confocal microscope (LSM700, Carl Zeiss, Jena, Germany) and processed using Zen2011 software (Carl Zeiss).

### 2.5. Statistical Analysis

Statistical differences in protein expression among different tissues of E14.5 WT embryos (*n* = 3) were assessed by a non-parametric Kruskal–Wallis test followed by its corresponding Post Hoc Analysis that was performed using GraphPad Prism 6.0 (GraphPad Software, Inc., La Jolla, CA, USA) and IBM SPSS Statistics v23. Data are represented as mean ± s.e.m and differences were considered significant at *p* < 0,05 (* *p* < 0.05; ** *p* < 0.01 and *** *p* < 0.001). For Western blot analysis, the intensity of the specific bands were analyzed using Image J 1.8.0_66 (64 bit) software (NIH).

## 3. Results and Discussion

In this study, we analyzed MT1-MMP expression by a LacZ reporter transgene under its endogenous promoter (from now on, MT1-MMP in the text) at different stages of embryonic mouse development (from E8.5 to E17.5) [50]. This reporter mouse model allowed us to confirm MT1-MMP expression at certain embryonic tissues and, more importantly, to identify a dynamic pattern of expression for MT1-MMP particularly in the developing cardiovascular and nervous systems and in the limbs. To date, only few reports have claimed that MT1-MMP may play a role during embryonic angiogenesis according to the vascular defects observed during lung morphogenesis or vascular invasion of cartilage [13,14,53]. Based on our results about MT1-MMP expression in endothelial cells of distinct embryonic tissues, we suggest that the metalloproteinase may be relevant for vascularization during organogenesis in the embryo as well as during heart development. In the same line, no information on the expression of MT1-MMP during brain development has been reported so far. However, we detect that MT1-MMP localizes during the development of distinct structures of the nervous system such as the olfactory bulb, the cerebral cortex or the superior colliculus, for example. This novel information may help understand the functional pleiotropism of MT1-MMP in pathophysiology.

### 3.1. Dynamic Expression of MT1-MMP during Cardiovascular Development

**Figure 1:**
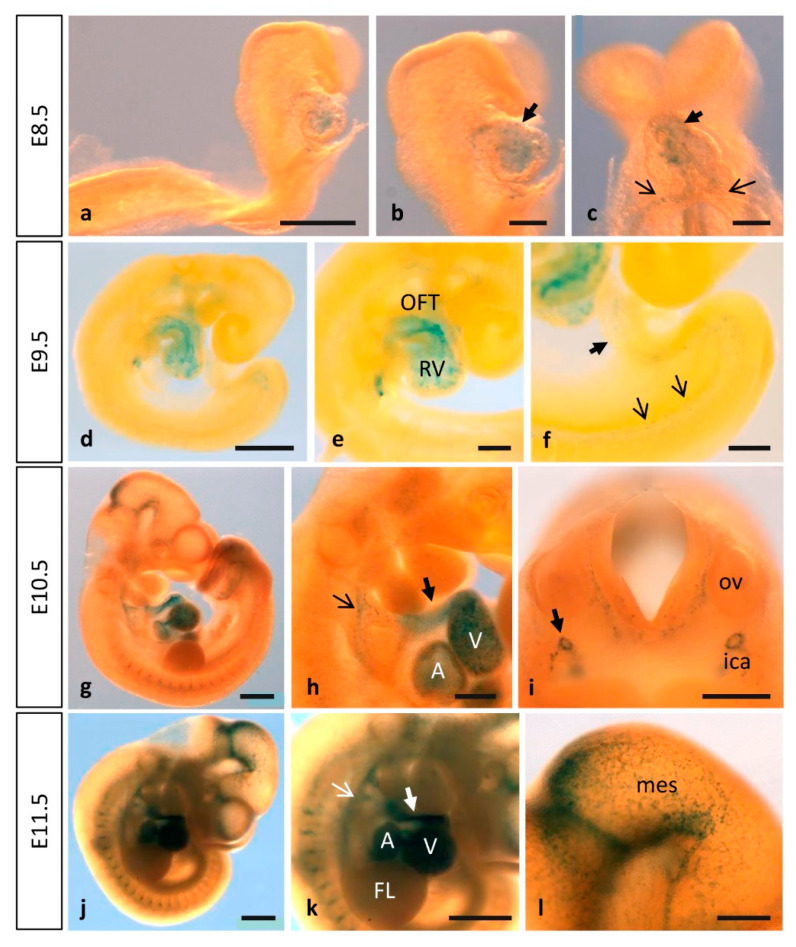
MT1-MMP is expressed in the early mouse embryo. Whole-mount β-gal staining in Mt1-mmp^LacZ/+^ embryos from E8.5 to E11.5. Staining is observed in the endocardium of the developing heart tube (**a**–**c**) as early as E8.5 embryonic stage. At later stages of development, this expression persists and the strongest labeling is observed in the atrium, the ventricle, the cardiac outflow tract (black and white thick arrows in (**h**,**k**)), the aortic arch arteries (black and white thin arrows in (**h**,**k**)) and the dorsal aorta at E9.5 (**d**–**f**), E10.5 (**g**–**i**) and E11.5 (**j**–**l**). Expression in the central nervous system was first detected by E10.5 (**g**) and E11.5 (**j**) in the caudal midbrain and the rostral rhombencephalon (**l**). Furthermore, β-gal positive cells were detected in the perineural vascular plexus and the first blood vessels entering the brain (**i**,**l**). Somites also show β-gal positive cells at these embryonic stages (**g**,**j**,**k**). Abbreviations: A, atrium; FL, forelimb; ica, internal carotid artery; mes, mesencephalon; OFT, outflow tract; ov, otic vesicle; RV, right ventricle; V, ventricle. Scale bars: 1 mm (**g**,**j**,**k**), 500 µm (**a**,**d**,**h**,**i**,**l**), 200 µm (**b**,**c**,**e**,**f**).

**Figure 2:**
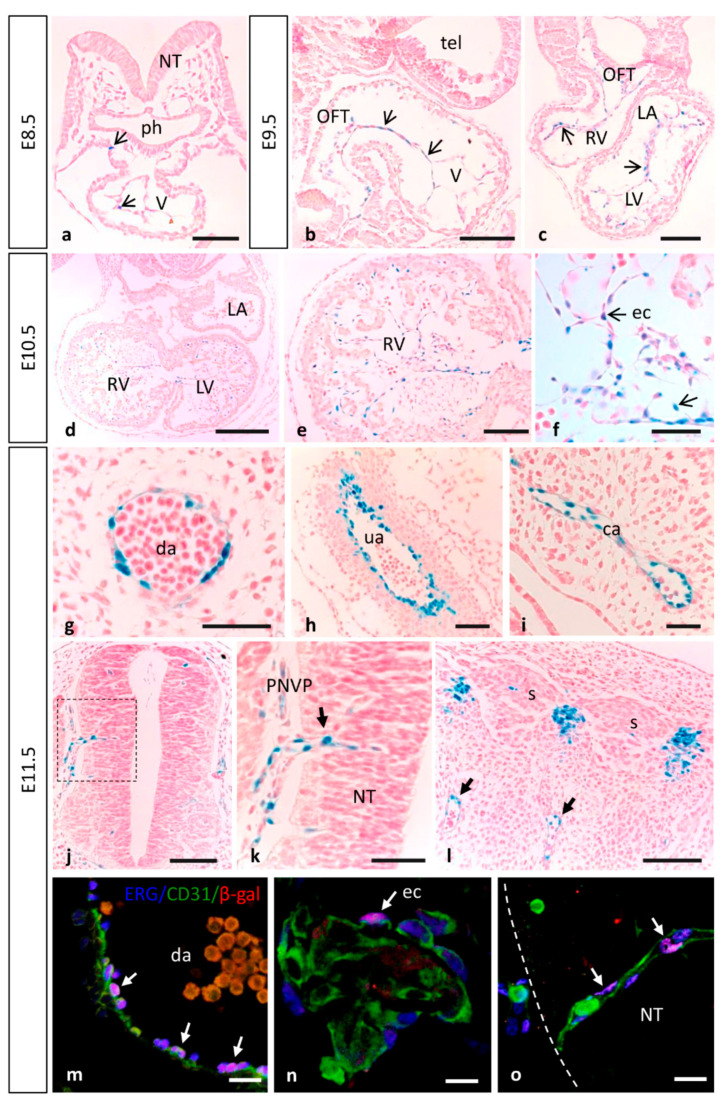
MT1-MMP is expressed at early embryonic stages in the developing cardiovascular system. (**a**–**f**) β-gal staining of paraffin sections from E8.5 to E10.5 Mt1-mmp^LacZ/+^ embryos revealed expression of MT1-MMP in the endocardial tissue lining the primitive heart tube at early embryonic stages (arrows in (**a**)), and the endocardium of the atrium, the ventricle and the cardiac outflow tract at E9.5 (arrows in (**b**), sagittal section; arrows in (**c**), coronal section) and at E10.5 (**d**–**f**). (**g**–**l**) Mt1-mmp^LacZ/+^ expression was detected in large blood vessels such as the dorsal aorta (**g**), the umbilical artery (**h**) and the carotid artery (**i**) of E11.5 embryos. At this embryonic stage, β-gal positive cells were detected in the perineural vascular plexus that surrounds the neural tube (**j**,**k**) and in somites (**l**), in a pattern compatible with intersomitic arteries (arrows in (**l**)). Note the β-gal labeling in endothelial cells of a blood vessel sprouting into the neural tube (arrow in (**k**)). (**m**–**o**) Confocal images of immunohistochemistry for the endothelial transcription factor ERG (blue), the membrane marker for endothelial cells CD31 (green) and the reporter β-gal (red), demonstrate MT1-MMP expression in endothelial cells of the dorsal aorta (**m**) and the endocardial endothelium of the primitive heart tube (**n**) of E11.5 embryos. MT1-MMP expression was also demonstrated in the endothelial cells from the first blood vessels entering the brain parenchyma (**o**). See images from split channels in Figure S1. Abbreviations: ca, carotid artery; da, dorsal aorta; ec, endothelial cell; OFT, outflow tract; LA, left atrium; LV, left ventricle; NT, neural tube; ph, pharyngeal region; PNVP, perineural vascular plexus; RV, right ventricle; s, somite; tel, telencephalon; V, ventricle; ua, umbilical artery. Scale bars: 250 µm (**d**), 100 µm (**a**–**c**,**e**,**j**,**l**), 50 µm (**f**,**g**,**k**,**m**–**o**), 20 µm (**h**,**i**).

**Figure 3:**
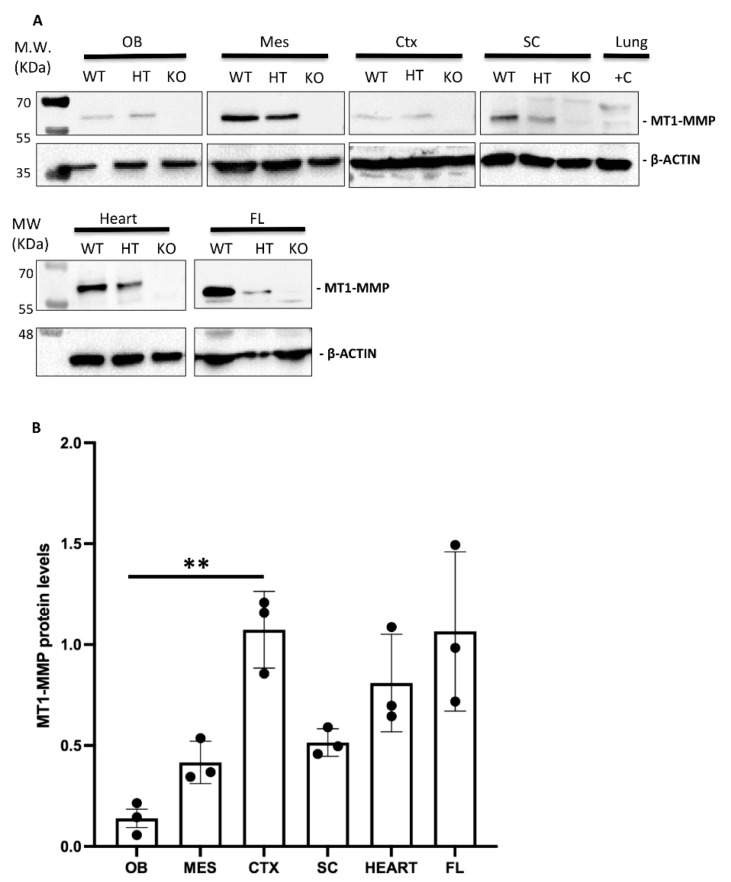
(**A**) Western blot analysis confirms MT1-MMP protein expression in distinct embryonic tissues. (**A**) Protein detection by Western blot of E14.5 embryonic tissues revealed MT1-MMP expression in the olfactory bulb (OB), mesencephalon (MES), cerebral cortex (CTX), spinal cord (SC), heart and forelimbs (FL). Specific bands correspond to the mature form of MT1-MMP (57 kDa) and β-actin (42 kDa). Adult lungs were used as a positive control (+C lane) due to the high expression of the protein in this tissue. As expected, MT1-MMP protein levels were significantly decreased in the heterozygous (HT) compared to the wild type (WT), and absent in the knock-out (KO) embryonic tissues. (**B**) Quantification of Western blot in WT embryonic tissues demonstrates higher levels of the active MT1-MMP in the cerebral cortex, heart and the forelimbs compared to the olfactory bulb, mesencephalon and spinal cord (** indicates significant differences at *p* < 0.01) (*n* = 3 per tissue). MT1-MMP protein levels were expressed as arbitrary units/50 µg protein.

By means of β-galactosidase staining in whole mouse embryos and sections, MT1-MMP was detected in the cardiovascular system at distinct developmental stages (Figure 1 and Figure 2). The earliest expression of MT1-MMP associated to heart development was detected by E8.5. Thus, β-gal positive cells were restricted to the truncus arteriosus and the left and right ventricles of the developing heart tube by 6–8 somite staged embryo (Figure 1a–c). Additionally, β-gal staining was observed in the primitive left and right atrium at this embryonic stage. At later stages of development, this expression persists and a stronger LacZ labeling was observed in the developing heart of E9.5, E10.5 and E11.5 embryos, particularly in the cardiac outflow tract (OFT), but also in the atrium and the ventricle (Figure 1d,e,g,h,j,k). Analysis of paraffin sections from Mt1-mmp^LacZ/+^ embryos stained with β-gal revealed that MT1-MMP expression is present in the endocardial tissue lining the primitive heart tube at early embryonic stages (E8.5, Figure 2a). Expression in the endocardium of the atrium and ventricle as well as in the OFT is also present at E9.5 (Figure 2b,c) and E10.5 embryonic stages (Figure 2d–f) and persists to perinatal stages (E17.5) (data not shown). In contrast to previous in situ hybridization data (ISH), we did not detect LacZ staining in the myocardium at E12.5, and we did observe LacZ staining in endocardial endothelial cells [16]. Moreover, our data showed expression in the cardiac OFT and large arteries from earlier embryonic stages than previously reported. We confirmed the expression of MT1-MMP in endothelial cells of the endocardium by immunohistochemistry for β-gal and the endothelial markers CD31 and ERG (Figure 2n and Figure S1e–h). To confirm these results based on the LacZ reporter expression of Mt1-mmp gene, we analyzed by Western blot the protein expression in the embryonic heart. As shown in Figure 3, high levels of MT1-MMP were expressed in this tissue in E14.5 WT embryos with lower levels in the HT tissue, and the absence of the protein in the KO heart. These data are supported by the detection of Mt1-mmp mRNA levels in the embryonic heart at perinatal stages [20]. Our results suggest that MT1-MMP may play a relevant role in the morphoregulatory processes during heart organogenesis related to endocardial signals such as ventricular trabeculation, valve formation and coronary artery development [54,55,56]. In this regard, EMT occurs in the endocardial cushions during early heart development for the proper formation of the atrial and ventricular septae and valves. This process involves metalloproteinase activity for degradation of type IV collagen in the endocardial basement membrane by MMP-2 and MT1-MMP and MMP-9 to facilitate migration of mesenchymal cells [57,58,59]. In these contexts, MT1-MMP may modulate EMT signals via its proteolytic activities on ECM components or transmembrane proteins and correlating with the expression of its main inhibitor TIMP2 during cushion formation in the embryonic heart [17,60]. We have recently reported that a GPI-membrane-anchored MMP, MT4-MMP, is also expressed in the embryonic mouse heart. In this case, MT4-MMP expression in the endocardial endothelium of the atrium and ventricle was detected only in the early embryo, whereas labeling was restricted to the epicardium at later stages of development [34]. As these MT-MMPs are among the least homologous in protein sequence, our data supports functional specificities for MT1-MMP and MT4-MMP during cardiac development.

Previous analysis by ISH has shown MT1-MMP expression in the media layer constituted by VSMCs in several large arteries of the embryo but negative in endothelial cells, despite of defective vascular invasion and expansion reported in cartilage and lung, respectively [13,14,17]. Moreover, MT1-MMP function was demonstrated essential for arterial structure and mural cell function through the regulation of PDGF-B-PDGFRβ signaling in VSMC differentiation [53]. Even though we detected MT1-MMP highly expressed in the endothelium, the mechanism through which it contributes to the vasculature remains unknown. Our approach with the LacZ-reporter allowed us to detect Mt1-mmp promoter activity i.e., in the dorsal aorta, the internal carotid artery (rostral extension of the dorsal aorta), the aortic arch arteries, and the OFT from E8.5 to E12.5 (Figure 1 and Figure 2) as well as in the somites, in a pattern compatible with intersomitic arteries (Figure 2). Mt1-mmp^LacZ/+^ expression was also detected in other large blood vessels such as the umbilical artery and vein and the primary head vein from E11.5 to E17.5 embryos (Figure 2g–i). Previous studies have pointed out the presence of MT1-MMP in the cardiac OFT, especially at the root of the aorta correlating with the prospective semilunar valves [17]. Further, β-gal positive cells localized in the perineural vascular plexus (PNVP) that surrounds the neural tube as well as in blood vessels sprouting into the parenchyma of the neural tube and the mesencephalon at these embryonic stages (E10.5, E11.5) (Figure 2j,k). This labeling in the brain blood vessels extends to other brain regions as development proceeds. Notably, by means of immunohistochemistry for β-gal and the endothelial transcription factor ERG and the membrane marker CD31, we confirmed the expression of MT1-MMP in endothelial cells of the dorsal aorta and brain blood vessels (Figure 2m,o and Figure S1a–d,i–l). This expression of the proteinase in endothelial cells is in line with the pleiotropic actions demonstrated for MT1-MMP in angiogenesis [17,61,62]. Additionally, β-gal staining was observed in the meninges, the posterior and middle cerebral arteries and the anterior cerebral vein at E12.5. This is coincident with the expression pattern of MT4-MMP in large blood vessels shown in our previous work, although MT1-MMP seems to exhibit higher levels of expression in the endothelium compared to MT4-MMP which is highly expressed in VSMCs [34,63].

Cells localize their MT1-MMP at lamellipodia of migrating cells to promote motility and invasion both in physiological and pathological conditions [3,15]. Thus, MT1-MMP is a key regulator of cell migration through its extracellular proteinase activity that degrades pericellular ECM and processes cell adhesion molecules, but also via its intracellular non-proteolytic pathways [15]. Consequently, MT1-MMP is a relevant promoter of angiogenesis through pro-MMP-2 activation and stimulation of endothelial cell migration by directly degrading the pericellular matrix [52,64,65,66]. Several studies have shown that MT1-MMP is highly enriched in endothelial tip cells and required for sprouting angiogenesis [50,67,68]. During blood vessel growth, a link between matrix proteolysis and cell motility must be established for successful invasion of the endothelial cells. Also during angiogenic sprouting, endothelial cells require the membrane associated MT1-MMP for luminal expansion and formation of vascular guidance tunnels [69]. Regarding the molecular mechanism that may regulate the role of MT1-MMP in both processes, it is known that endothelial cells devoid of the angiogenic sprouting mediator Slug show reduced MT1-MMP levels, in a phenotype that is rescued by the re-expression of the proteinase [70]. Therefore, we can hypothesize that one possible mechanism could be the selective processing of different substrates as seen in endothelial cell sprouting during inflammatory angiogenesis, where MT1-MMP action relies on the processing of several substrates in a combinatorial proteolytic program [71]. This suggests a fine regulation of the angiogenic response through adhesion, motility and chemotaxis, which ultimately would lead to vascular development. Vascular expansion can also occur by intussusceptive angiogenesis, a process associated to organ expansion as in the lung but also to pathologies as inflammatory bowel disease in which microvasculature expands through capillary splitting via rearrangement of endothelial cells [72]. It has been demonstrated that MT1-MMP is required for nitric oxide (NO) production via cleavage of thrombospondin-1 (TSP1) by MT1-MMP, which produces a C-terminal TSP1 fragment that binds to CD47/αvβ3 integrin. NO production in this context induces vasodilation in arterioles, which would initiate intussusceptive remodeling [73]. This functional link between NO and MT1-MMP has been shown before during endothelial cell migration and cord formation. At the motility-associated EC membrane cell protrusions, where MT1-MMP function requires eNOS, the enzyme responsible for NO production, this mechanism may regulate the activation of EC to transition into a migratory state [74].

Previous studies also highlighted the presence of MT1-MMP in the junction between uterine decidua and placental spongiotrophoblasts [17,20]. We detect MT1-MMP expression in the umbilical vessels. In this regard, the data shown suggests a possible role of MT1-MMP at least in directing the extension of the developing umbilical vessels (namely two arteries and a vein) through the Wharton’s jelly, a gelatinous proteoglycan-rich substance derived from the mesoderm enriched in collagen type I and type III, both MT1-MMP substrates [75]. It is known that MT1-MMP acts as a key pericellular collagenase that controls the ability of VSMCs to degrade and infiltrate 3D barriers of interstitial collagen, including the arterial wall [76]. Moreover, MT1-MMP has a greater catalytic activity on type III collagen found in visceral and cardiovascular tissues [77,78]. ECM dynamics perform a major influence on umbilical VSMCs fate, although the mechanisms by which the umbilical cord vascular growth happens are poorly understood. We hypothesize that MT1-MMP may play a critical role in the process similarly to the secreted metalloproteinase ADAMTS9, that regulates VSMC proliferation and differentiation non autonomously through its interaction with PDGFRβ [79].

### 3.2. MT1-MMP Is Highly Expressed during Nervous System Development

**Figure 4:**
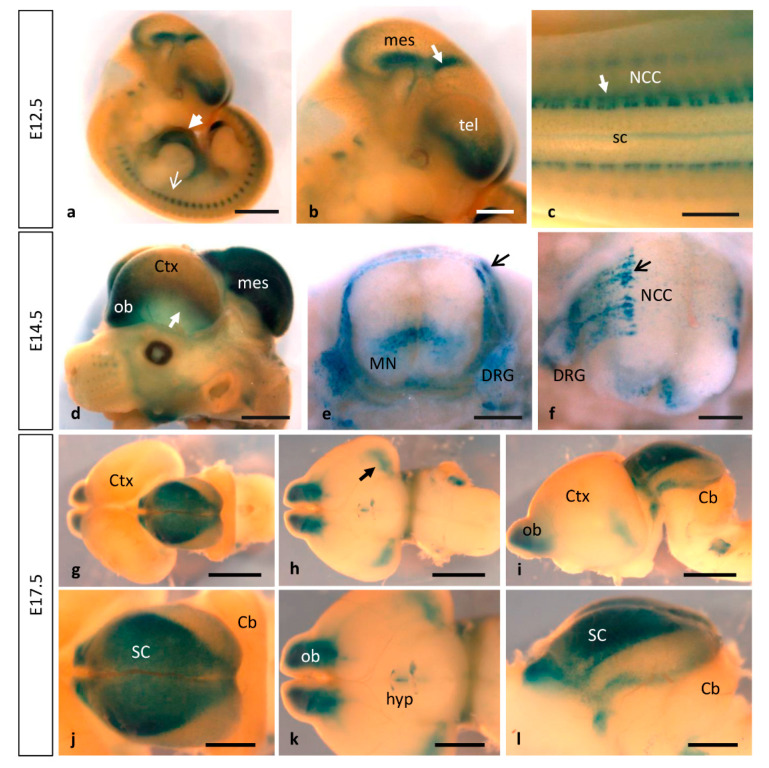
Dynamic expression of MT1-MMP at later stages of brain and embryo development. As development proceeds, stronger pattern of MT1-MMP expression persists in the cardiovascular system and the brain. (**a**–**c**) E12.5 Mt1-mmp^LacZ/+^ embryos show reporter expression in the developing heart (white arrowhead in a), somites (white arrow in (**a**)) and at distinct regions of the brain: the rostral telencephalon (the prospective olfactory bulb), zona limitans intrathalamica (white arrow in (**b**)), the floor plate and the caudal mesencephalon and neural crest cells (white arrow in (**c**)). (**d**–**f**) At E14.5, MT1-MMP expression increases in the olfactory bulb and the caudal cerebral cortex (arrow in a), the dorsal part of the mesencephalon and in neural progenitors and motoneurons of the spinal cord (**e**,**f**). Further, β-gal positive cells were located in streams of migrating neural crest cells (arrow in (**e**,**f**)), the dorsal nerve root and the dorsal root ganglia. (**g**–**l**) Later in development, LacZ staining persists in the brain at E17.5: in the olfactory bulb, the amygdala (arrow in (**h**,**k**)), the hypothalamic region and the superior colliculus ((**g**,**j**), dorsal view; (**h**,**k**) ventral view; (**i**,**l**), lateral view of the brain). Abbreviations: Cb, cerebellum; Ctx, cerebral cortex; DRG, dorsal root ganglia; hyp, hypothalamic region; mes, mesencephalon; MN, motoneurons; NCC, neural crest cell; ob, olfactory bulb; sc, spinal cord; SC, superior colliculus; tel, telencephalon. Scale bars: 2 mm (**a**,**d**,**g**–**i**), 1 mm (**b**,**c**,**j**–**l**), 500 µm (**e**,**f**).

**Figure 5:**
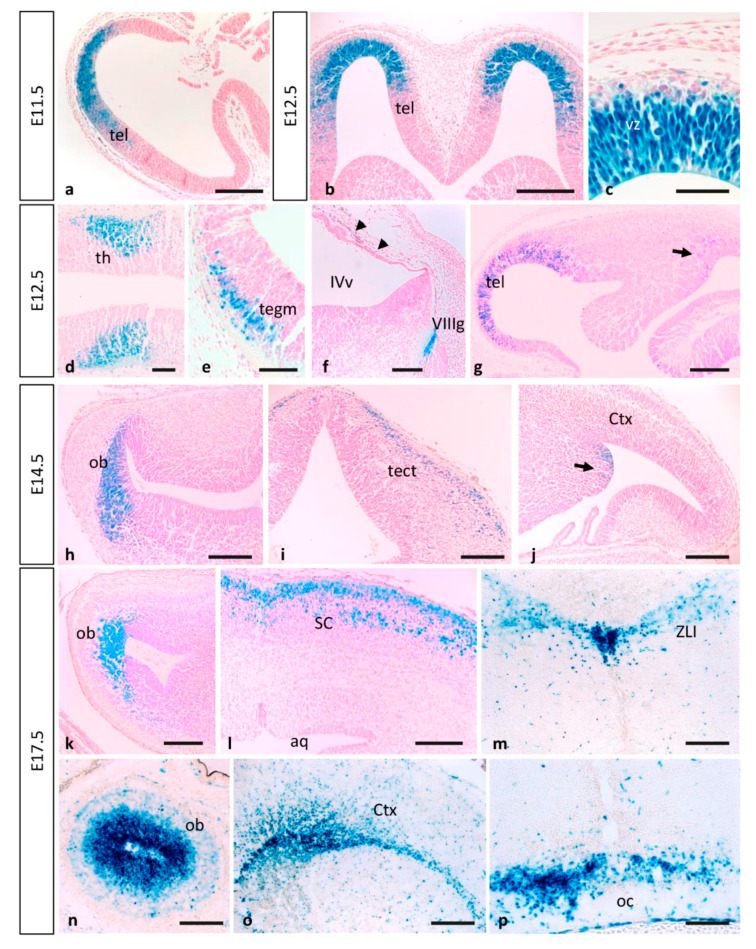
Expression of MT1-MMP in the developing mouse brain. β-gal staining of horizontal (**a**–**c**,**g**,**h**,**k**) and coronal sections (**n**) from E11.5 to E17.5 Mt1-mmp^LacZ/+^ embryos revealed high expression of MT1-MMP in the ventricular zone of the rostral telencephalon at early embryonic stages (**a**–**c**,**g**) that will give rise to the olfactory bulb later in development (**h**,**k**,**n**). In addition, β-gal positive cells were detected in the thalamus (**d**), the mesencephalon (**e**), the VIII nerve ganglia (**f**) and the meninges (arrowheads in **f**). At this embryonic stage, β-gal stained cells appear in the caudal portion of the telencephalon (arrow in **g**) where the amygdala will develop later (arrow in **j**) at E14.5. LacZ-positive cells were also located in the mesencephalic tectum of E14.5 embryos (**i**) and restricted to the superficial layers of the superior colliculus by E17.5 (**l**). By this perinatal stage, strong LacZ staining localized in the periventricular zone of the III ventricle and the zona limitans intrathalamica (**m**) and the optic chiasm region (**p**). Streams of β-gal positive cells entered the cerebral cortex by E17.5 (**o**). Abbreviations: aq, cerebral aqueduct; Ctx, cerebral cortex; ob, olfactory bulb; oc, optic chiasm; tect, mesencephalic tectum; tegm, mesencephalic tegmentum; SC, superior colliculus; tel, telencephalon; th, thalamus; vz, ventricular zone; IVv, IV ventricle; VIIIg, VIII nerve ganglia; ZLI, zona limitans intrathalamica. Scale bars: 250 µm (**a**,**b**), 200 µm (**f**–**p**), 100 µm (**d**,**e**), 50 µm (**c**).

No detailed expression analysis for MT1-MMP in the developing nervous system has been described previously. Our present study shows that MT1-MMP distribution follows a restricted temporal and spatial pattern of expression during mouse brain development (Figure 4 and Figure 5). We detected the first levels of expression of MT1-MMP in the floor plate and the ventral neural tube at E9.5 in the mouse embryo (data not shown). By E10.5, LacZ staining was found in the caudal mesencephalon (Figure 1g). As shown in whole-mount β-gal-stained embryos, reporter expression extends in the brain as development proceeds and it is detected at distinct brain regions from E11.5 to E12.5: the rostral (the prospective olfactory bulb) and the caudal and ventral telencephalon, the zona limitans intrathalamica and the floor plate (Figure 1j–l and Figure 4a,b). Detailed analysis of β-gal staining of Mt1-mmp^LacZ/+^ sections revealed a strong expression of MT1-MMP in the neural progenitors within the ventricular zone of the rostral telencephalon at early embryonic stages (Figure 5a–c,g) that will give rise to the olfactory bulb later in the embryo (Figure 5h,k,n). In addition, LacZ reporter expression was observed in the caudal portion of the telencephalon, the thalamus, the early postmitotic neurons within the mantle zone of the mesencephalon and the VIII nerve ganglia (Figure 5d–f and Figure S2).

**Figure 6:**
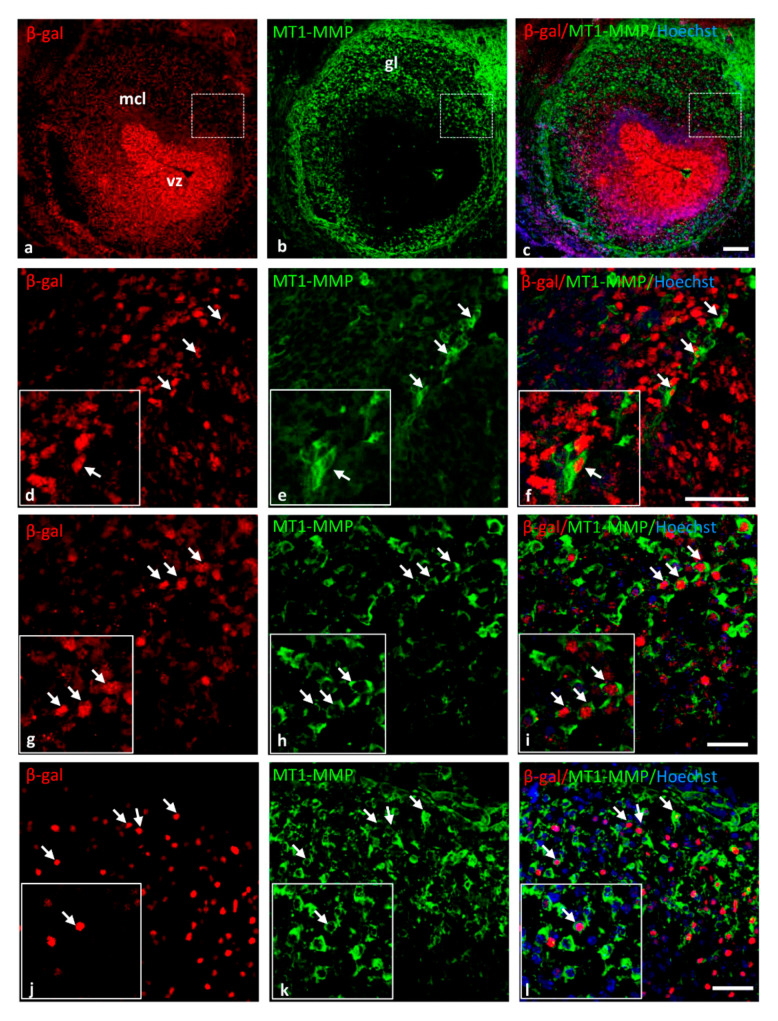
MT1-MMP is expressed in the olfactory bulb, the cerebral cortex and the superior colliculus of E14.5 HT embryos. Double-labeling immunohistochemistry for β-gal (in red) and MT1-MMP (in green) confirmed the expression of the enzyme and the reporter β-gal (white arrows) in the olfactory bulb (**a**–**f**), the cerebral cortex (**g**–**i**) and the superior colliculus (**j**–**l**). Abbreviations: gl: glomerular layer; mcl: mitral cell layer; vz: ventricular zone. Scale bars: 100 µm (**a**–**c**) and 50 µm (**d**–**l**).

As development progressed, MT1-MMP expression increases in the olfactory bulb, the caudal and ventral telencephalon where the amygdala will develop later, and superficial layer of the mesencephalic tectum by E14.5 (Figure 4d–f, Figure 5h–j and Figure 6). Whole mount LacZ-stained brains revealed that a strong staining persists in the mouse embryo by E17.5, particularly in the olfactory bulb, the amygdala, superficial layers of the superior colliculus and the optic chiasm in the hypothalamic region (Figure 4g–l and Figure 5k–p). MT1-MMP expression also localized in streams of cells within the cerebral cortex, the periventricular zone of the III ventricle and the zona limitans intrathalamica (Figure 5m,o). Although there is no detailed analysis of MT1-MMP expression, mRNA levels have been detected in the embryonic brain supporting our data [20]. Recently, LacZ staining was reported in the olfactory bulb, neural progenitors of the ventricular zone, rostral migratory stream and midbrain of postnatal brains [27]. Indeed, deficient mice for MT1-MMP display several defects in brain development including corpus callosum agenesis, enlarged ventricles and astrocytosis. Supporting a role for MT1-MMP in early postnatal brain development, the proteinase is necessary for ependymal cell maturation and ciliogenesis via suppression of Notch signaling, and the lack of its activity leads to hydrocephalus [27]. The relevance of the proteinase in the adult brain has been brought out with the correlation of its expression in the hypothalamus and the wasting phenotype observed in the mutant mice. Thus, loss of MT1-MMP function alters distribution of two neuropeptides, neuropeptide Y (NPY) and agouti-related protein (AgRP), in the arcuate nucleus which is implicated in the regulation of food intake and body weight control [28].

**Figure 7:**
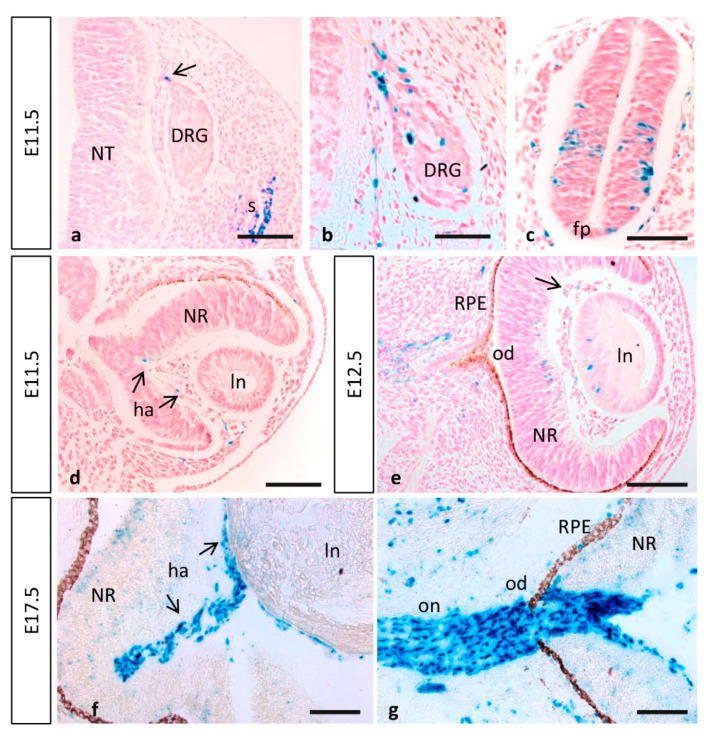
MT1-MMP expression in the embryonic spinal cord and eye. (**a**,**b**) MT1-MMP expression was detected in the spinal cord particularly in neural crest cells, somites, the dorsal nerve root and the dorsal root ganglia at E11.5. Reporter expression was also observed in neural precursors of the spinal cord and the floor plate (**c**). (**d**,**e**) In the developing eye, β-gal positive cells localize in the hyaloid artery at E11.5 (arrows in (**b**)) and E12.5 (arrow in (**e**)). Further, scattered cells were detected in the lens and the neural retina by E12.5 (**e**). Later in development, MT1-MMP expression increases in the hyaloid artery and is present in cells surrounding the optic nerve and at the level of the optic disc and optic nerve head, possibly corresponding to glial cells (**f**,**g**). Abbreviations: DRG, dorsal root ganglia; fp, floor plate; ha, hyaloid artery; od, optic disc; on, optic nerve; ln, lens; NR, neural retina; NT, neural tube; RPE, retinal pigmented epithelium. Scale bars: 100 µm (**a**–**g**).

Interestingly, strong labeling was observed in a segmented manner along the entire neural tube corresponding to migrating neural crest cells (NCCs). These β-gal positive cells were distributed in streams of migrating cells, along the dorsal nerve root and in the dorsal root ganglia in the spinal cord from E10.5 to E14.5 (Figure 1g,j,k and Figure 4c,e,f and Figure 7a,b). At more caudal levels, reporter expression was observed in neural precursors and early postmitotic neurons of the ventral spinal cord by E10.5–E12.5 and the pool of motoneurons at E14.5 (Figure 4e,f and Figure 7c). Immunohistochemistry for the transcription factor Nkx6.1, expressed in ventral progenitors that give rise to motoneurons and ventral interneurons, in combination with β-gal antibodies showed that, although MT1-MMP expression localized in this region, it is not expressed in these neural population (Figure S1m–p).

**Figure 8:**
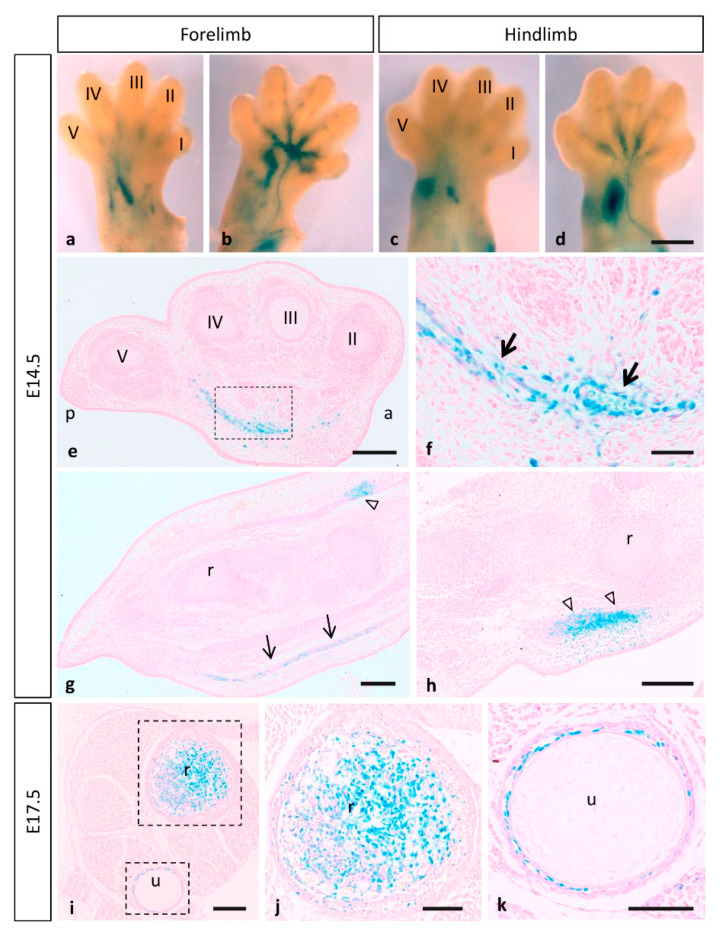
Mt1-mmp^LacZ/+^ distribution during mouse limb development. Whole mount forelimbs ((**a**), dorsal side; (**b**), ventral side) and hindlimbs ((**c**), dorsal side; (**d**), ventral side) stained with β-gal show MT1-MMP expression restricted to the vasculature and muscles of the limb at E14.5. (**e**–**h**) Paraffin cross (**e**,**f**) and longitudinal sections (**g**,**h**) through the hindlimb showed β-gal positive cells in endothelial cells of blood vessels entering the limbs (arrows in (**f**,**g**)) and dorsally in the earliest muscle blocks (open arrowheads in (**g**,**h**)) at E14.5. (**i**–**k**) Limb crossed sections at the level of proximal radius and ulna revealed LacZ expressing cells related to the ossification within the cartilage primordium of these bones at E17.5. Abbreviations: a, anterior; p, posterior; r, radius; u, ulna; digits I, II, III, IV and V. Scale bars: 500 µm (**a**–**d**), 200 µm (**e**,**g**–**i**), 100 µm (**j**,**k**), 50 µm (**f**).

Supporting these data, the expression of the protein MT1-MMP in the brain was confirmed by Western blot analysis in the olfactory bulb, cerebral cortex, dorsal mesencephalon (superior colliculus) and the spinal cord of E14.5 WT embryos (Figure 3). The highest levels of MT1-MMP were detected in the cerebral cortex, followed by the mesencephalon and the spinal cord, whereas the lowest levels were expressed in the olfactory bulb (Figure 3B). As expected, no protein levels were detected in the KO tissues while lower levels of protein were present in the heterozygous embryos. Notably, protein expression levels are as high in the cerebral cortex as in the forelimbs which is also supported by means of β-gal staining (Figure 8). Immunohistochemistry for β-gal and MT1-MMP in HT embryos at E14.5 revealed that the enzyme is transcribed preferentially in the proliferative zone of these brain regions (Figure 6). Thus, the highest levels of β-gal were detected in the subventricular zone whereas its expression is downregulated in the glomerular and mitral cell layers of the olfactory bulb (Figure 6a–c). On the contrary, the mature protein mainly localizes in the glomerular layer where double positive cells for β-gal and MT1-MMP are detected (Figure 6d–f). Similarly, some β-gal/MT1-MMP-positive cells were detected in the superficial layers of the superior colliculus and the mantle zone of the cerebral cortex, whereas the higher levels of β-gal also localize in the subventricular zone (Figure 6g–l).

MT1-MMP positive signal at the level of NCCs is in the direction of previous findings. In frog embryos, knock-down of MT1-MMP affected melanoblast migration, whereas MT3-MMP overexpression led to neural and head abnormalities, further suggesting their possible involvement in NCC development [80,81]. Also in Xenopus, MT1-MMP is expressed distinctively in NCCs and its function is paramount during EMT and migration independently of MMP-2 activation and possibly through the regulation of cadherin levels [23]. Thus, overexpression of MT1-MMP led to premature migration of NCCs, while down-regulation of the proteinase resulted in a reduced migration. Similarly, another member of the transmembrane MMPs, MT3-MMP, is expressed in cranial NCC in the chick embryo and its blockage inhibited NCC migration [82]. MT4-MMP expression has also been reported in NCC in the mouse embryo, and its orthologue mmp17b was demonstrated to be essential for the proper migration of NCCs in zebrafish [34,83].

Neural crest–derived cells are broadly distributed in adult tissues and organs, where a subset of them retain NCC-like multipotency [84]. Some of these derivatives include precursors for the development of the cardiac OFT, a process dependent on Smad4 expression on cardiac NCCs [11]. This process implies strong tissue remodeling through degradation and reorganization of ECM. Our results indicate that MT1-MMP is highly expressed in the OFT at these embryonic stages (Figure 1 and Figure 2), what suggests a possible role for MT1-MMP in tissue remodeling of the ECM for the proper OFT positioning, although the exact function of this proteinase in the OFT formation is to date not known. Previous work has shown that cardiac NCCs depleted of Smad4 show reduced mRNA levels of MT1-MMP which may explain defects in positioning and the recruitment of NCCs into the OFT in these mutants [11]. Smad regulates the transcription factors Snail and Slug, key effectors of EMT, tumor progression and invasiveness [85,86]. Despite the differing effects of both transcription factors on cell adhesion and integrin expression, both Snail and Slug up-regulate MT1-MMP in cancer cells suggesting a possible role of MT1-MMP in cardiac tissue remodeling during development through the TFG-beta pathway [87,88,89].

#### 3.2.1. Olfactory System

**Figure 9:**
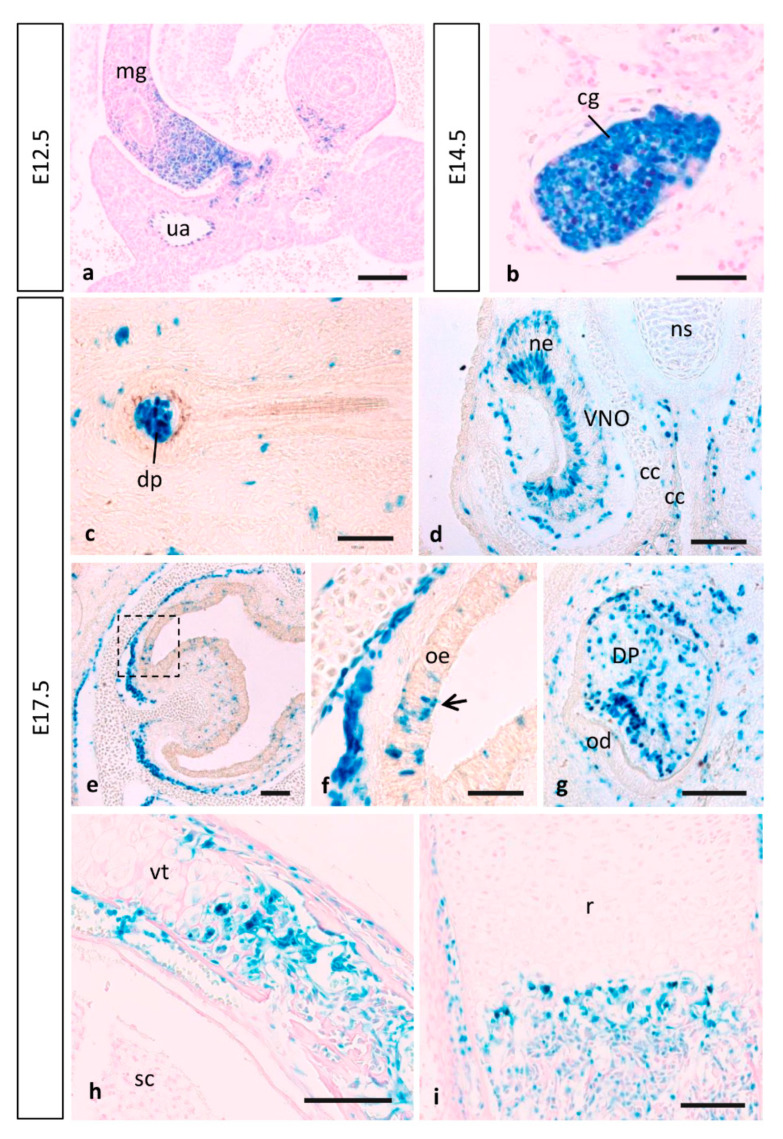
MT1-MMP expression in other mouse embryonic structures. Reporter expression in Mt1-mmp^LacZ/+^ early embryos was detected in the midgut loop and the cervical sensory ganglia. At E17.5, β-gal positive cells localized in the dermal papilla of the hair follicle (**c**), in sensory neurons of the vomeronasal organ (**d**) and the olfactory epithelium (**e**,**f**), and also in the condensing dental mesenchyme. Notably, MT1-MMP expression was also detected during endochondral ossification in the vertebra (**h**) and the radius of the hindlimb (**i**) by this stage. Abbreviations: cc, cartilaginous capsule; cg, cervical sensory ganglia; dp, dermal papillae; DP, dental pulp; mg, midgut loop; ne, neurosensory epithelium; ns, nasal septum; od, odontoblast; oe, olfactory epithelium; r, radius; sc, spinal cord; ua, umbilical artery; VNO, vomeronasal organ; vt, vertebra. Scale bars: 100 mm (**a**,**d**,**e**,**g**–**i**), 50 mm (**b**,**c**,**f**).

As mentioned before, our results show high levels of expression of MT1-MMP in the olfactory bulb from the early embryo to perinatal stages of development (Figure 5a,b,g,h,k,o). These data are in line with a recent study that reported proteinase expression in the postnatal olfactory bulb [27]. We have also shown β-galactosidase activity localized during development of distinct sensory organs such the cervical sensory ganglia (Figure 9b and Figure S2), the olfactory epithelium (Figure 9e,f) and the vomeronasal organ (VNO) (Figure 9d), the later containing receptor neurons that enable pheromone detection. Our results suggest that MMPs may be required in the olfactory epithelium since early stages of development to organize their connectivity between targets and NCC derivatives as they migrate. Previous work has established that MMP-2, MT1-MMP and TIMP-2 are temporally expressed during olfactory degeneration, neurogenesis and axonal outgrowth following injury in the mice olfactory epithelium, but to date there has been no previous description of expression of MT1-MMP in that tissue [90]. On the other hand, since MT1-MMP was detected in proliferating neurons of the neurosensory epithelium and in specific neurosensory organs we cannot rule out that MT1-MMP is relevant for the overall development of the olfactory system.

#### 3.2.2. Eye Development

MT1-MMP is expressed in the retina of healthy adult mice. As is the case for other angiogenesis related genes, it is localized in the nerve fiber layer of the postnatal mouse retina, where its expression correlates with inner retinal vascularization [29]. In fact, defective MT1-MMP functioning has been linked to several neurodegenerative disorders due to its role in neovascularization [33]. Previous studies have described expression of MT1-MMP in the eye. Thus, the proteinase has been detected in the inner retinal layer of adult rabbits, in adult human and monkey optic nerve and in the nerve fiber layer of the postnatal mouse retina [29,30,31,32]. However, the use of immunohistochemistry techniques with different commercial antibodies came with unclear results, and MT1-MMP localization has not been reported in the embryonic eye. Our data showed that, during eye development, the first Mt1-mmp^LacZ/+^ cells observed are endothelial cells of the hyaloid artery at E11.5 and E12.5 (Figure 7d,e). Moreover, β-gal positive cells were detected in the central retina and the lens by E12.5 (Figure 7e). Later in development, LacZ reporter expression was stronger in the hyaloid vessels but absent from the lens and the neural retina (Figure 7f). All these data support a role for this metalloproteinase during angiogenesis and proliferation of RGC at early embryonic stages likely by its expression in the nervous component of the retina since Mt1-mmp gene was not found to be enriched in the microarray assays performed in retinal tip cells [91]. Our findings also suggest that MT1-MMP may contribute to regression of the hyaloid vasculature that occurs after birth in accordance with previous actions of MT1-MMP in vessel regression suggested in vitro [92].

In a relevant way, we found that MT1-MMP localized at distinct points of the visual pathway, as LacZ staining was detected in optic chiasm region and the optic stalk at early stages of development. Notably, numerous positive cells for β-gal were found in the optic disc and the optic nerve, as well as in the optic chiasm region, possibly corresponding to astrocytes, by E17.5 (Figure 5p and Figure 7g). Expression was first detected in the caudal mesencephalon in Mt1-mmp^LacZ^ knock-in embryos at E11.5 and 12.5 (Figure 1j,l and Figure 4a,b). As shown in whole mount embryos and brain, LacZ reporter expression highly increased later in development in the dorsal mesencephalon at E14.5 and the superior colliculus at E17.5 (Figure 4d,g–l). In fact, analysis of horizontal and coronal sections from these embryos revealed that β-gal positive cells were localized in the superficial layers of the mesencephalic tectum and superior colliculus that receive direct visual input from the retina (Figure 5i,l). Interestingly, MT1-MMP expression has been described in mice RGC axons and their growth cones as well as in glial cells [93]. In zebrafish, MT1-MMP has been reported to be necessary for retinal and retinotectal development, being expressed in RGCs and the optic nerve [94]. Despite all these data, there is no clear evidence for a role of MT1-MMP in the developing mouse visual system and further studies exploring its function in the retina are needed. Previous work developed in our lab have shown that matrix metalloproteinases such as MT4-MMP may play a role during retinal development [34]. The present data together with our previous findings suggest generally that MMPs, and in particular MT-MMPs, may play complementary or redundant roles in the development of the eye, possibly through regulation of the axonal migration and elongation through mechanisms to date unknown.

MMP activities have been linked to several neurodegenerative disorders of the retina. For instance, expression of MT1-MMP has been reported in astrocytes of the human optic nerve head and increased levels of the protease have been related to glaucoma [31]. As mentioned before, we also detected high expression levels of MT1-MMP in the optic disc and nerve at perinatal stages. In line with this, the expression pattern of MT1-MMP further suggests its implication in axonal outgrowth during development, which may as well suggest a possible role in repair. Previous studies have highlighted its role in retinal remodeling and axonal regeneration upon injury of the optic nerve in zebrafish. Thus, after an initial peak in the proteinase expression during the injury response phase, retinal MT1-MMP gradually decreases during axonal regeneration [95]. It is known that MMPs play a role as beneficial factors in axonal regeneration, where MT1-MMP dependent activation of MMP-2 contributes RGCs axonal outgrowth [93].

### 3.3. Expression of MT1-MMP during Limb Development

During limb development, β-gal staining shows that MT1-MMP expression was restricted to the vasculature and muscles and tendons of the forelimbs and hindlimbs of E14.5 embryos (Figure 8a–h). Thus, paraffin longitudinal and cross sections through the hindlimb showed β-gal positive endothelial cells of blood vessels entering the limbs ventrally (Figure 8e–g). The reporter expression was also localized dorsally in the earliest muscle blocks and tendons at E14.5 (Figure 8g,h). Notably, limb coronal sections at the level of proximal radius and ulna, revealed LacZ expressing cells related to the ossification within the cartilage primordium of these bones at E17.5 (Figure 8i–k). In accordance with our data, defective vascular invasion of cartilage was reported in MT1-MMP-deficient mouse neonates as well as mRNA levels were reported in muscle tissue of newborn mice [13,20]. These results were confirmed by Western blot analysis (Figure 3). Consistent with our data on the LacZ reporter expression of our gene, high levels of expression of MT1-MMP were detected in the forelimbs of E14.5 WT embryos while protein levels were lower in the HT and absent in the KO embryonic tissue.

Our results are in line with previous evidence showing that MT1-MMP is prominently expressed in embryonic connective tissue cells such as osteoblasts, osteoclasts and perichondrial, muscle and tendon fibroblasts [17,96,97]. Knock-out MT1-MMP display several craniofacial abnormalities linked to shorter bones due to delayed vascular development as well as several skeletal and extra skeletal connective tissues disorders [13,24,25]. In addition, previous studies have revealed that double deficiency for MT1-MMP and MT3-MMP in mice leads to severe embryonic defects in palatogenesis and skeletal development incompatible with life [10]. All these effects are characteristic of lack of interstitial collagenolytic activity of MT1-MMP that results in generalized connective tissue abnormalities [13,24,25,98]. Regarding the molecular control during skeletal development, it has been demonstrated in culture conditions that MT1-MMP, which is produced by osteoblasts, activate latent TGF-beta to promote their survival during their conversion into osteocytes [99]. Moreover, MT1-MMP participates in bone remodeling by non-proteolytic modulation of Rac1 activity during osteoclast formation [100].

As previously reported, MT1-MMP activity is essential for secondary ossification during bone formation in the mouse embryo [101]. Supporting these data, we demonstrated the expression of the enzyme during the ossification within cartilage primordium of distinct bones including the mandible, hyoid bone, ribs, nasal septum and vertebra (Figure 9h,i). Since we also reported MT4-MMP expression in distinct bones during embryogenesis, we hypothesize that both proteases may be complementary and relevant to skeletal maturation [34].

Skeletal muscle development involves the migration of myogenic cells from somites, myoblast cell cycle arrest, myocyte fusion and finally differentiation into multinucleated contractile myofibers. Communication between cells and the surrounding microenvironment, including cell–cell and cell–matrix interactions, play a role in all phases of myogenesis [102]. The differentiation of myoblasts proceeds in a tissue environment surrounded by extracellular matrix that has to be remodeled dynamically in tune with the myogenic differentiation process. Once differentiated, myofibers form a functional contractile unit by integrating into the skeletal muscle framework. In that regard, previous work has shown that MT1-MMP transcripts were expressed by myoblast, and it was identified as a major contributor to complete myoblast cell fusion by its actions on fibronectin [21]. Furthermore, MMP-2 is expressed in myoblasts and appears to be activated by the cleavage performed by MT1-MMP enzyme [103].

As previously mentioned, the histochemical analysis of β-galactosidase activity revealed an expression at the intersomitic region from E10.5 to E12.5 compatible with intersomitic arteries. Nonetheless, this pattern of expression is also coincident with tendon tissue development spatially as well as temporally [104]. The tendon is an ECM-rich tissue that undergoes dynamic degradation of ECM at certain stages [105]. We hypothesize that MT1-MMP activity may contribute to the degradation of ECM for the formation of the lateral protrusions that tenocytes form to connect to adjacent cells [106]. It is known that MT1-MMP knock-out mice have arrested tendon development around the time of birth due to MT1-MMP processing of fibronectin rather than its collagenolytic activity [107]. This has led us to hypothesize a possible role of MT1 MMP in tendon development. Recently, MT1-MMP function has been reported to be essential to allow AChR clustering at the neuromuscular junction through local degradation of the extracellular matrix [108]. Altogether, these data suggest important physiological roles for MT1-MMP not only in the development of muscles and tendons but also at the synapse for the proper muscle innervation.

### 3.4. MT1-MMP Expression in Other Embryonic Structures

Apart from the high expression observed during the cardiovascular, brain and limb development, MT1-MMP also localized in other embryonic tissues as shown by LacZ staining. Skin appendages develop from placodes involving reciprocal interactions between the surface ectoderm and underlying mesenchyme during embryogenesis [109]. During the development of the hair follicle, MT1-MMP expression is initially detected in the epidermal placode at E14.5 (data not shown). At later embryonic stages, reciprocal induction between the placode and the dermal condensate will lead to the growth of the primordia of the hair follicle into the dermis. By these stages, MT1-MMP will have localized in the dermal papilla (Figure 9c).

As mentioned before, craniofacial defects present in MT1-MMP-null mice and MT1/MT3-MMP-double knock-out mice suggest a role of MT-MMPs in cranial morphogenesis [10,24]. Previous work has shown expression levels of MT1-MMP in the craniofacial mesenchyme, in condensations for the Meckel’s cartilage that later extend in the ossifying craniofacial skeleton [17]. Our data reveals expression levels of MT1-MMP in the teeth and mandible (Figure 9g). It is known that migrating NCCs contribute to the formation of the ectomesenchyme that ultimately will give rise to the formation of the developing tooth, comprising dynamic ECM production and modification and cell migration through ECM [110]. In that regard, it is known that MT1-MMP is widely expressed in the tooth and surrounding connective tissues during development and postnatal growth, and its activity in the dental mesenchyme is essential for proper tooth root formation and eruption [108,111]. MT1-MMP mRNA expression has been recently reported in cells associated with teeth and surrounding connective tissues during development [16,24]. Loss of MT1-MMP in mice impairs tooth root formation and eruption with multiple defects in dentoalveolar tissues, whereas MT1-MMP-deficient mice feature loss of molar tooth eruption and root formation [24,26]. On this subject, our studies point to an indispensable role for MT1-MMP-mediated matrix remodeling in tooth eruption through effects on bone formation, soft tissue remodeling and organization of the follicle/PDL (periodontal ligament) region.

In the present work, the histochemical analysis of β-gal also revealed that expression was very strong in the proximal part of the midgut loop within the physiological umbilical hernia as well as in the associated umbilical artery and vein from 12.5 to E17.5 (Figure 9a). This expression is found in an anatomic area where ECM remodeling is key to both rotation of the midgut as well as retraction of the herniated loops. Previous studies have pointed out a correlation between MT1-MMP and intestinal villi morphogenesis. MT1-MMP labeling was observed in the entire villus epithelium from birth until the complete maturation of the small intestinal mucosa in rats and chickens [42]. Further, MMP-2 activity plays a role during rat colonic gland formation, and this activity is regulated at both gene transcription and proenzyme activation levels [112]. No evidence to date has shown a similar expression pattern in the mouse embryo. In the present work, we show that β-gal staining was observed in the developing intestine from E14.5, becoming stronger at stage E17.5, which is equivalent to reported results in chicken embryos [42].

## 4. Conclusions

In summary, in this study we reported the expression of the transmembrane proteinase MT1-MMP at different developmental stages of the mouse embryo. We identify a novel pattern of expression during nervous system development that suggest key functions for this proteinase in axon growth and neuronal proliferation/differentiation. Besides our present data point for a possible role of MT1-MMP in endocardial differentiation during heart morphogenesis as well as in endothelial cell migration during angiogenesis. The activity of MT1-MMP is also relevant during limb development. The molecular mechanisms essentially remain unknown, but the accompanying co-expression in most territories of the inhibitor TIMP2 and its target MMP-2 indicates that the proteinase activity participates on pericellular matrix degradation and processing of distinct cell surface receptors and adhesion molecules that may allow cell migration, axon growth and angiogenesis, all of them essential processes in embryogenesis. Future studies are needed to elucidate the actual role of MT1-MMP in these cellular processes and the mechanisms involved during embryonic development.

## Data Availability

Not applicable.

## Supplementary Materials

The following are available online at https://www.mdpi.com/article/10.3390/cells10092448/s1, Figure S1: MT1-MMP is expressed in endothelial cells of distinct embryonic tissues. Figure S2: MT1-MMP is expressed in sensory ganglia during mouse development. Figure S3: Representative western-blot EMS.
