# Supplementary material for: Dynamic Expression of Membrane Type 1-Matrix Metalloproteinase (Mt1-mmp/Mmp14) in the Mouse Embryo
Source: Cells. 2021 Sep 17;10(9):2448. doi: 10.3390/cells10092448 (PMC8465375; doi:10.3390/cells10092448)
Supplement: Supplementary file 1 [file cells-10-02448-s001.zip › Sumplemmentary Material/Figure 3. Representative western-blot EMS 03 Sept 2021 EMS.pdf]

A

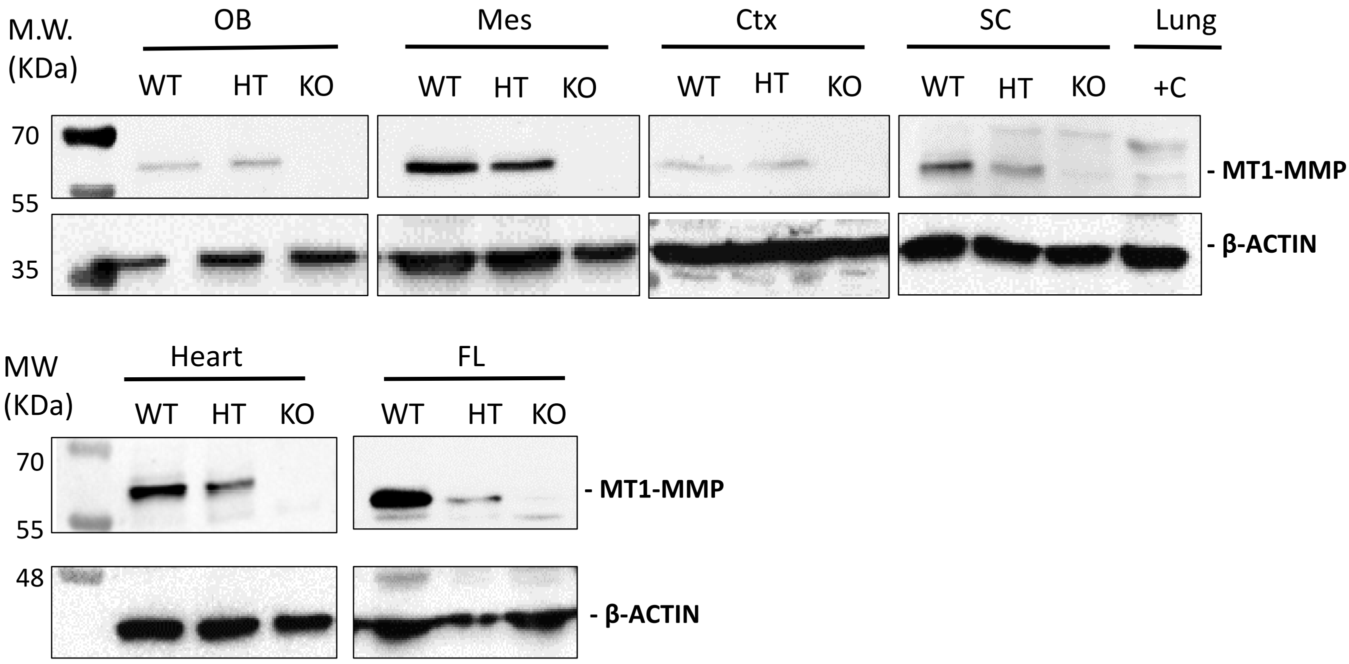
M.W. (KDa)
OB
Mes
Ctx
SC
Lung
WT HT KO
WT HT KO
WT HT KO
WT HT KO
+C
70
55
35
- MT1-MMP
- β-ACTIN
MW (KDa)
Heart
FL
WT HT KO
WT HT KO
70
55
48
- MT1-MMP
- β-ACTIN

B

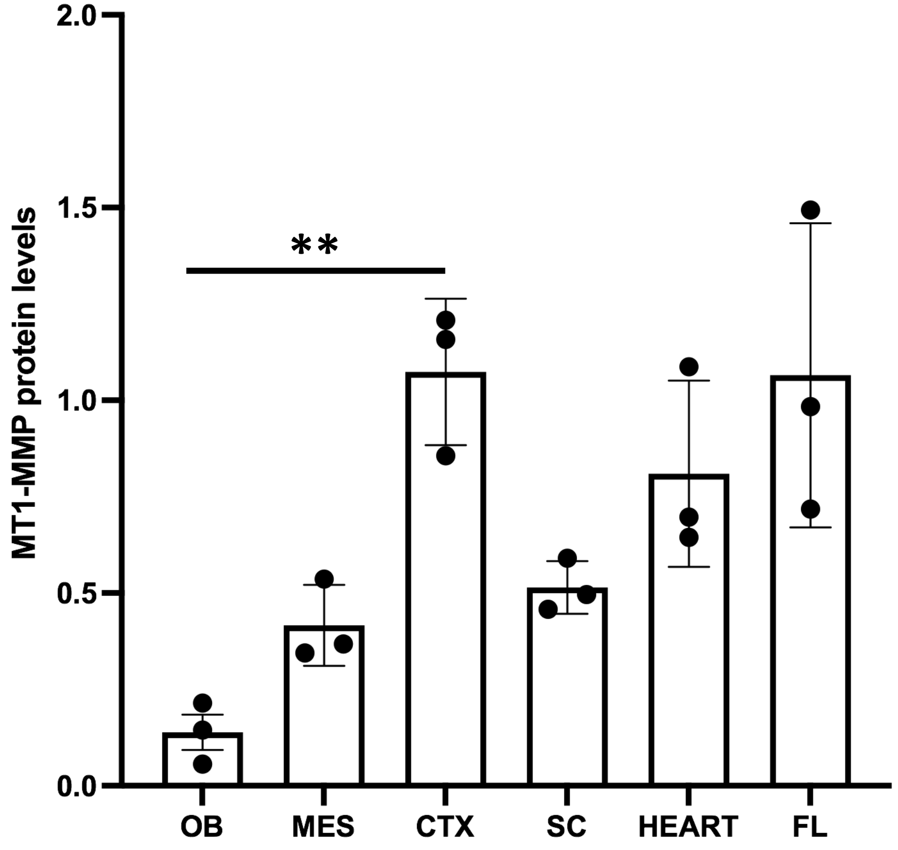
MT1-MMP protein levels
2.0
1.5
1.0
0.5
0.0
**
OB
MES
CTX
SC
HEART
FL

Included in the composition

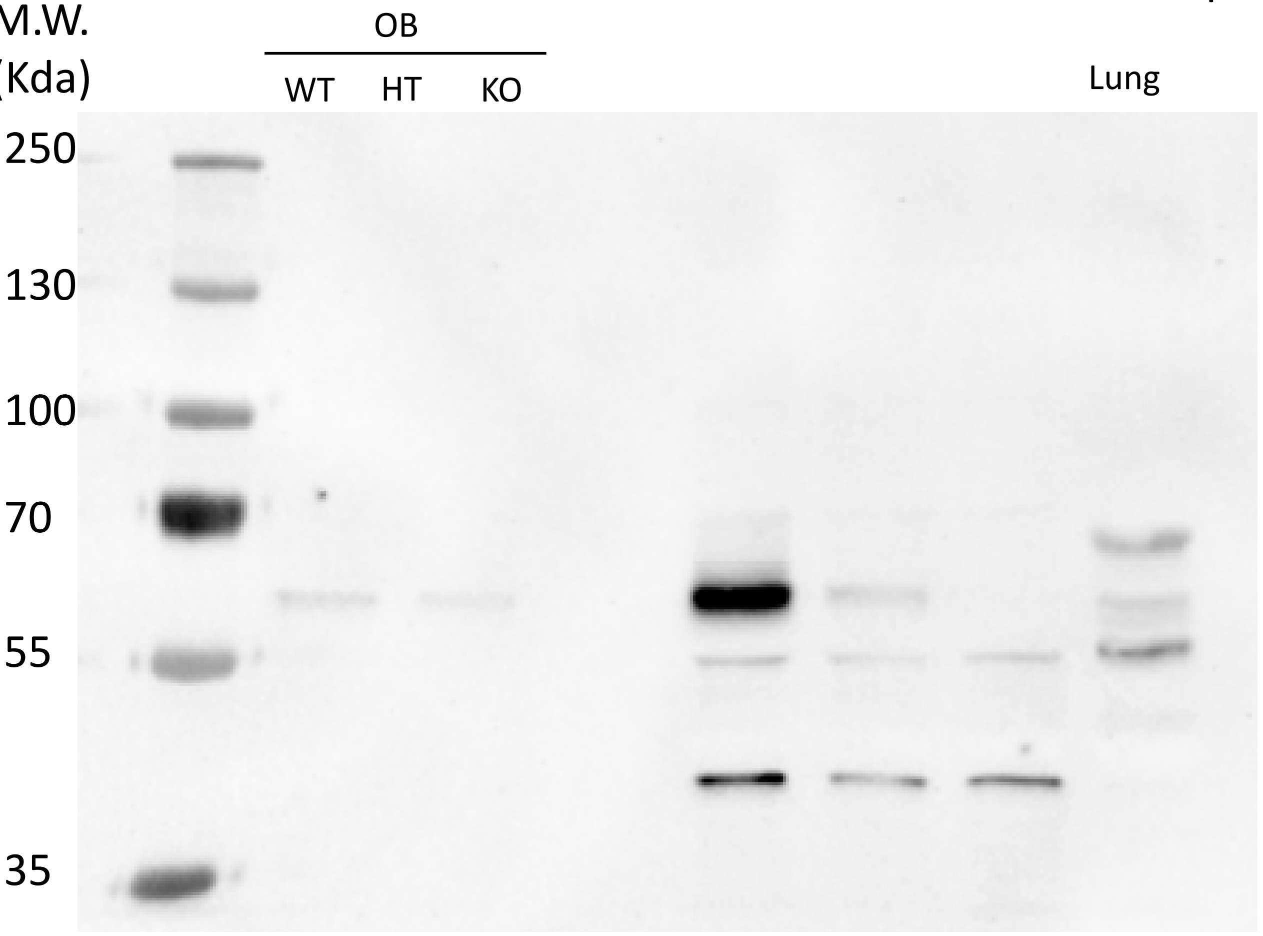

AB 1º: MT-LOOP [EP1264Y] (1:2500)
Ab 2º: goat anti- rabbit HRP (1:2500)

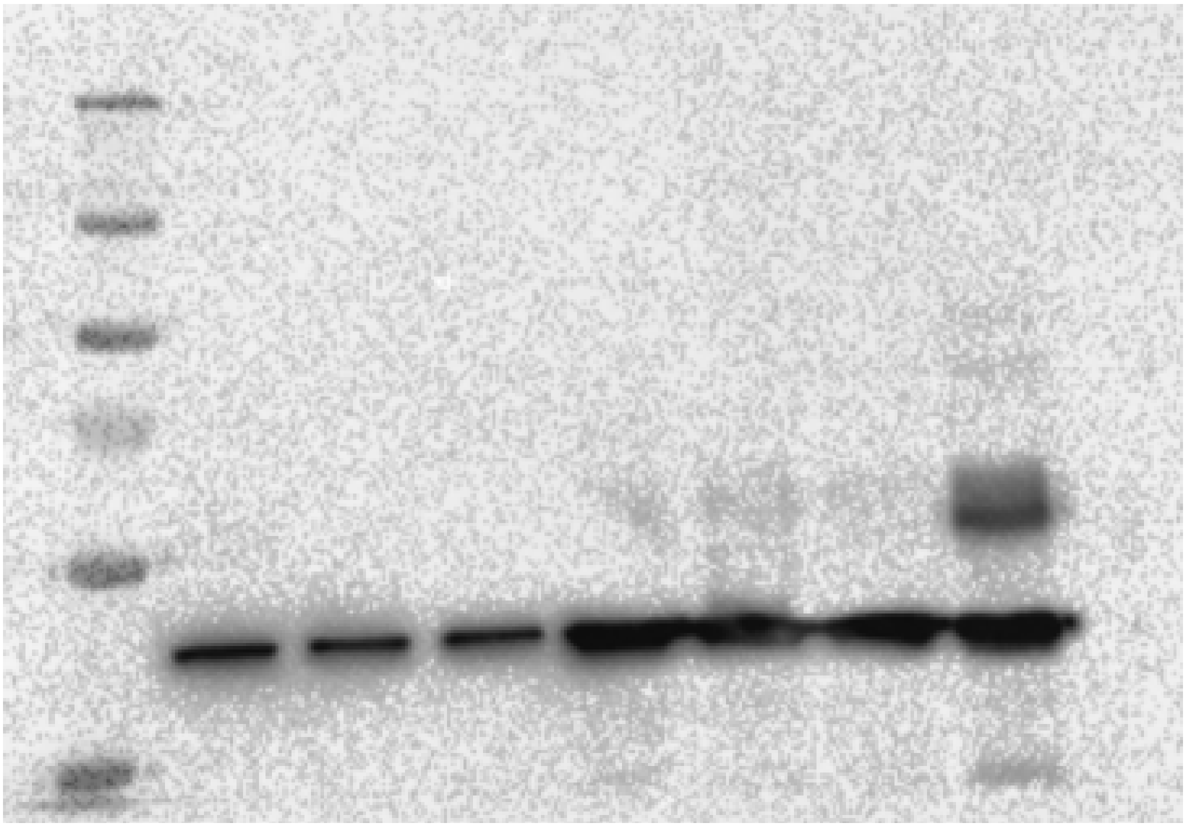

AB1º: monoclonal β-actin mouse (1:1000)
Ab 2º: goat anti-mouse HRP (1:2500)

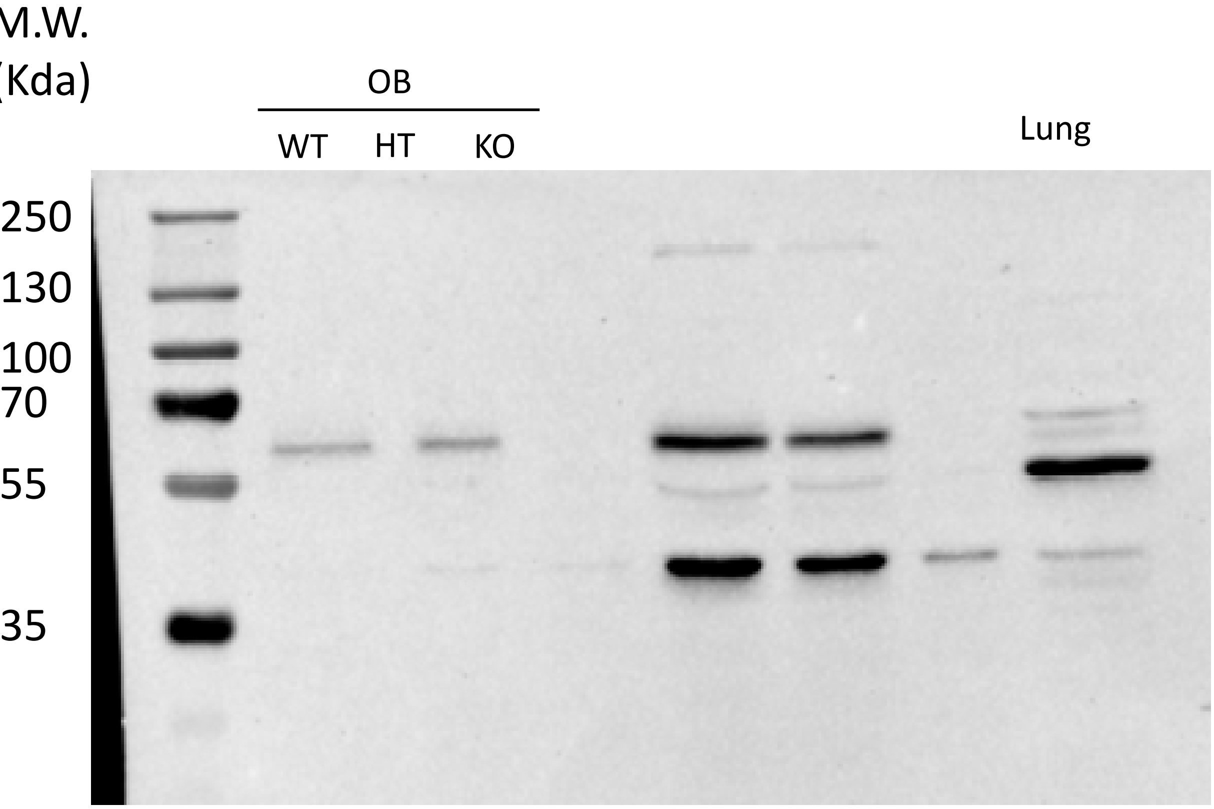

AB 1º: MT-LOOP [EP1264Y] (1:2500)
Ab 2º: goat anti- rabbit HRP (1:2500)

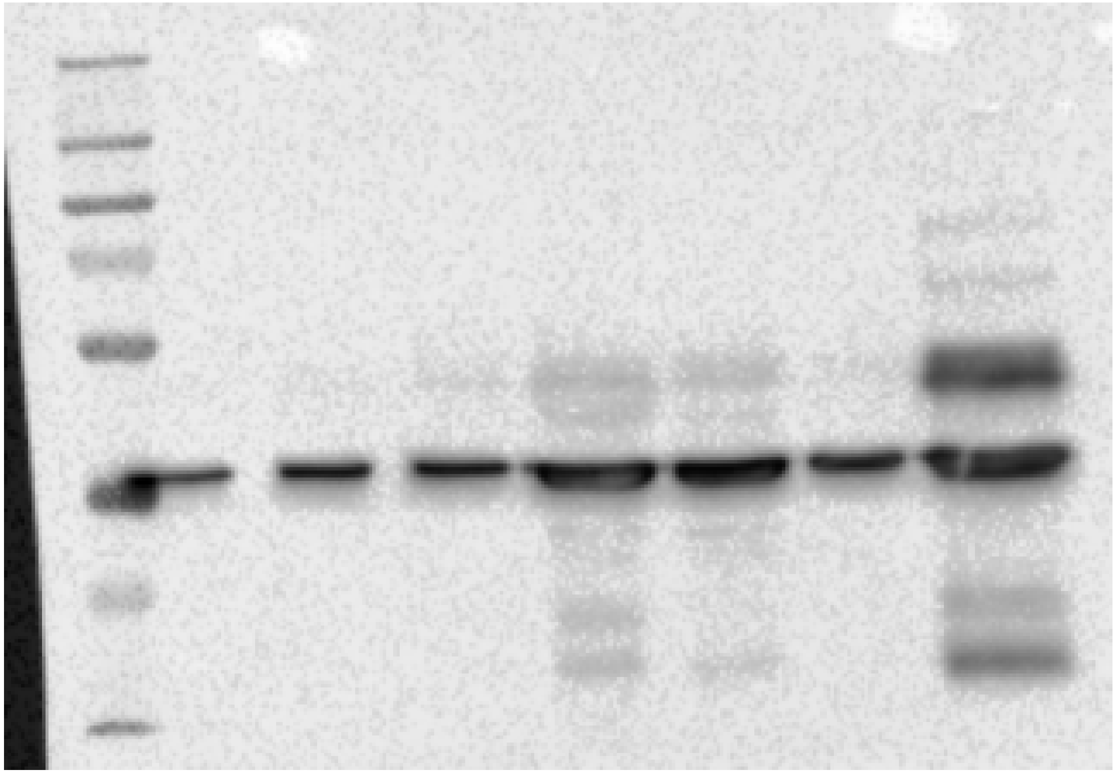

AB1º: monoclonal β-actin mouse (1:1000)
Ab 2º: goat anti-mouse HRP (1:2500)

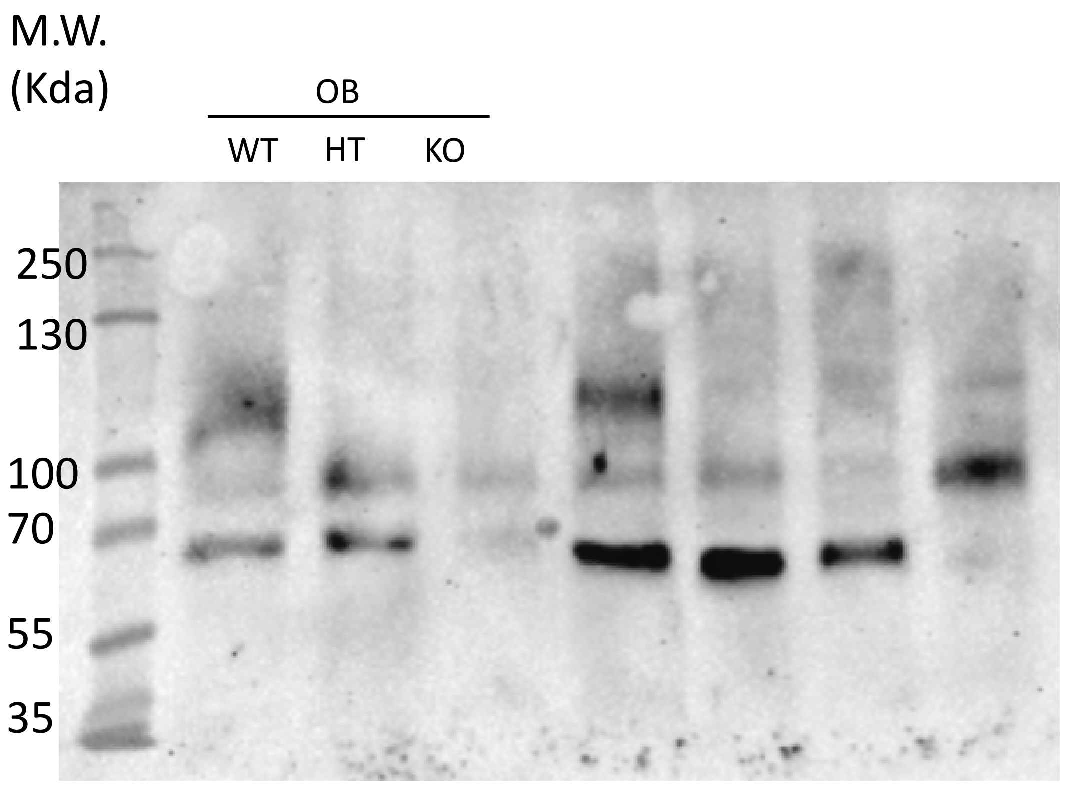

AB 1º: MT-LOOP [EP1264Y] (1:2500)
Ab 2º: goat anti- rabbit HRP (1:2500)

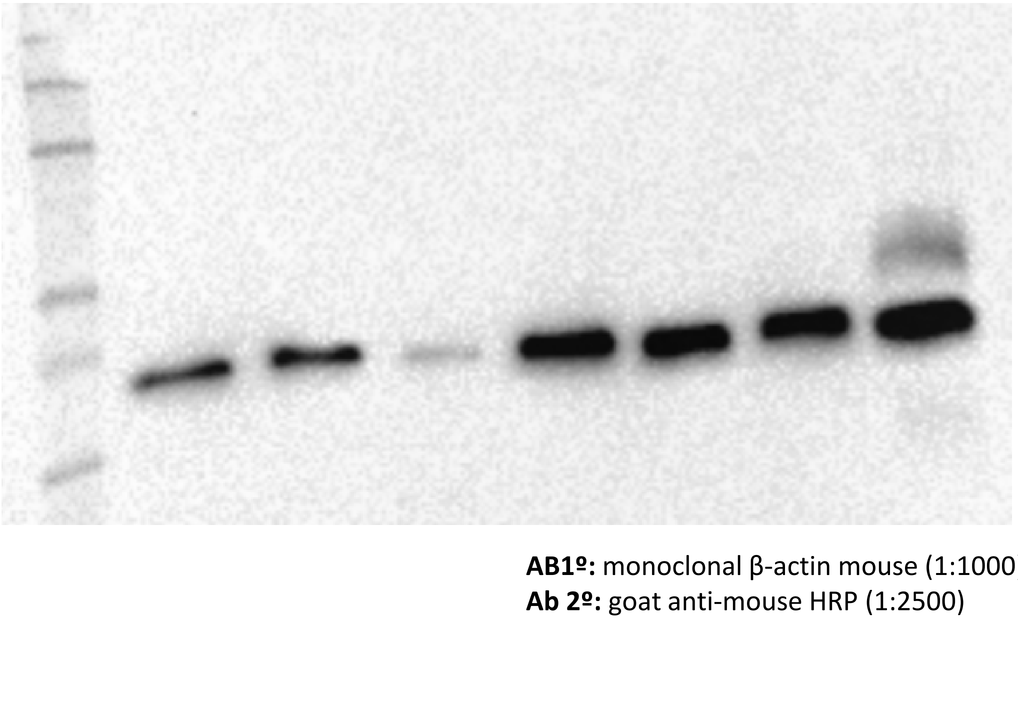

AB1º: monoclonal β-actin mouse (1:1000)
Ab 2º: goat anti-mouse HRP (1:2500)

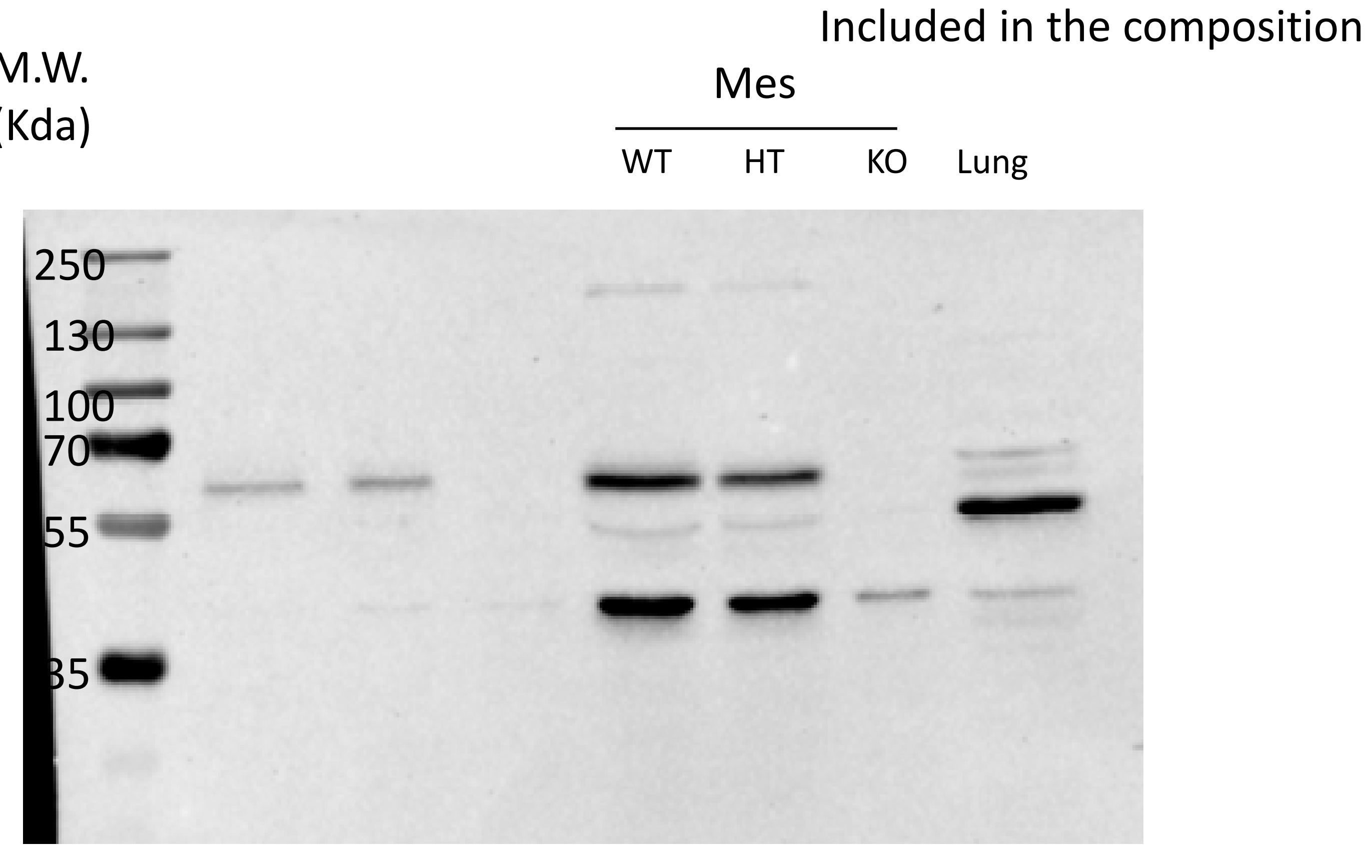

AB 1º: MT-LOOP [EP1264Y] (1:2500)
Ab 2º: goat anti- rabbit HRP (1:2500)

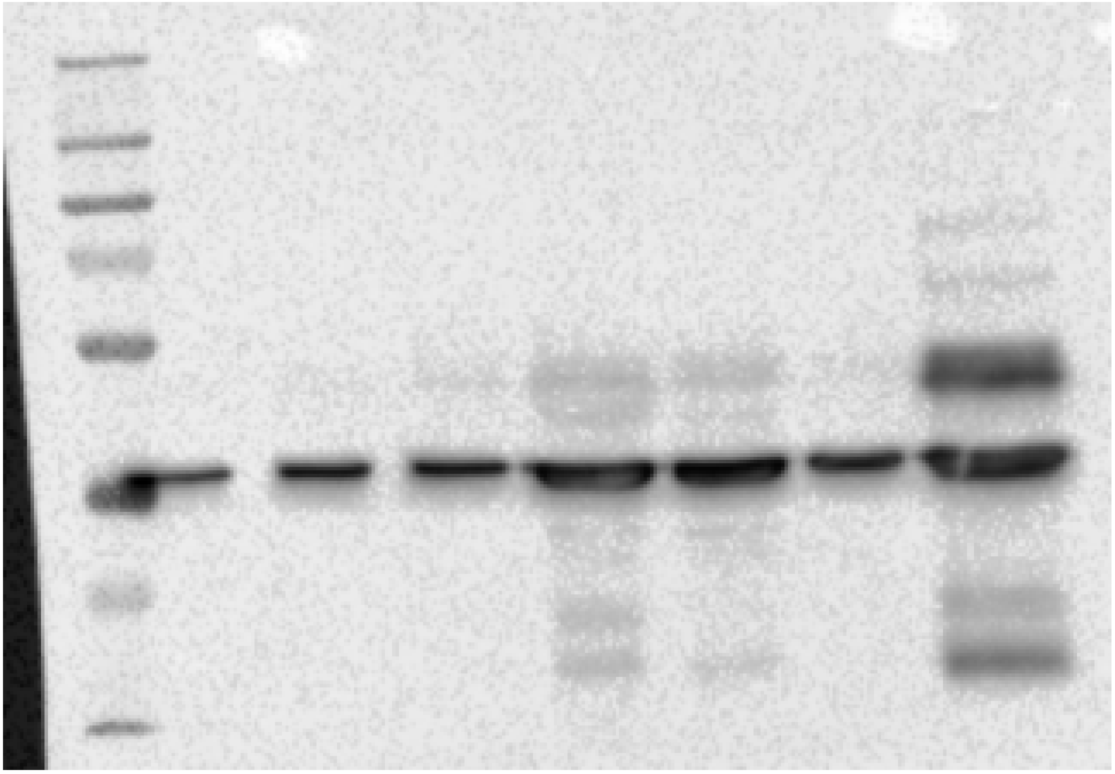

AB1º: monoclonal β-actin mouse (1:1000)
Ab 2º: goat anti-mouse HRP (1:2500)

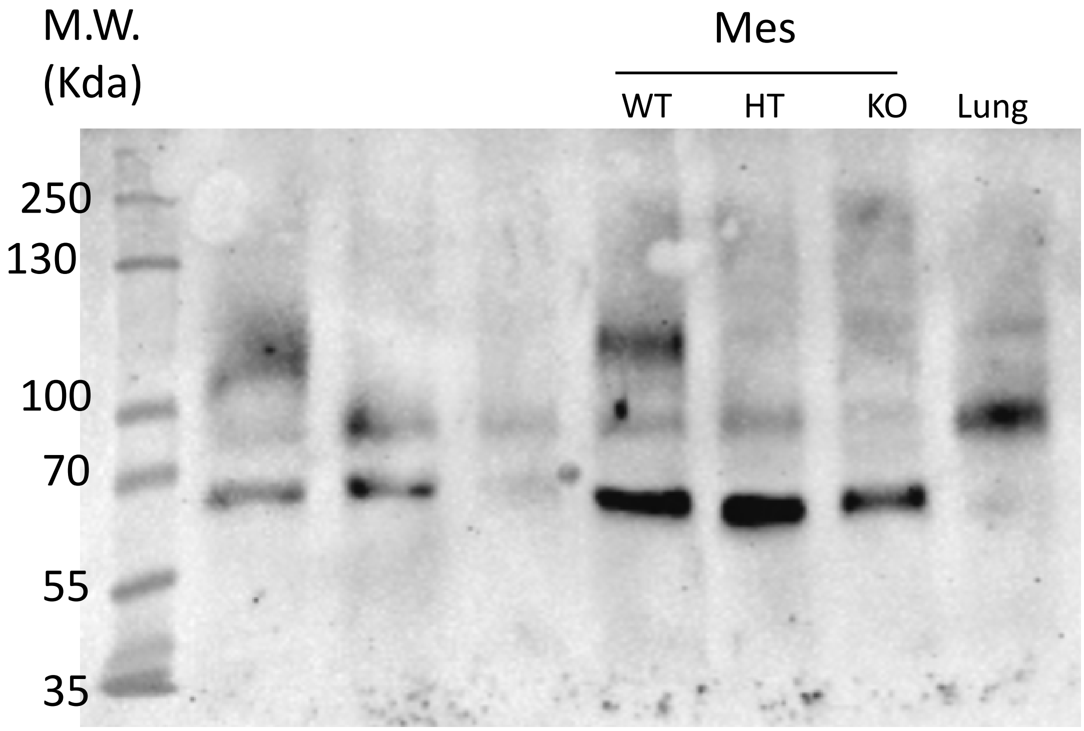

AB 1º: MT-LOOP [EP1264Y] (1:2500)
Ab 2º: goat anti- rabbit HRP (1:2500)

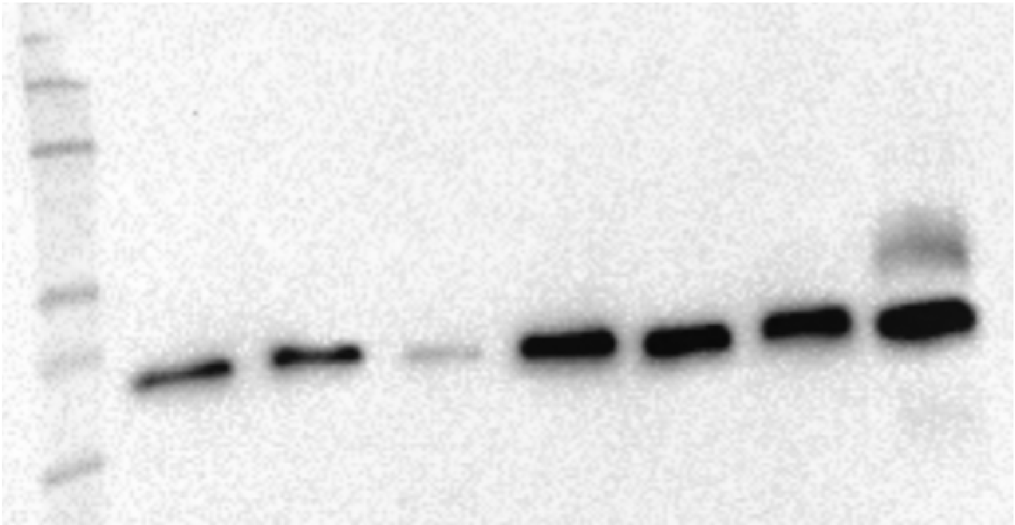

AB1º: monoclonal β-actin mouse (1:1000)
Ab 2º: goat anti-mouse HRP (1:2500)

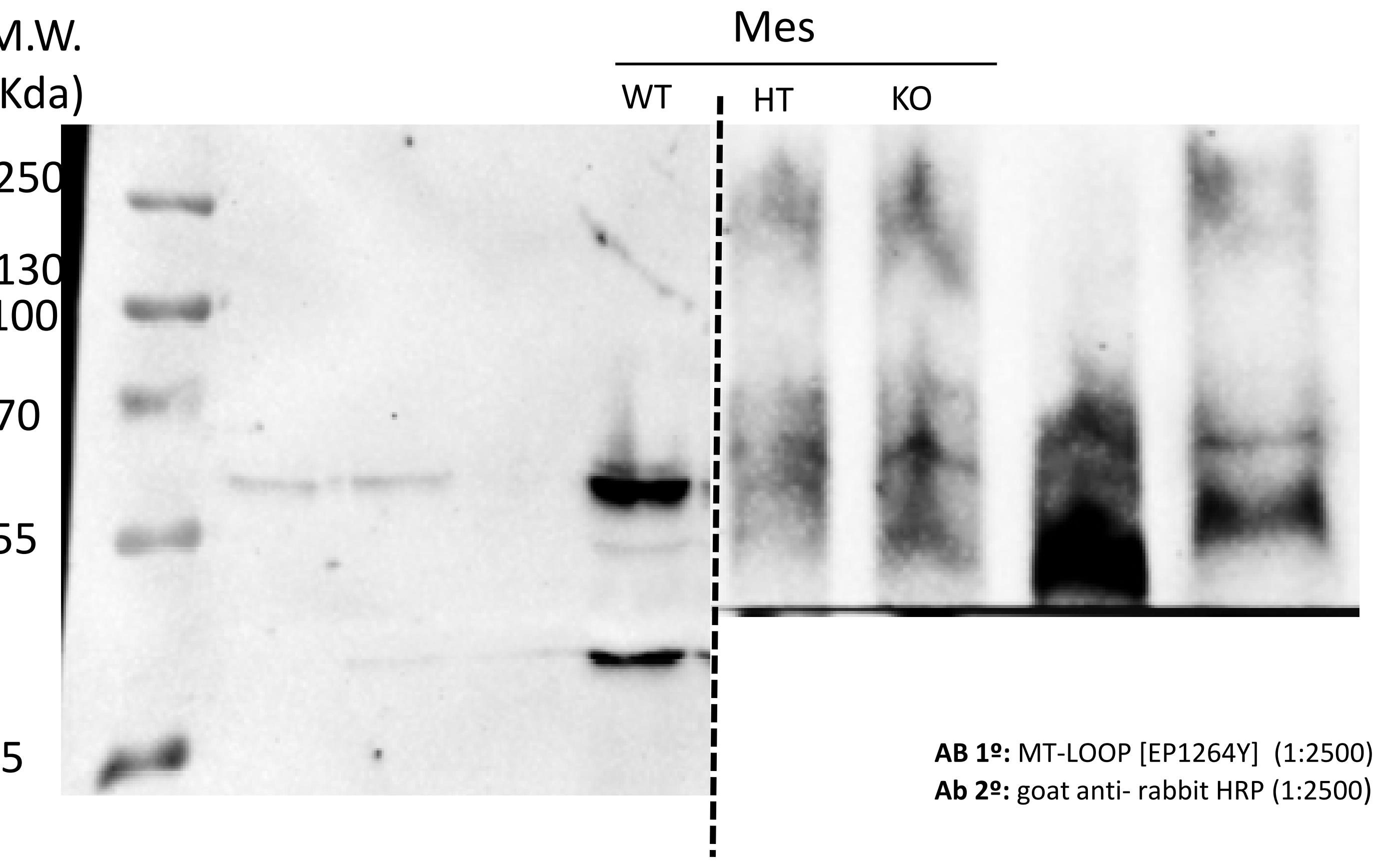
M.W.
(Kda)
250
130
100
70
55
5
Mes
WT
HT
KO
AB 1º: MT-LOOP [EP1264Y] (1:2500)
Ab 2º: goat anti- rabbit HRP (1:2500)

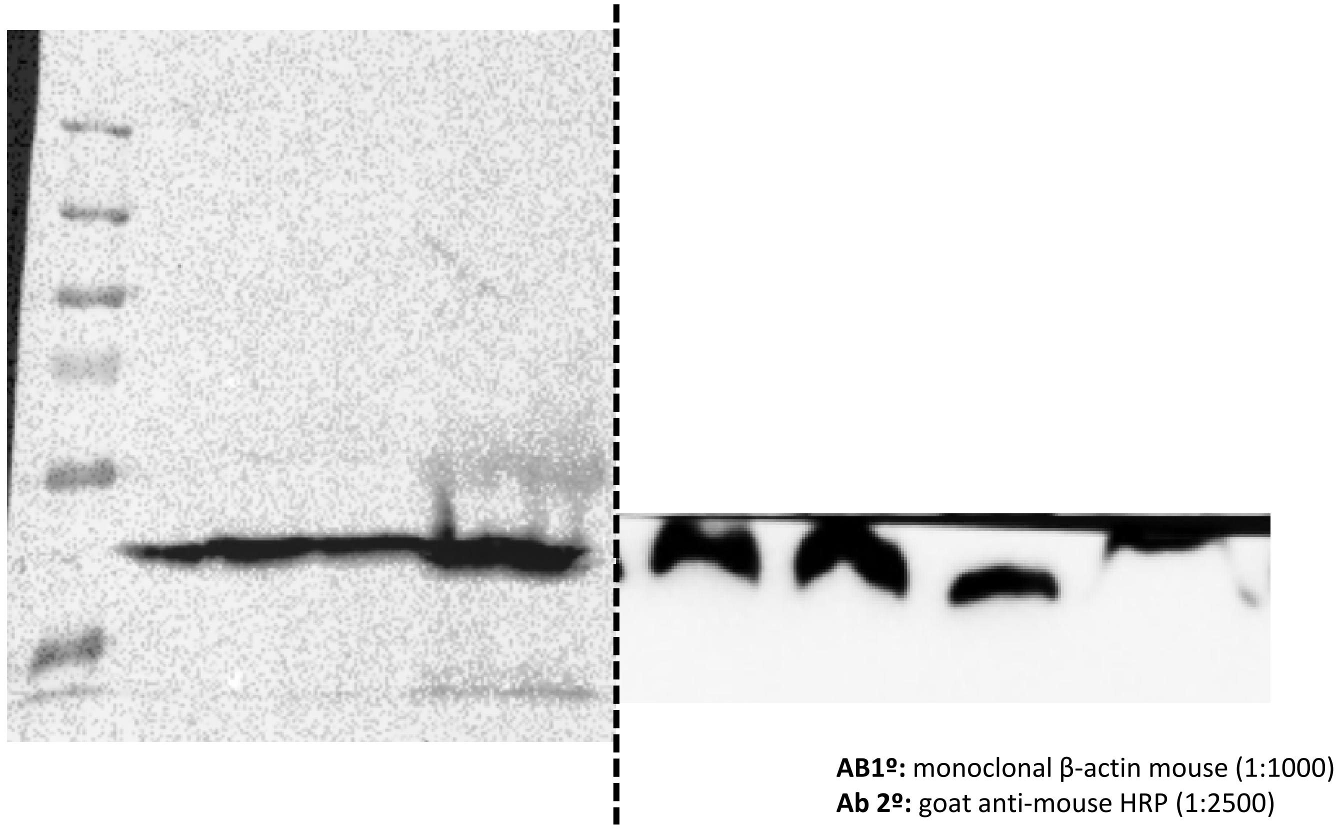
AB1º: monoclonal β-actin mouse (1:1000)
Ab 2º: goat anti-mouse HRP (1:2500)

Included in the composition

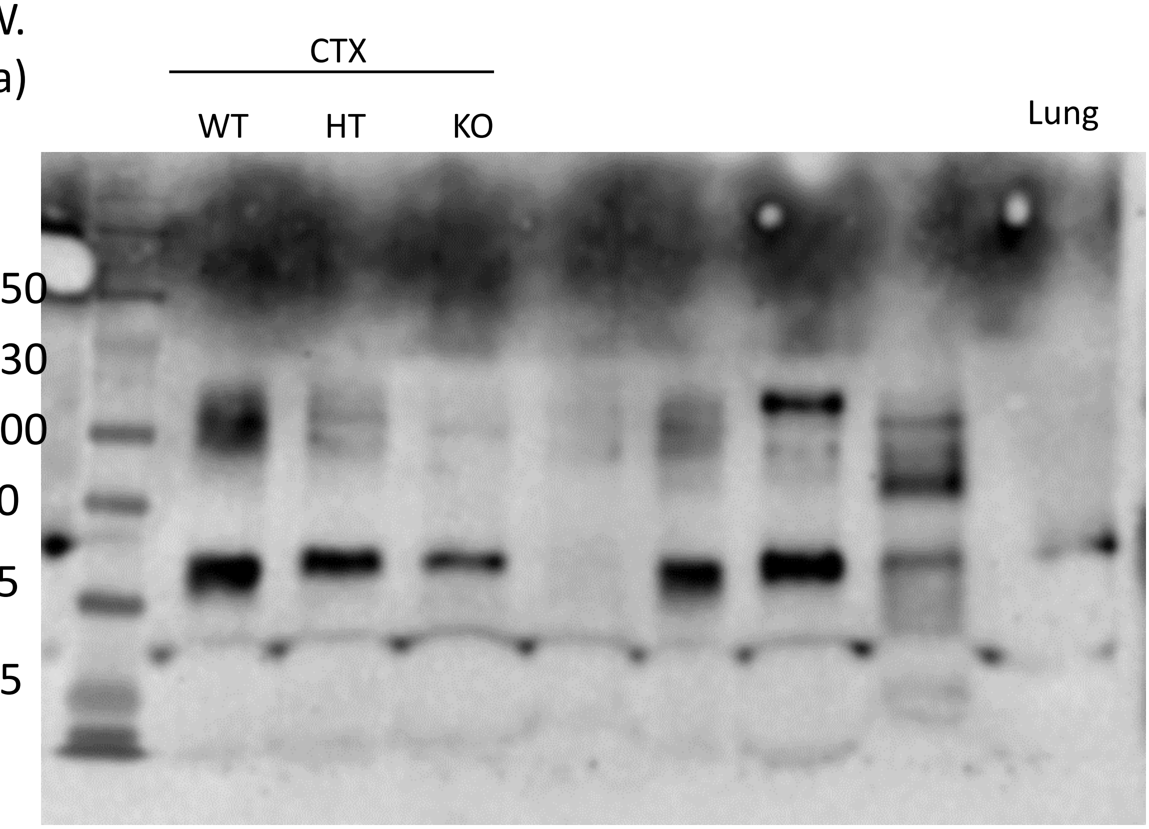

AB 1º: MT-LOOP [EP1264Y] (1:2500)
Ab 2º: goat anti- rabbit HRP (1:2500)

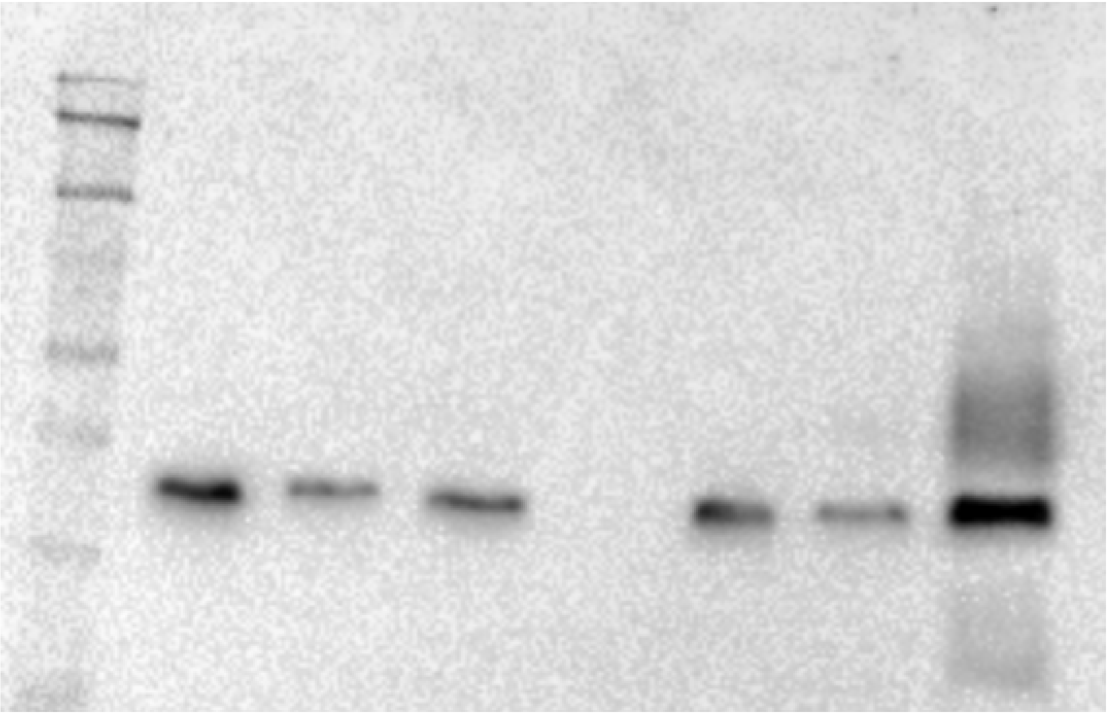

AB1º: monoclonal β-actin mouse (1:1000)
Ab 2º: goat anti-mouse HRP (1:2500)

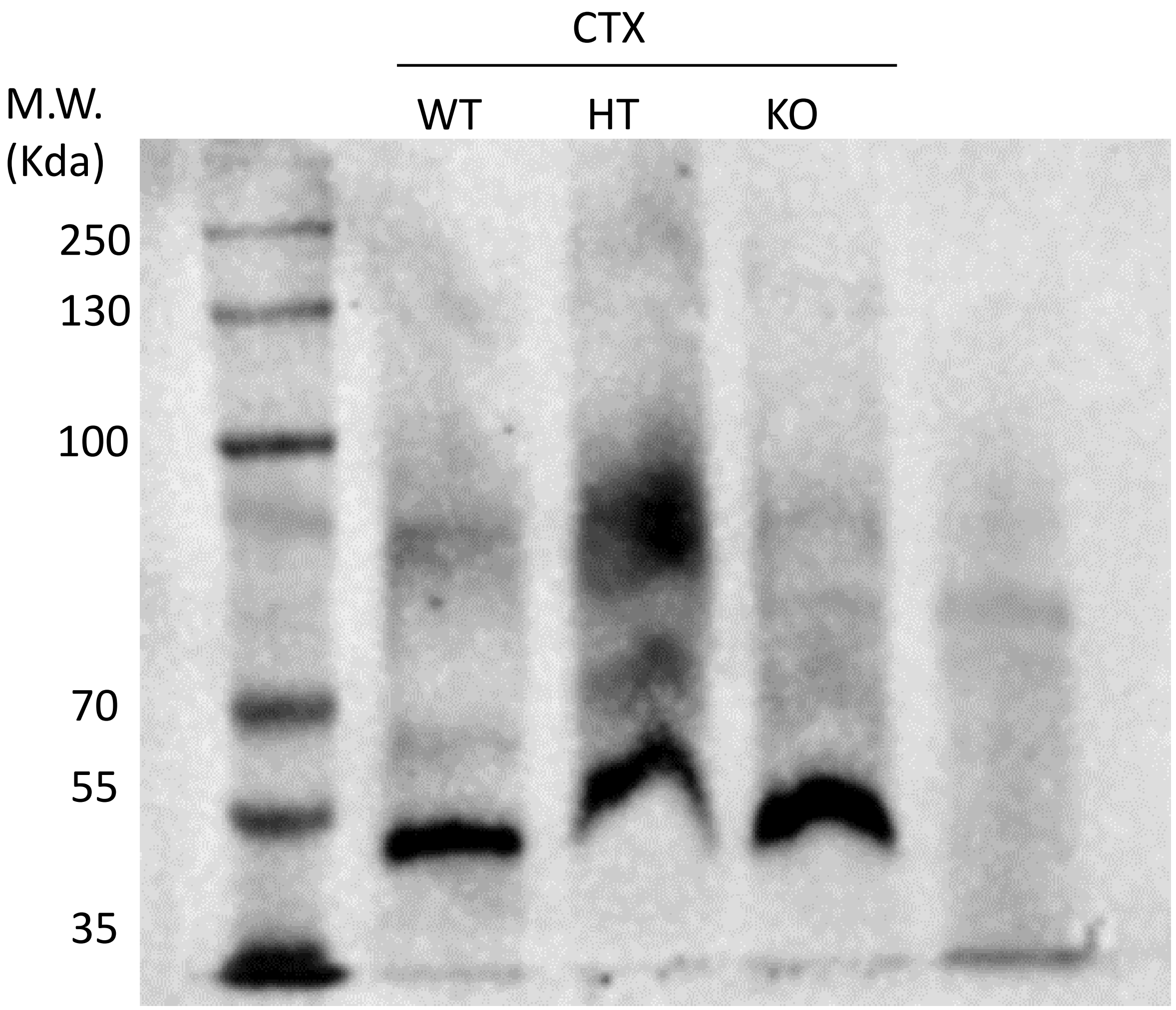

AB 1º: MT-LOOP [EP1264Y] (1:2500)
Ab 2º: goat anti- rabbit HRP (1:2500)

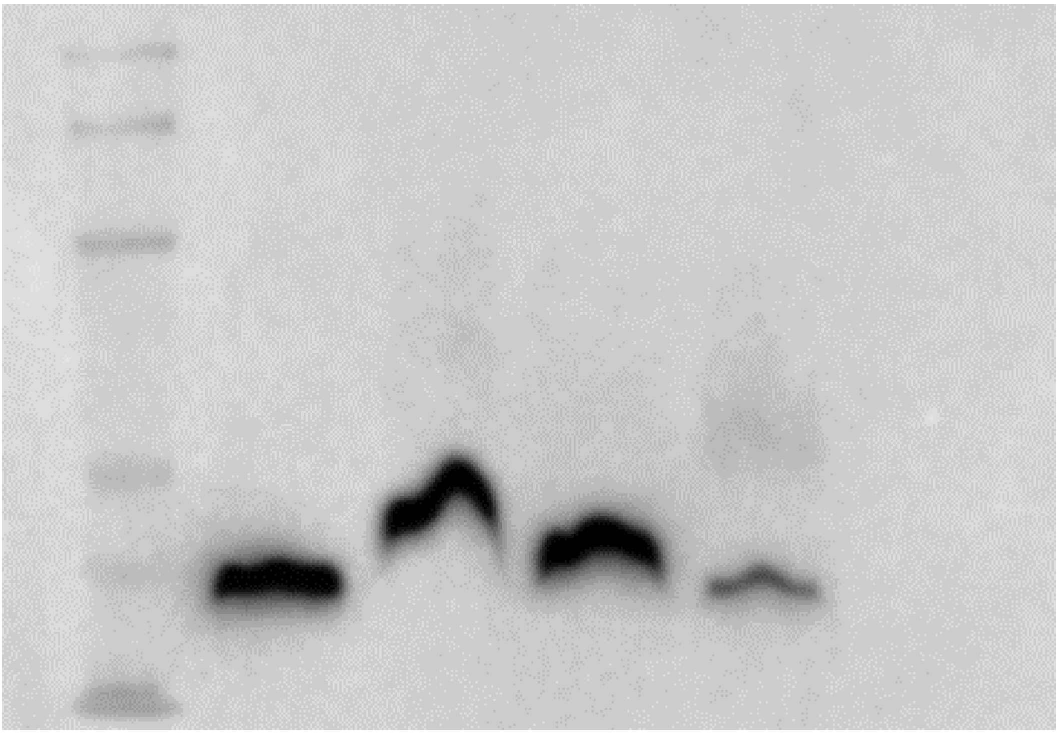

AB 1º: MT-LOOP [EP1264Y] (1:2500)
Ab 2º: goat anti- rabbit HRP (1:2500)

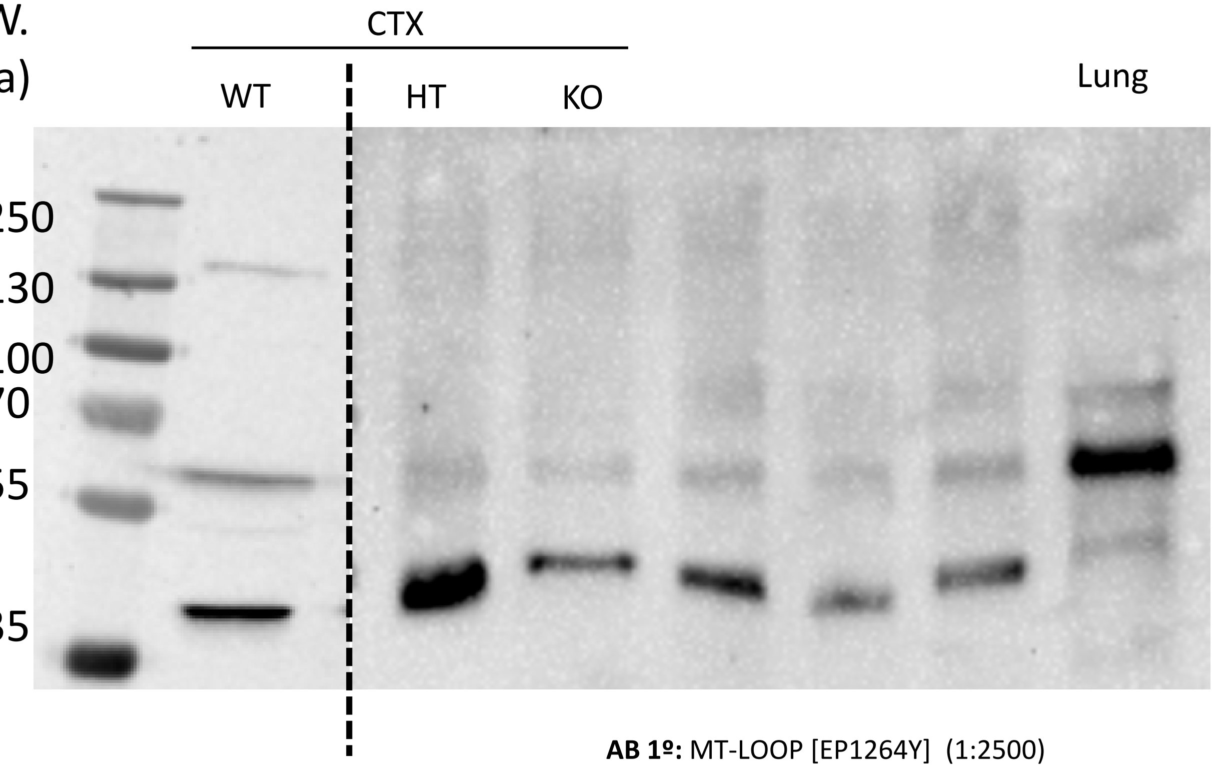

AB 1º: MT-LOOP [EP1264Y] (1:2500)
Ab 2º: goat anti- rabbit HRP (1:2500)

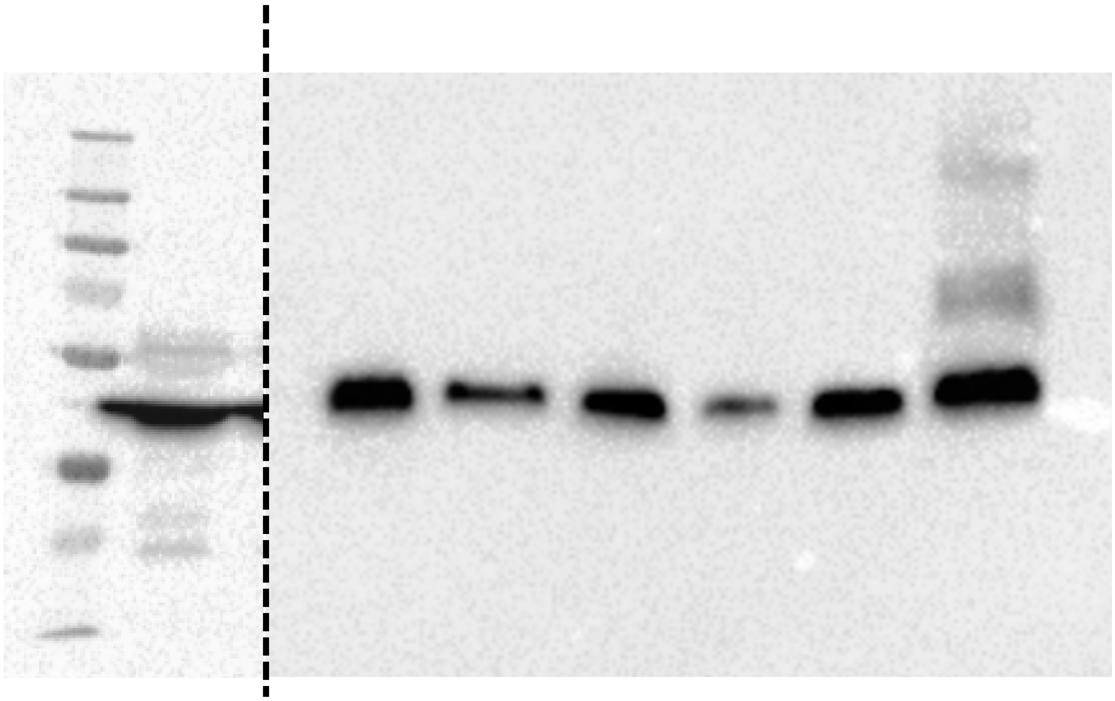

AB1º: monoclonal β-actin mouse (1:1000)
Ab 2º: goat anti-mouse HRP (1:2500)

Included in the composition

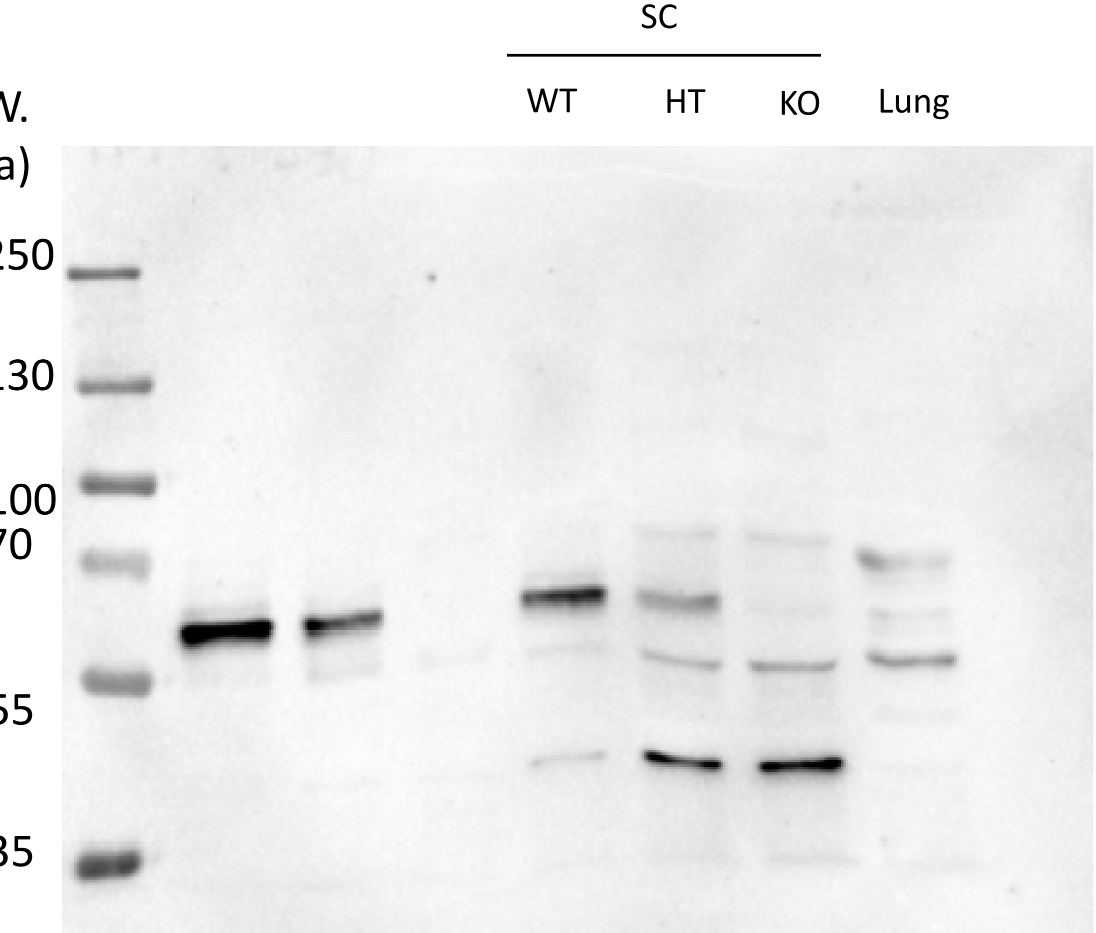

AB 1º: MT-LOOP [EP1264Y] (1:2500)
Ab 2º: goat anti- rabbit HRP (1:2500)

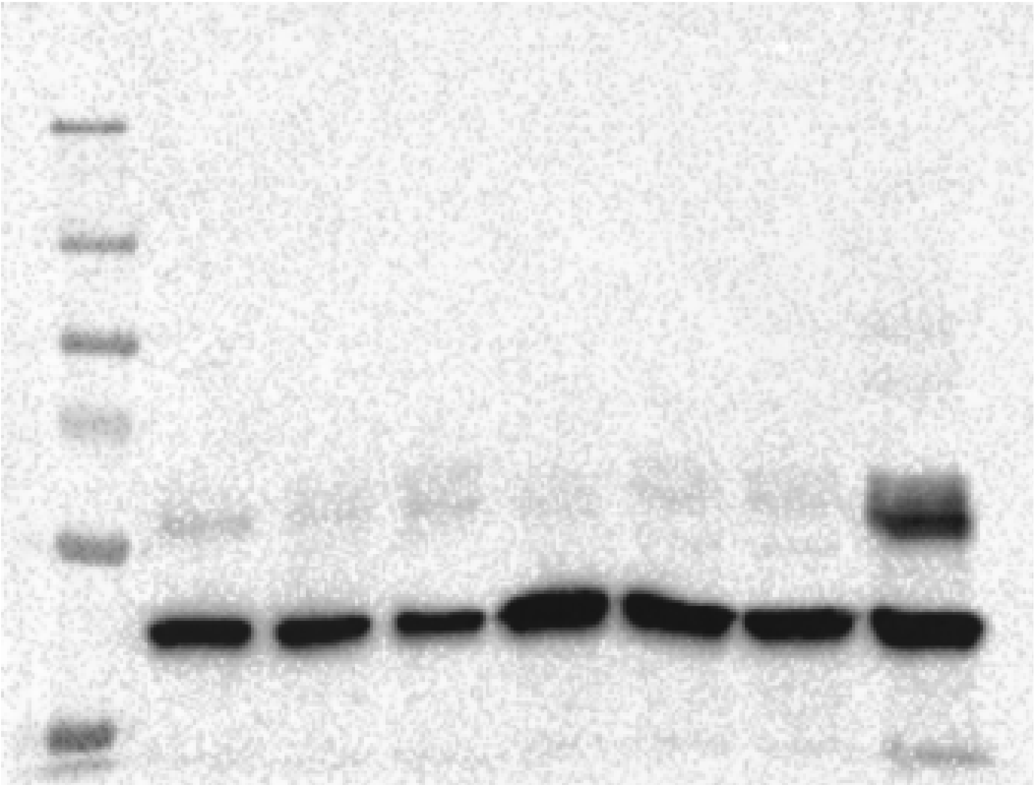

AB1º: monoclonal β-actin mouse (1:1000)
Ab 2º: goat anti-mouse HRP (1:2500)

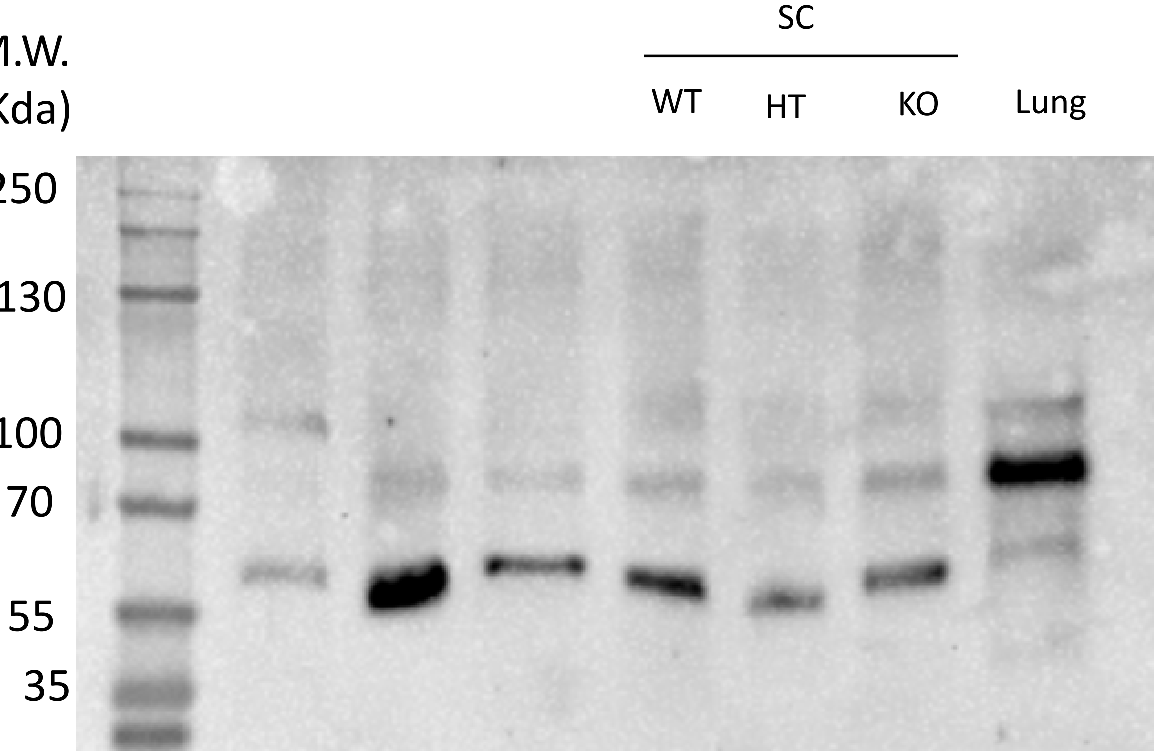

AB 1º: MT-LOOP [EP1264Y] (1:2500)
Ab 2º: goat anti- rabbit HRP (1:2500)

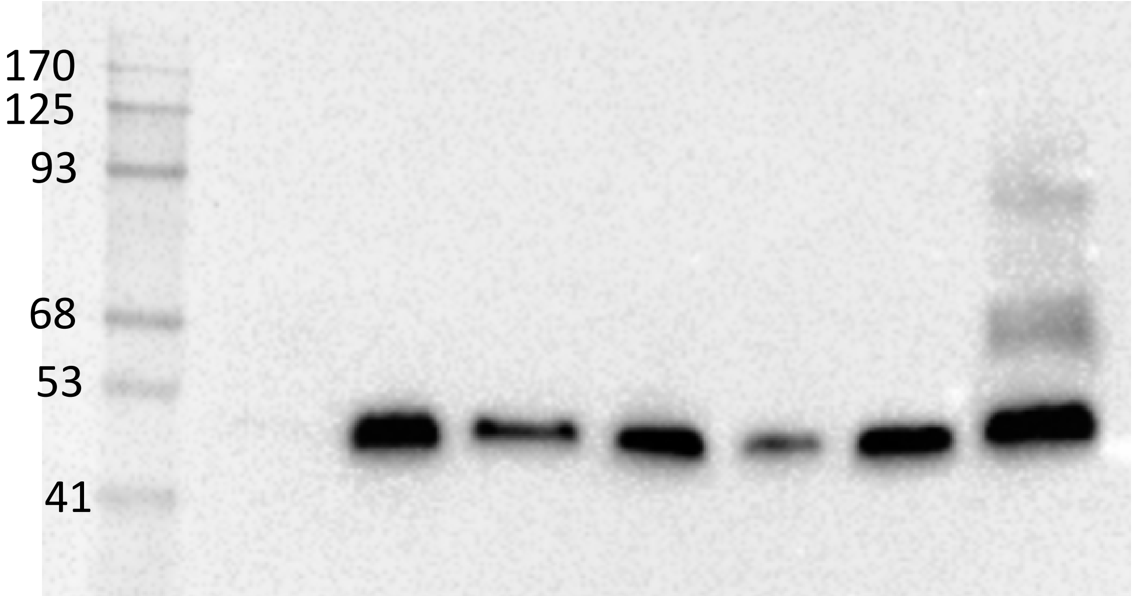

AB1º: monoclonal β-actin mouse (1:1000)
Ab 2º: goat anti-mouse HRP (1:2500)

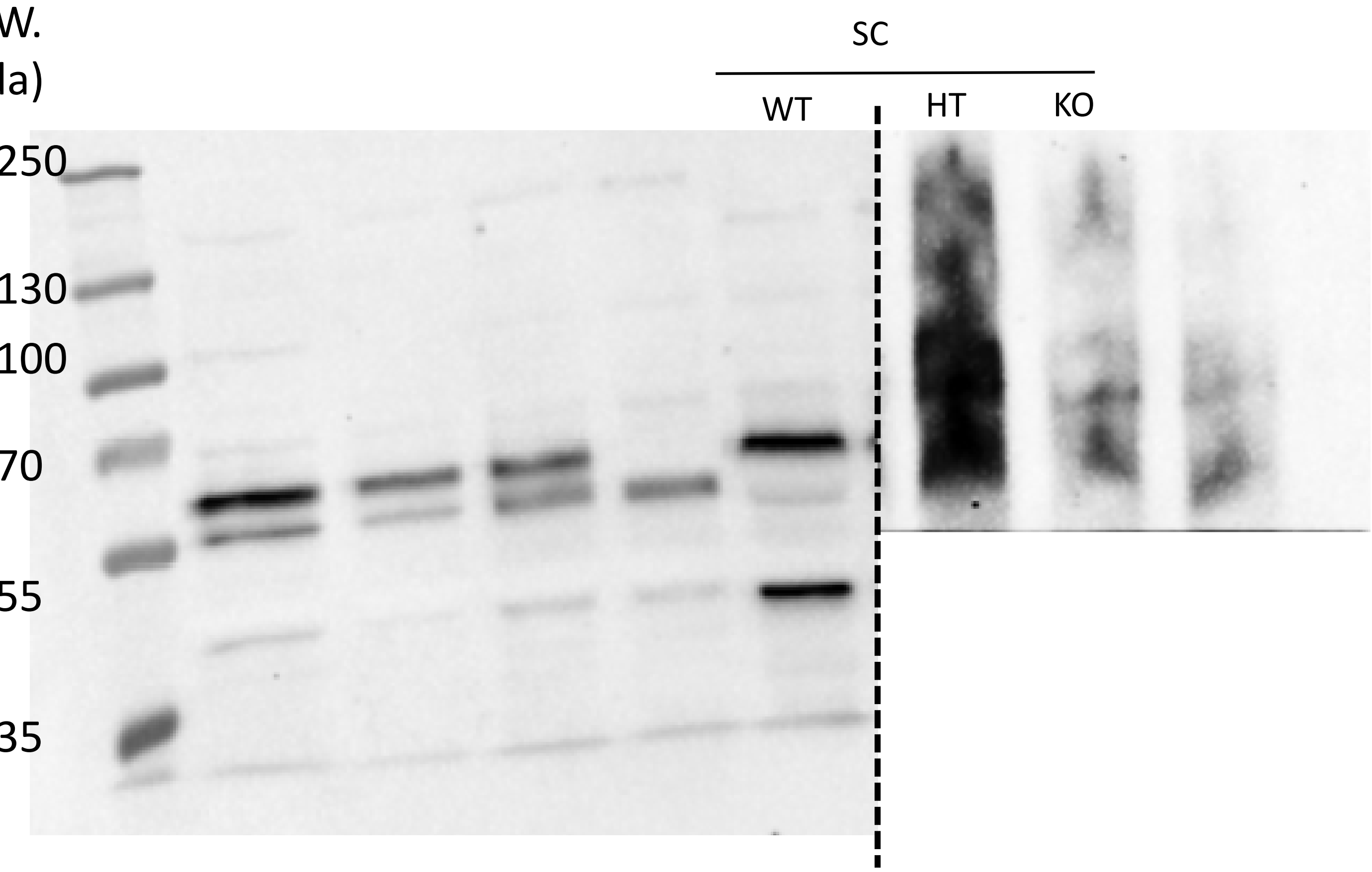

AB 1º: MT-LOOP [EP1264Y] (1:2500)
Ab 2º: goat anti- rabbit HRP (1:2500)

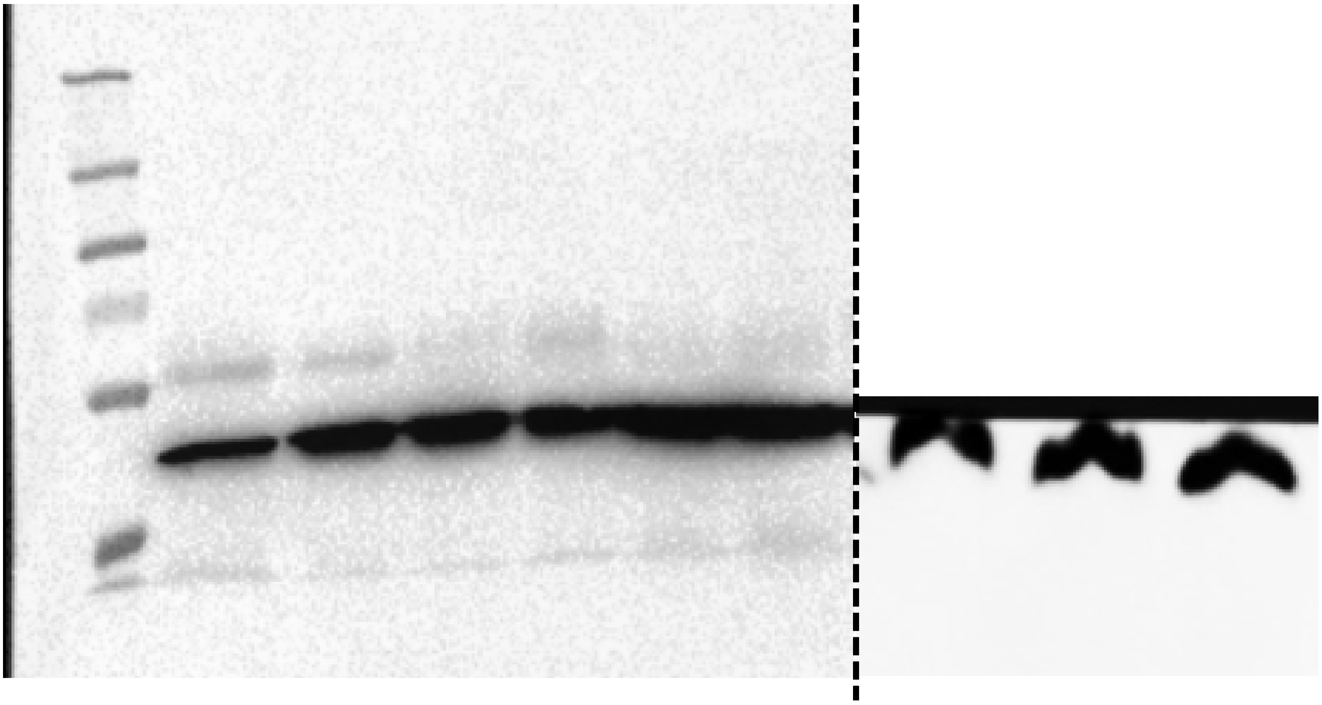

AB1º: monoclonal β-actin mouse (1:1000)
Ab 2º: goat anti-mouse HRP (1:2500)

Included in the composition

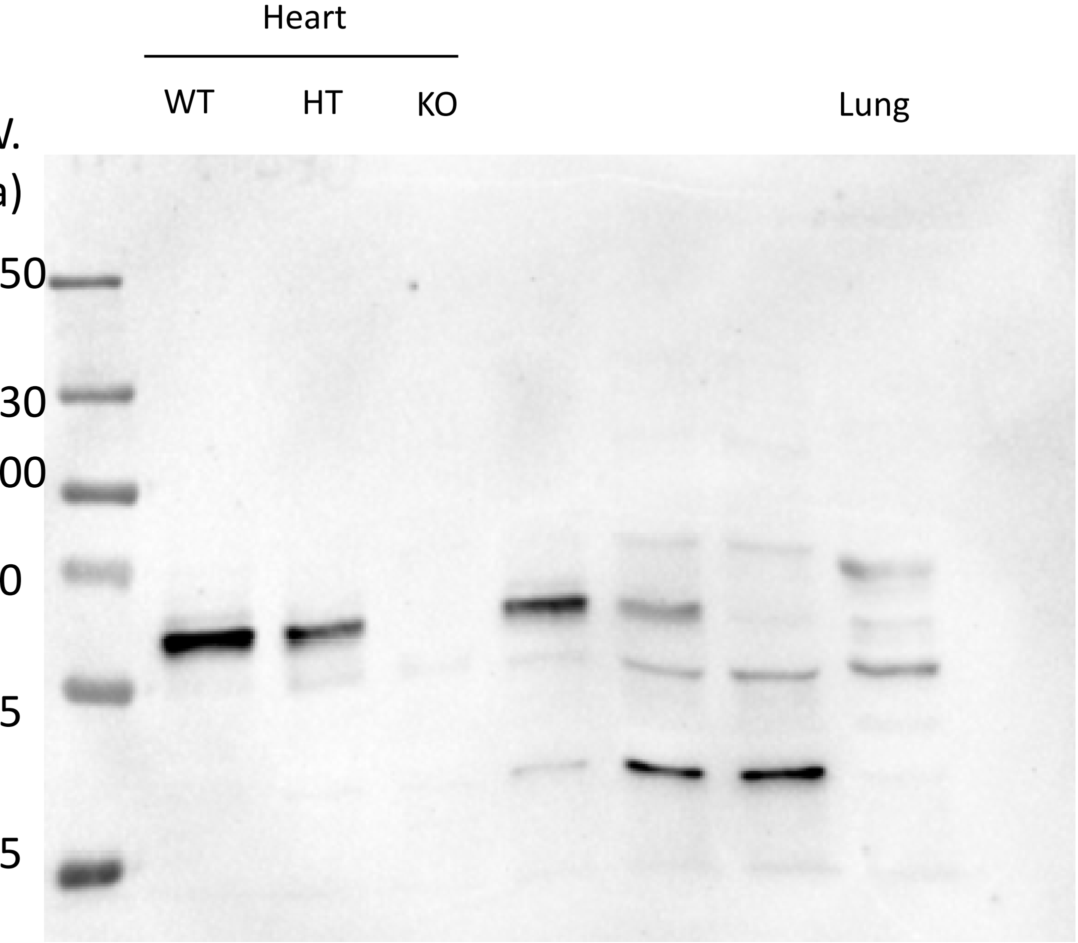

AB 1º: MT-LOOP [EP1264Y] (1:2500)
Ab 2º: goat anti- rabbit HRP (1:2500)

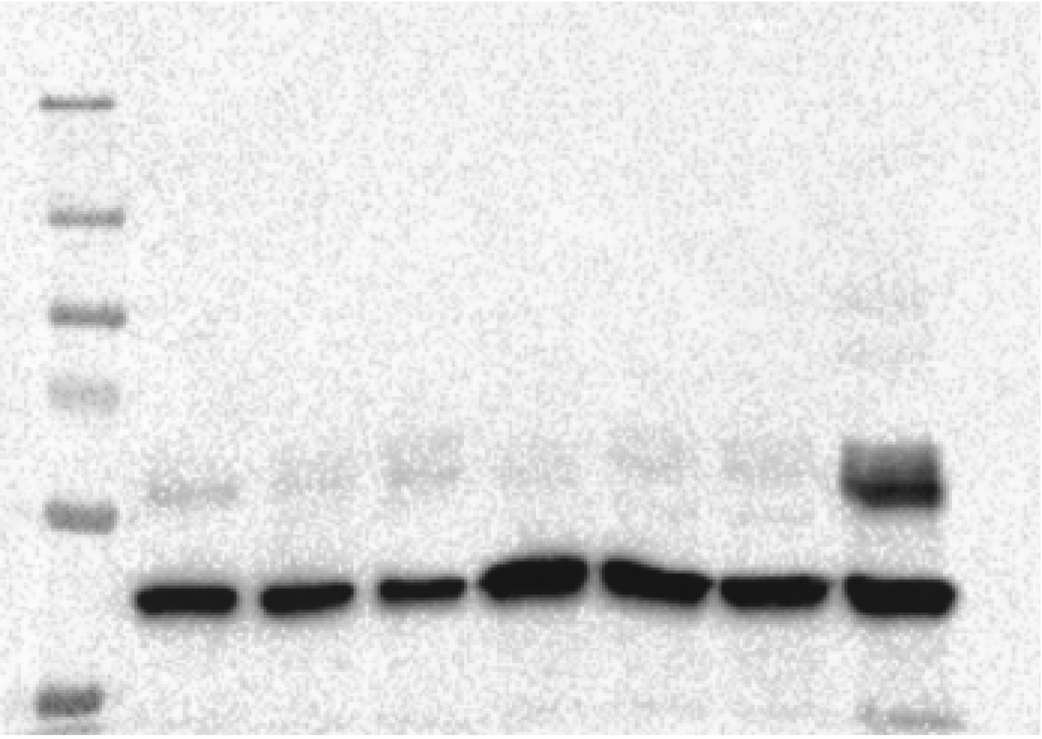

AB1º: monoclonal β-actin mouse (1:1000)
Ab 2º: goat anti-mouse HRP (1:2500)

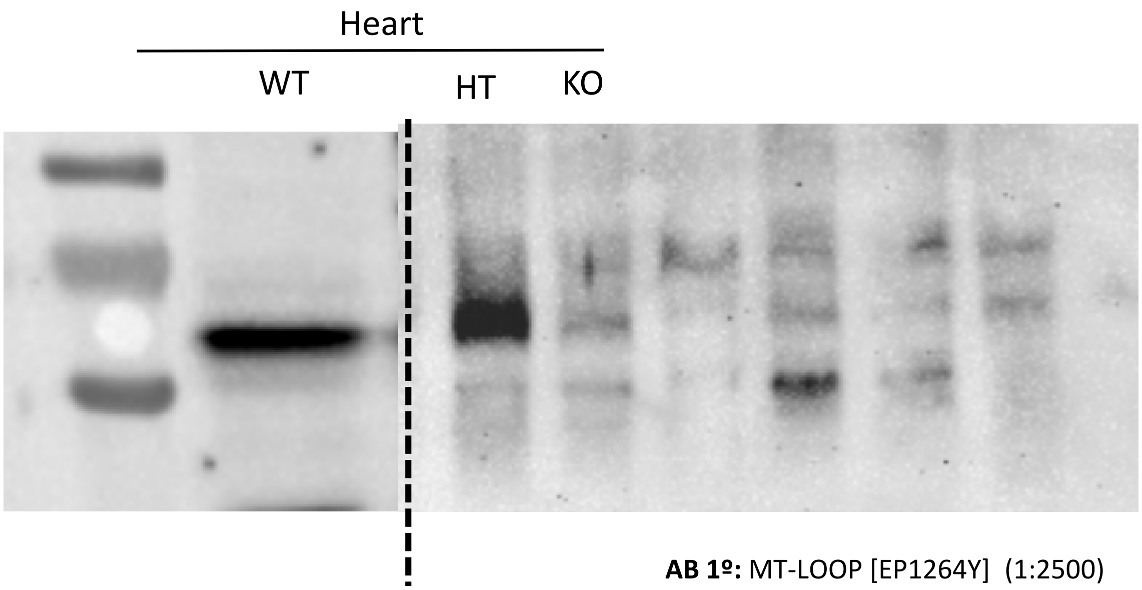

AB 1º: MT-LOOP [EP1264Y] (1:2500)
Ab 2º: goat anti- rabbit HRP (1:2500)

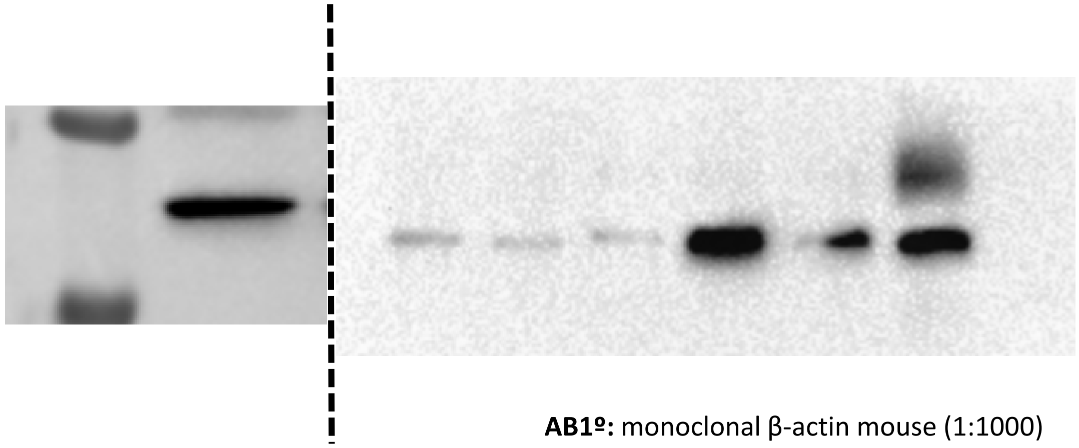

AB1º: monoclonal β-actin mouse (1:1000)
Ab 2º: goat anti-mouse HRP (1:2500)

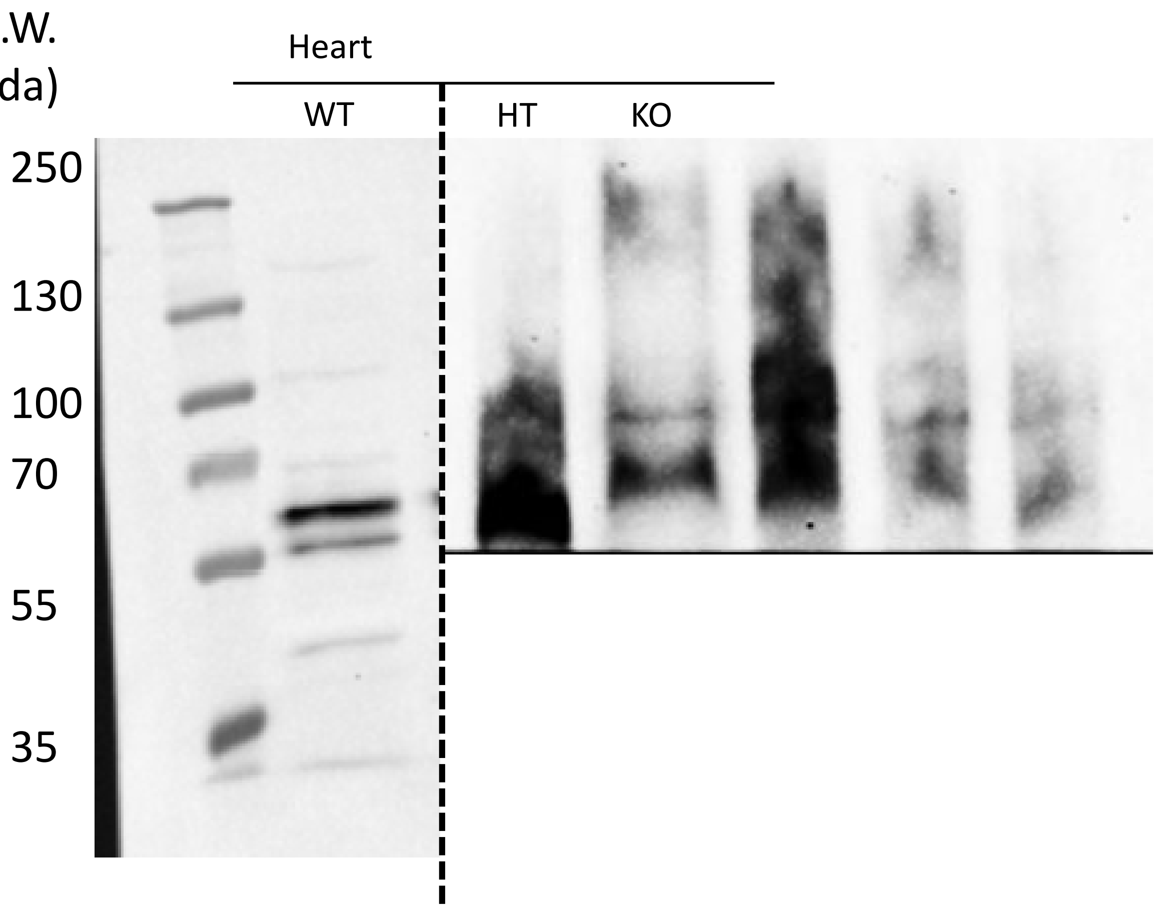

AB 1º: MT-LOOP [EP1264Y] (1:2500)
Ab 2º: goat anti- rabbit HRP (1:2500)

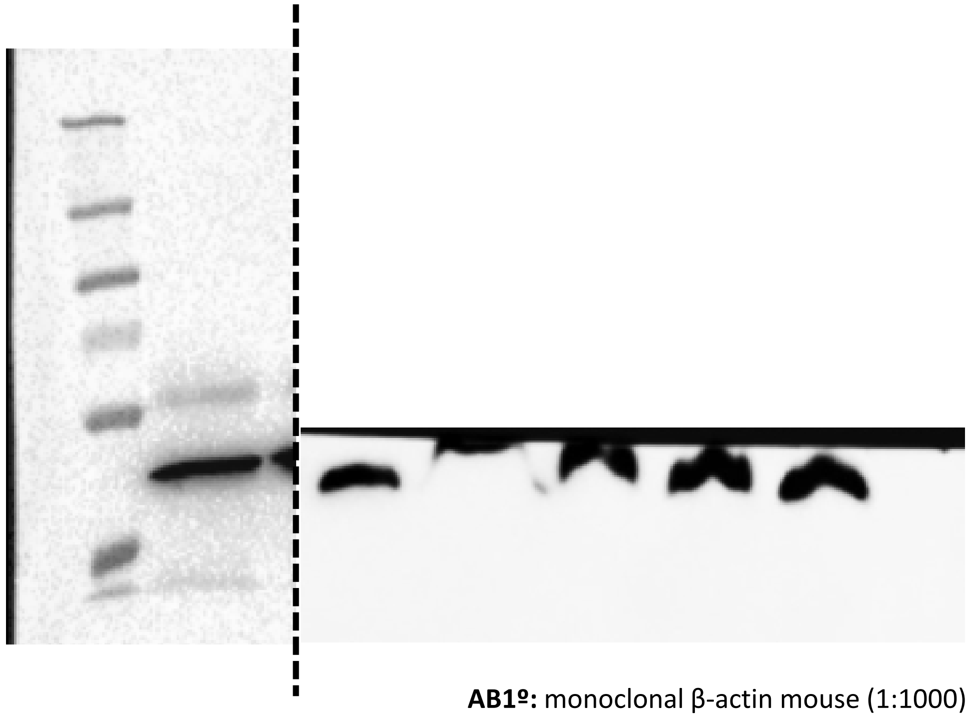

AB1º: monoclonal β-actin mouse (1:1000)
Ab 2º: goat anti-mouse HRP (1:2500)

Included in the composition

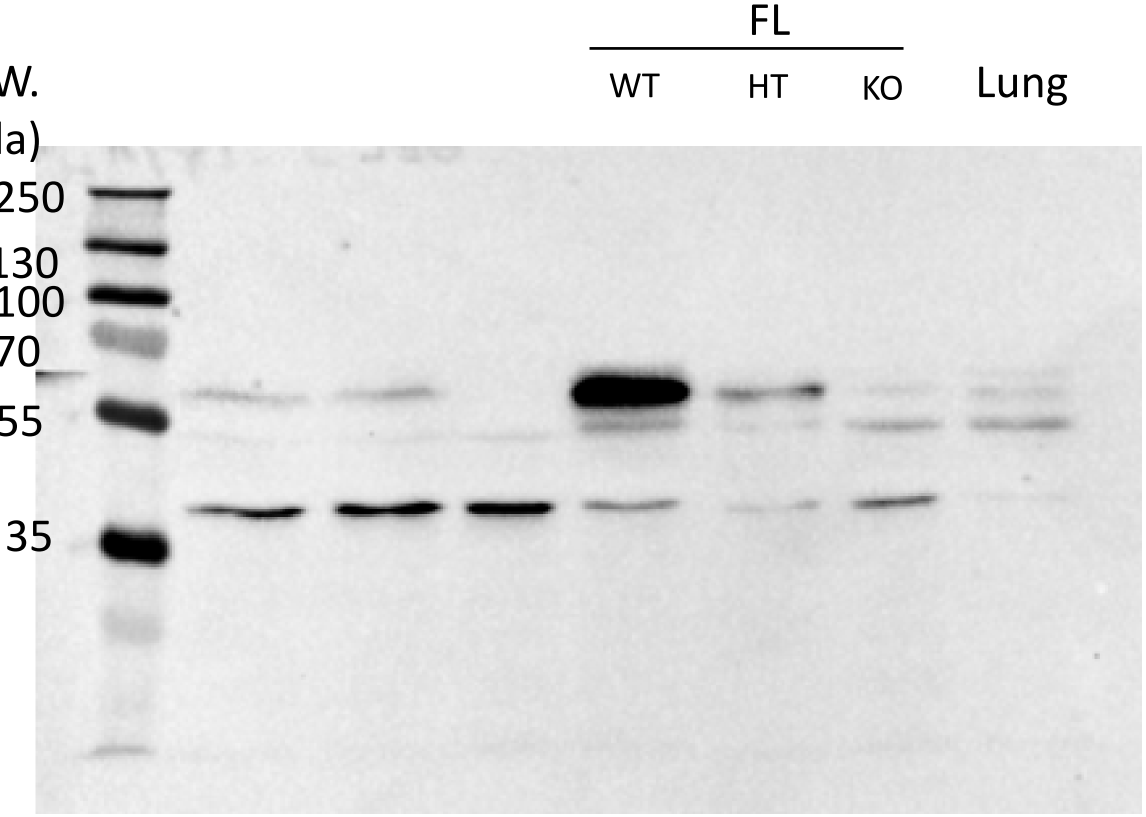

AB 1º: MT-LOOP [EP1264Y] (1:2500)
Ab 2º: goat anti- rabbit HRP (1:2500)

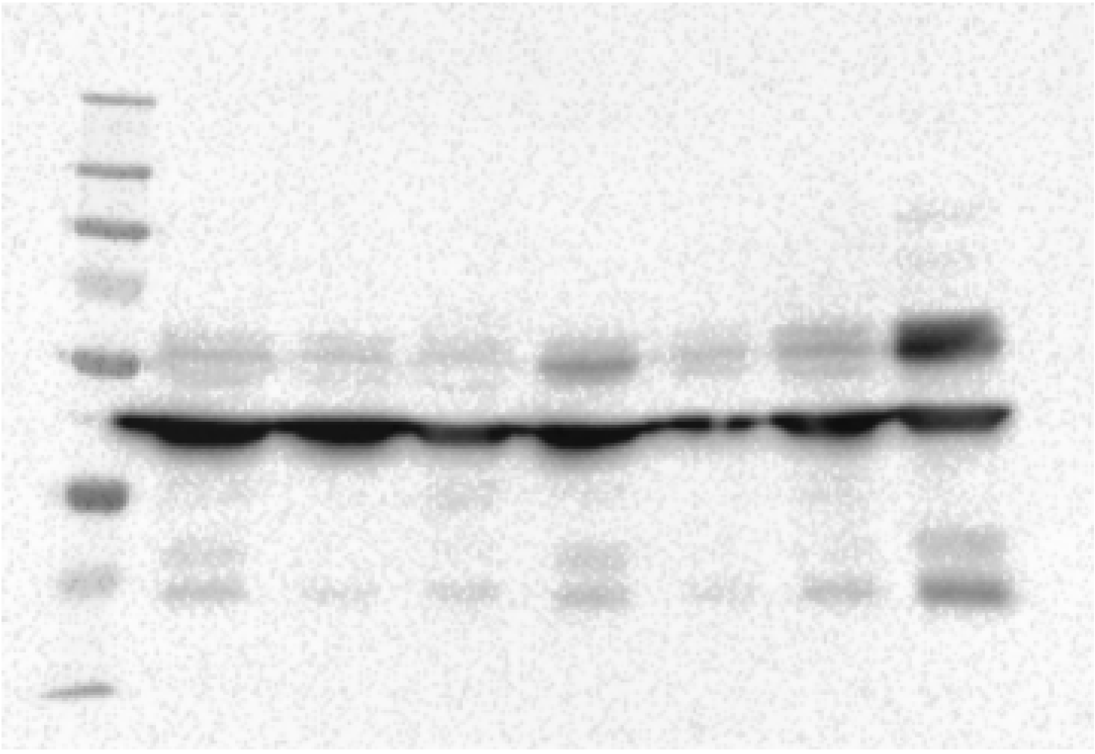

AB1º: monoclonal β-actin mouse (1:1000)
Ab 2º: goat anti-mouse HRP (1:2500)

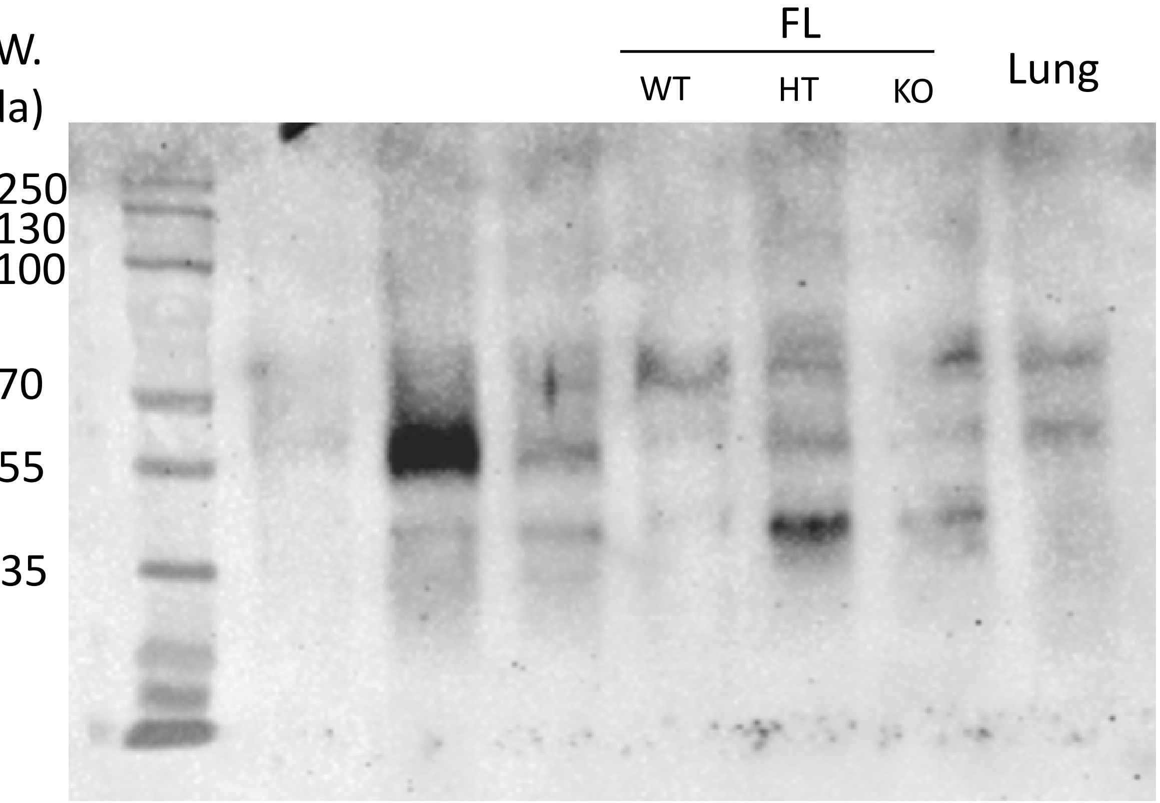

AB 1º: MT-LOOP [EP1264Y] (1:2500)
Ab 2º: goat anti- rabbit HRP (1:2500)

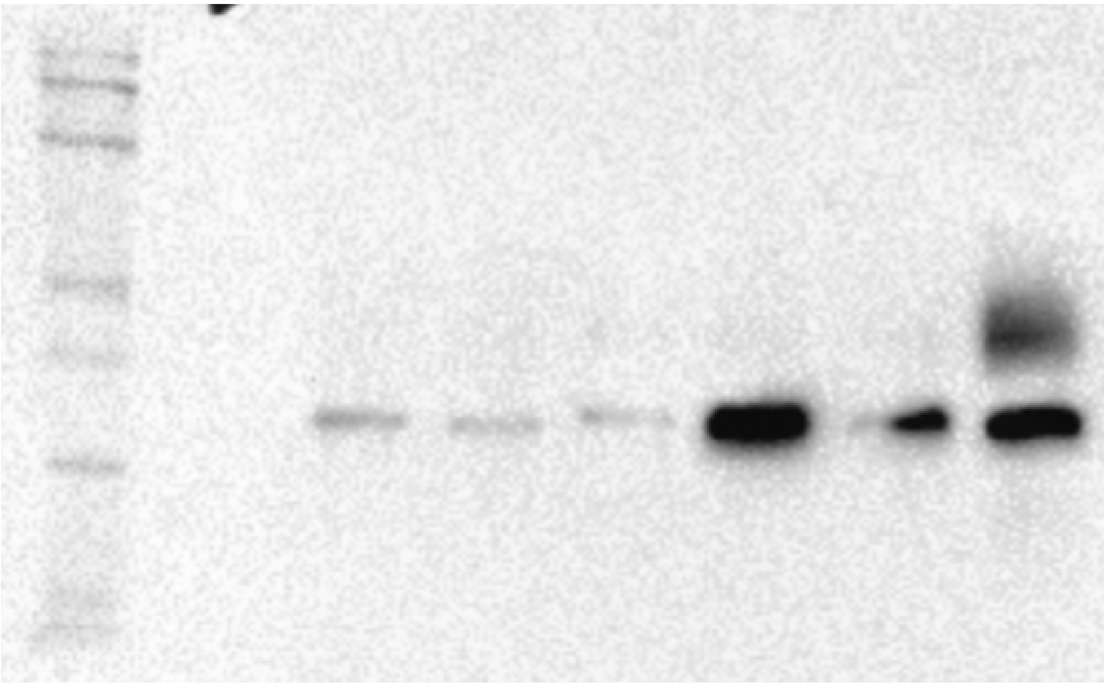

AB1º: monoclonal β-actin mouse (1:1000)
Ab 2º: goat anti-mouse HRP (1:2500)

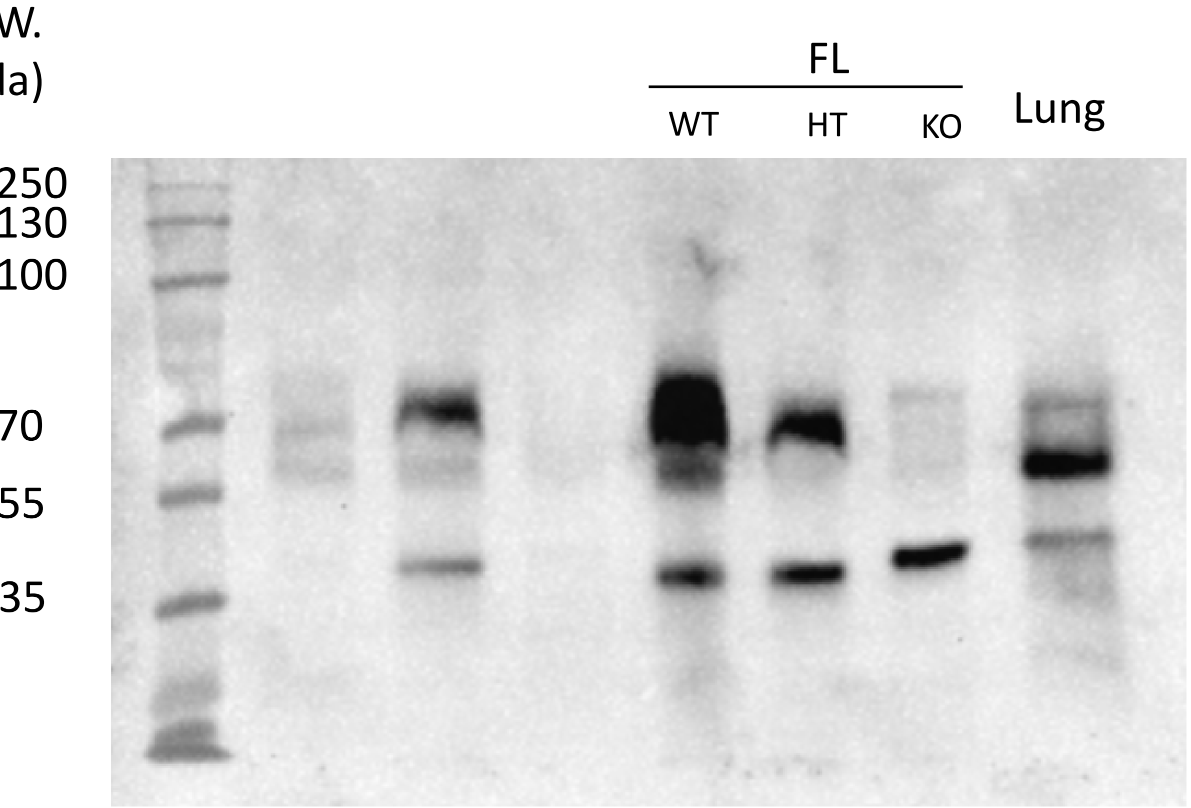

AB 1º: MT-LOOP [EP1264Y] (1:2500)
Ab 2º: goat anti- rabbit HRP (1:2500)

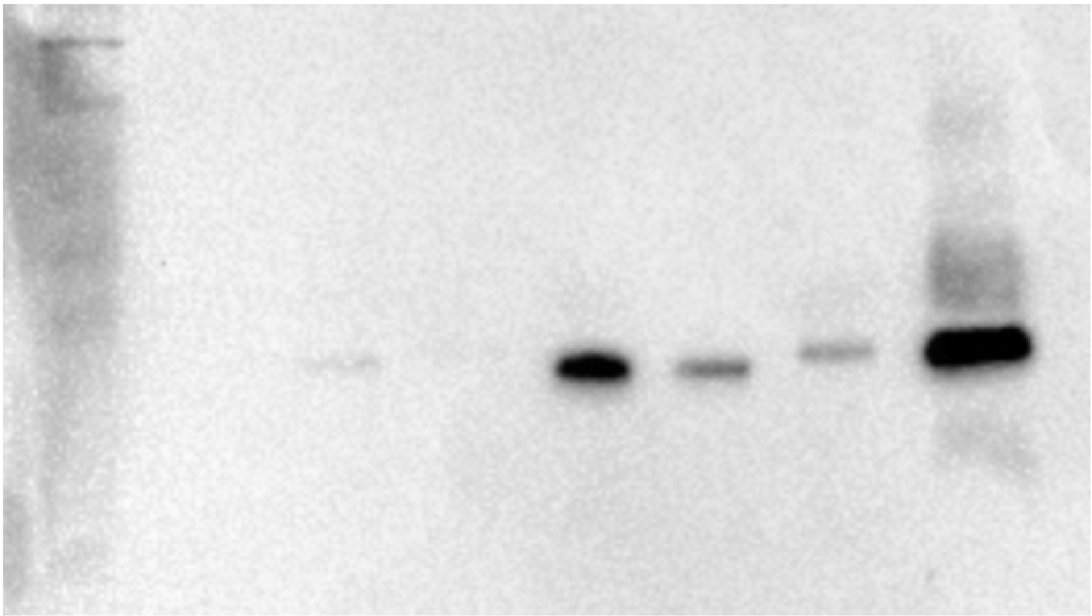

AB1º: monoclonal β-actin mouse (1:1000)
Ab 2º: goat anti-mouse HRP (1:2500)
