# Supplementary material for: Dynamic Expression of Membrane Type 1-Matrix Metalloproteinase (Mt1-mmp/Mmp14) in the Mouse Embryo
Source: Cells. 2021 Sep 17;10(9):2448. doi: 10.3390/cells10092448 (PMC8465375; doi:10.3390/cells10092448)
Supplement: Supplementary file 1 [file cells-10-02448-s001.zip › Sumplemmentary Material/Figure 3. Representative western-blot EMS 03 Sept 2021 EMS.pptx]

## Slide 1
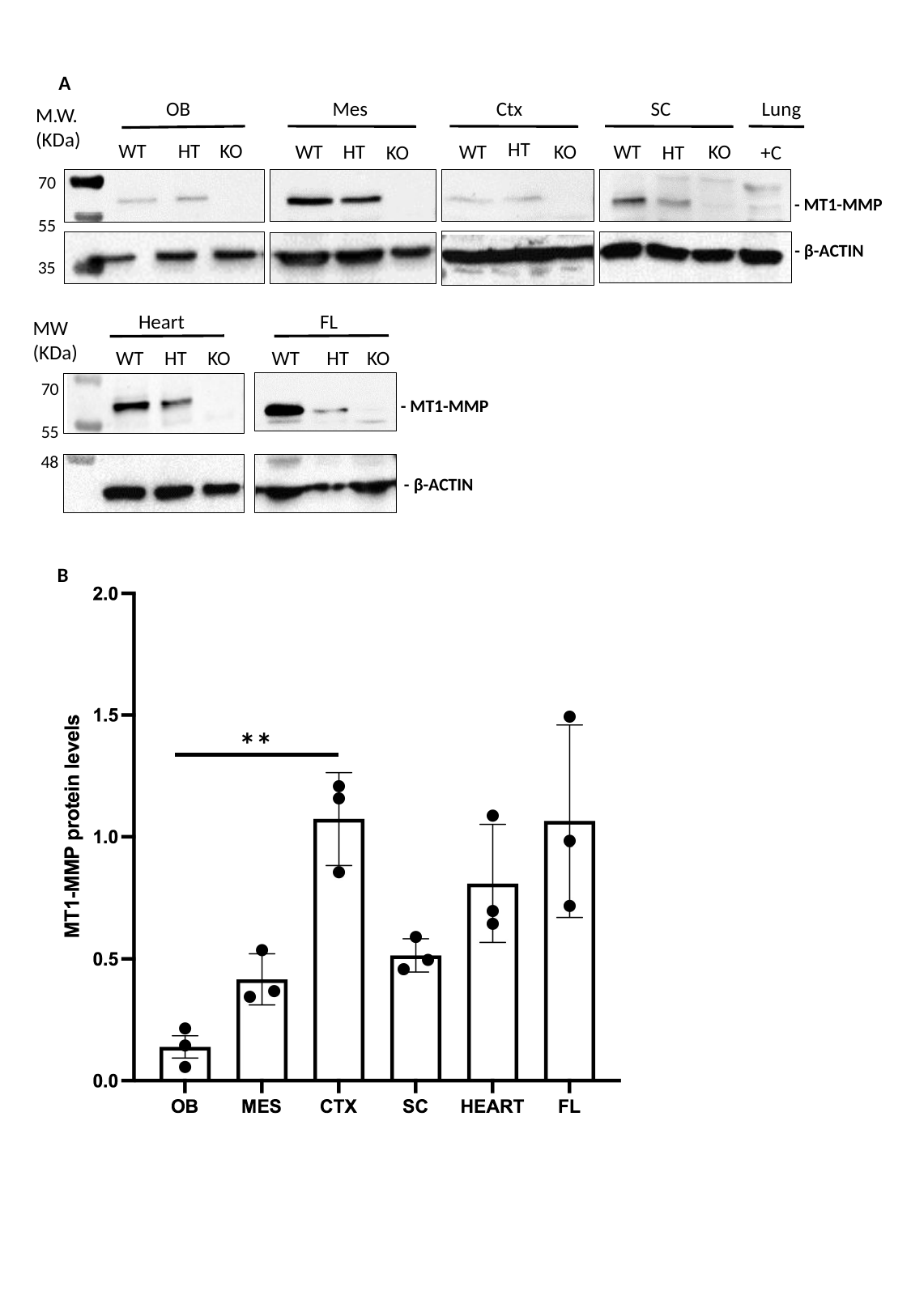

A
OB
Mes
Ctx
SC
Lung
M.W. (KDa)
HT
WT
HT
KO
KO
KO
WT
WT
WT
HT
+C
KO
HT
70
- MT1-MMP
55
- β-ACTIN
35
Heart
FL
MW (KDa)
WT
KO
HT
WT
HT
KO
70
- MT1-MMP
55
48
- β-ACTIN
B
**

## Slide 2
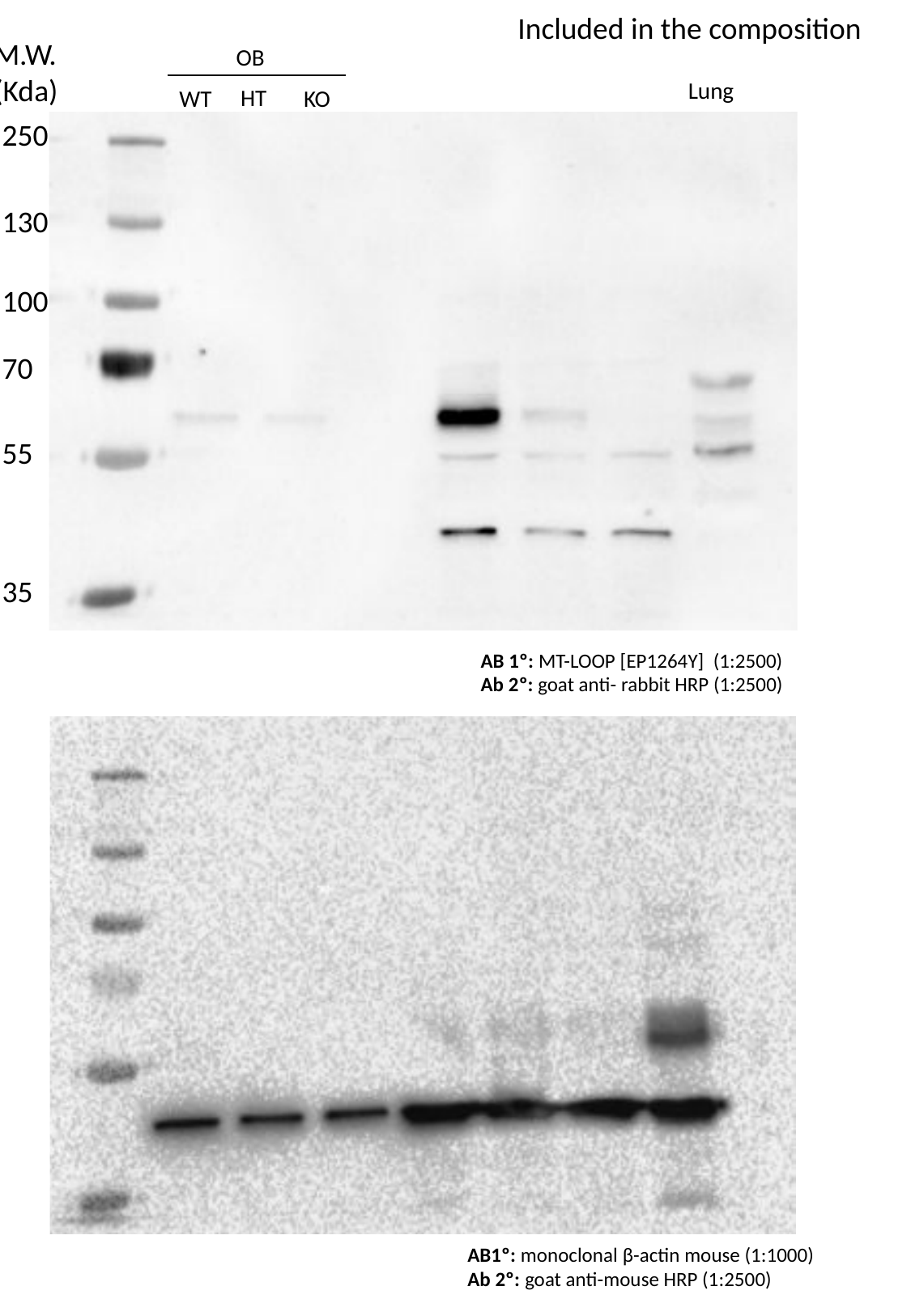

Included in the composition
M.W. (Kda)
OB
Lung
HT
WT
KO
250
130
100
70
55
35
AB 1º: MT-LOOP [EP1264Y] (1:2500)
Ab 2º: goat anti- rabbit HRP (1:2500)
AB1º: monoclonal β-actin mouse (1:1000)
Ab 2º: goat anti-mouse HRP (1:2500)

## Slide 3
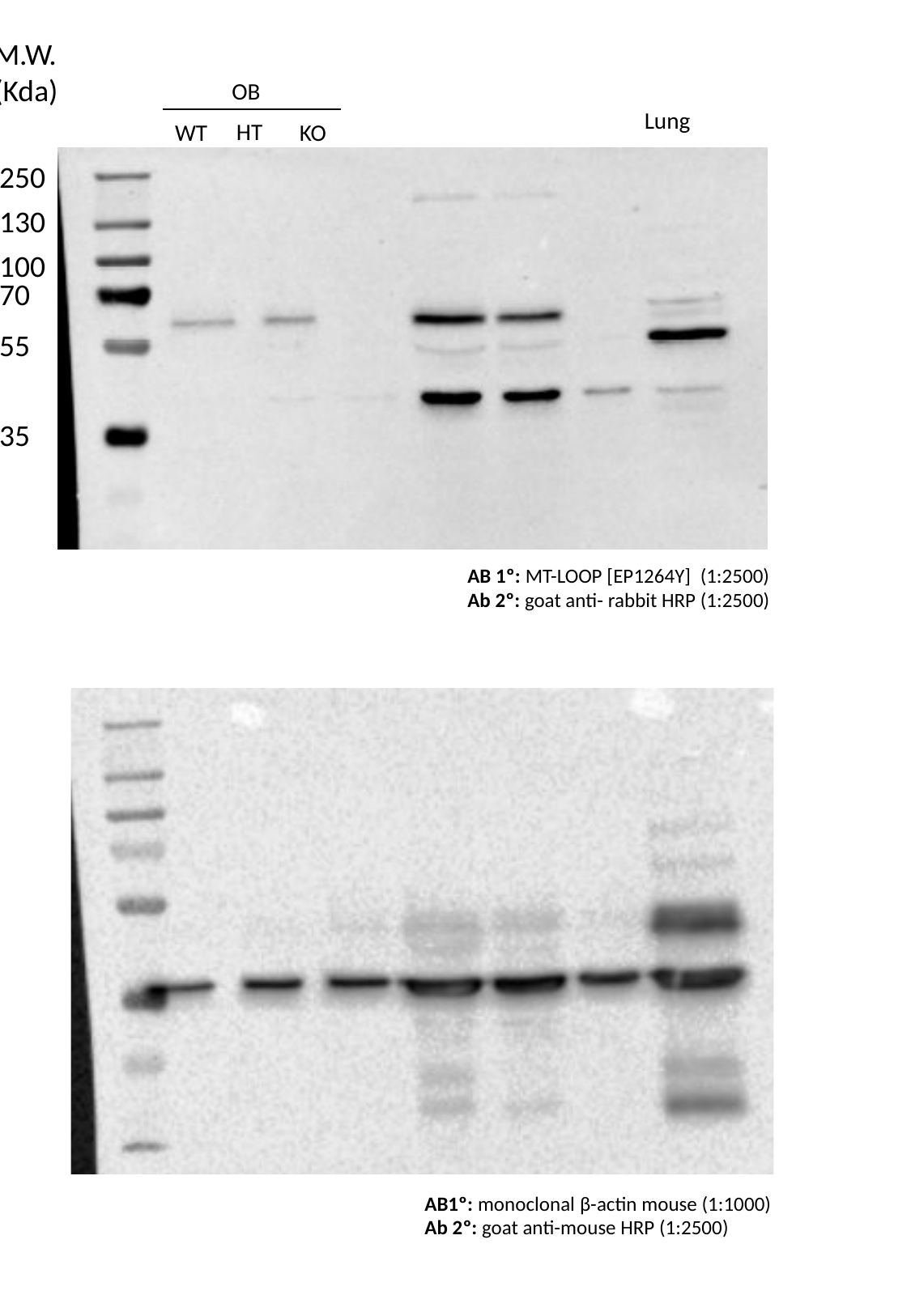

M.W. (Kda)
OB
Lung
HT
WT
KO
250
130
100
70
55
35
AB 1º: MT-LOOP [EP1264Y] (1:2500)
Ab 2º: goat anti- rabbit HRP (1:2500)
AB1º: monoclonal β-actin mouse (1:1000)
Ab 2º: goat anti-mouse HRP (1:2500)

## Slide 4
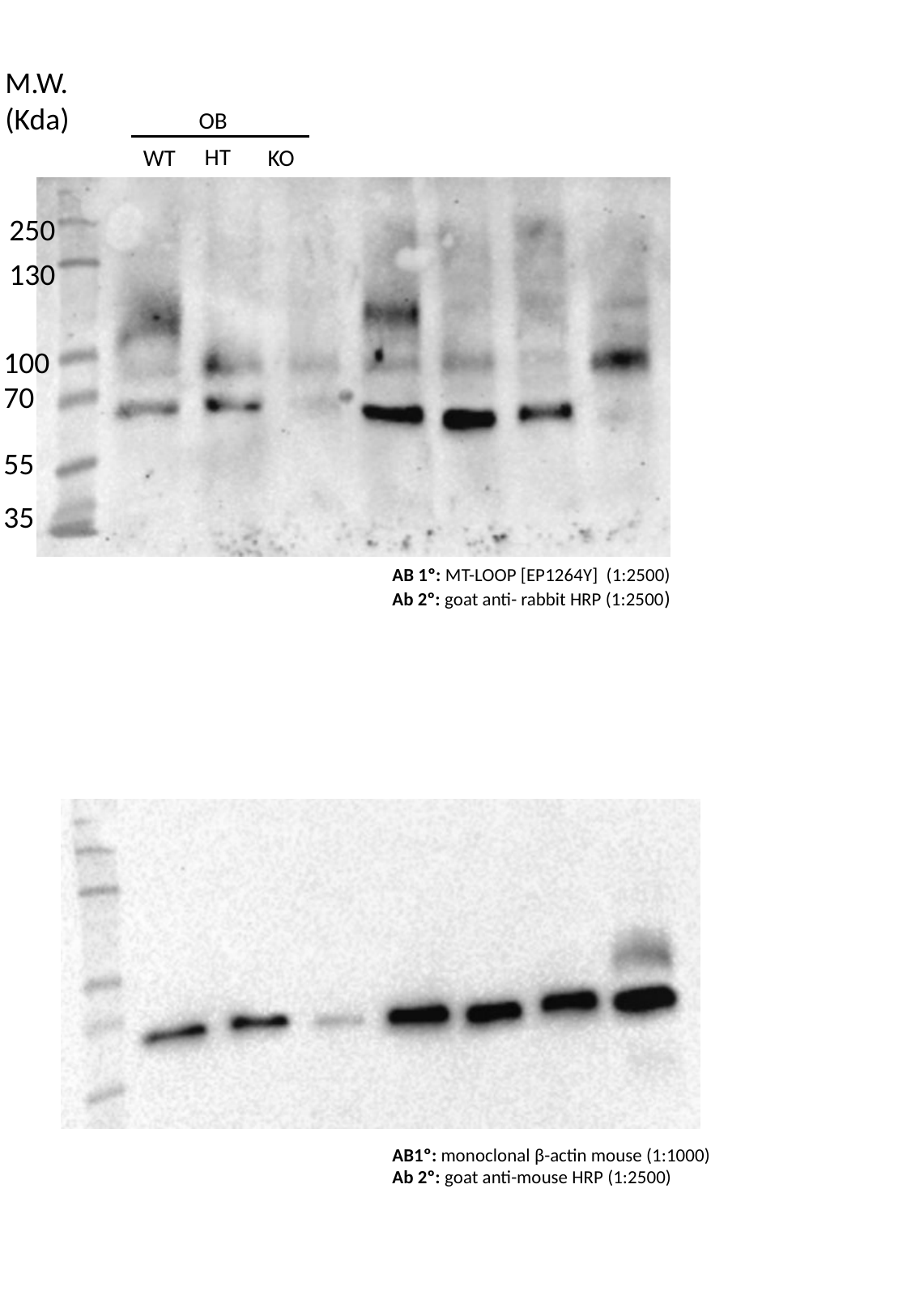

M.W. (Kda)
OB
HT
WT
KO
250
130
100
70
55
35
AB 1º: MT-LOOP [EP1264Y] (1:2500)
Ab 2º: goat anti- rabbit HRP (1:2500)
AB1º: monoclonal β-actin mouse (1:1000)
Ab 2º: goat anti-mouse HRP (1:2500)

## Slide 5
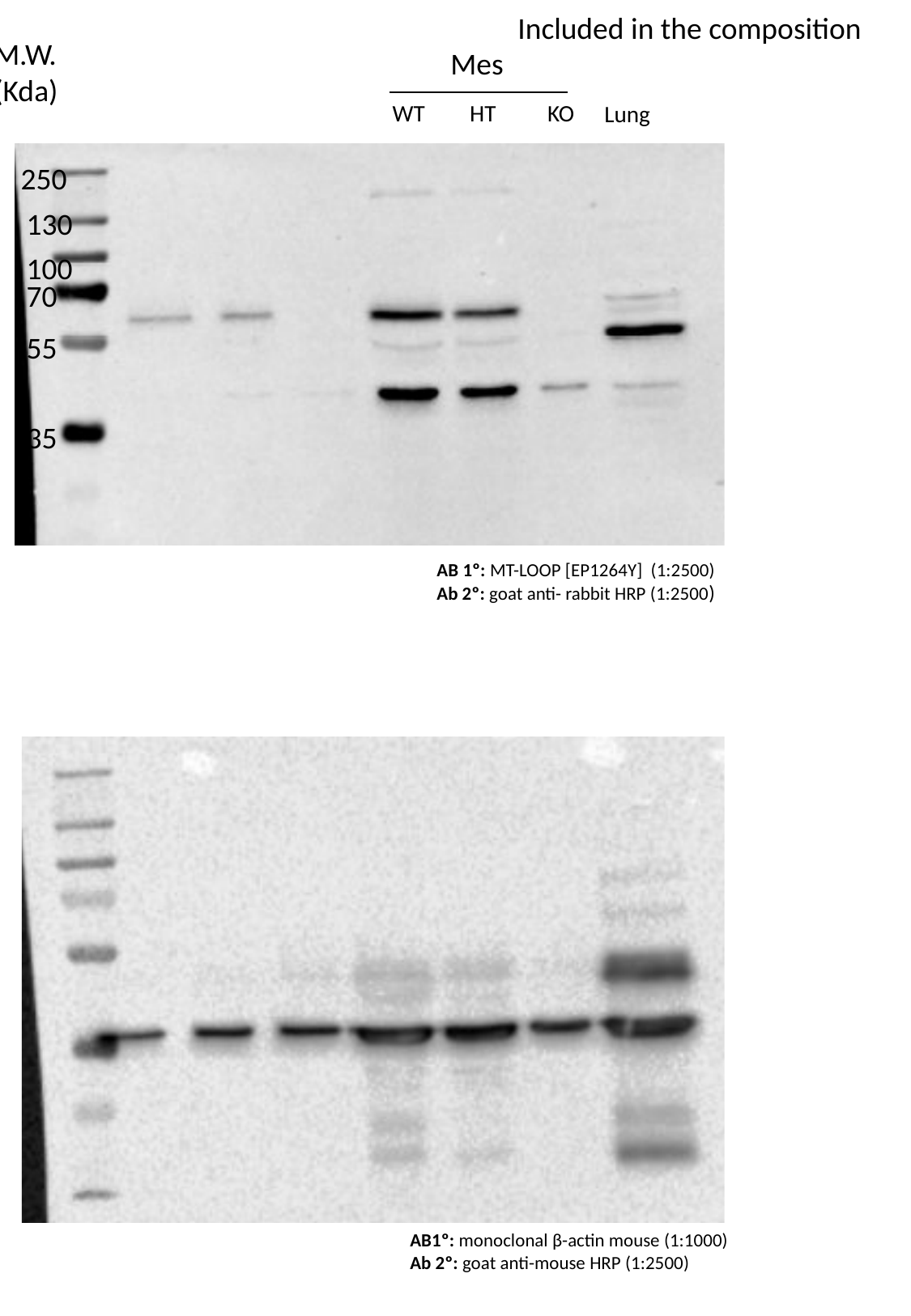

Included in the composition
M.W. (Kda)
Mes
WT
HT
KO
Lung
250
130
100
70
55
35
AB 1º: MT-LOOP [EP1264Y] (1:2500)
Ab 2º: goat anti- rabbit HRP (1:2500)
AB1º: monoclonal β-actin mouse (1:1000)
Ab 2º: goat anti-mouse HRP (1:2500)

## Slide 6
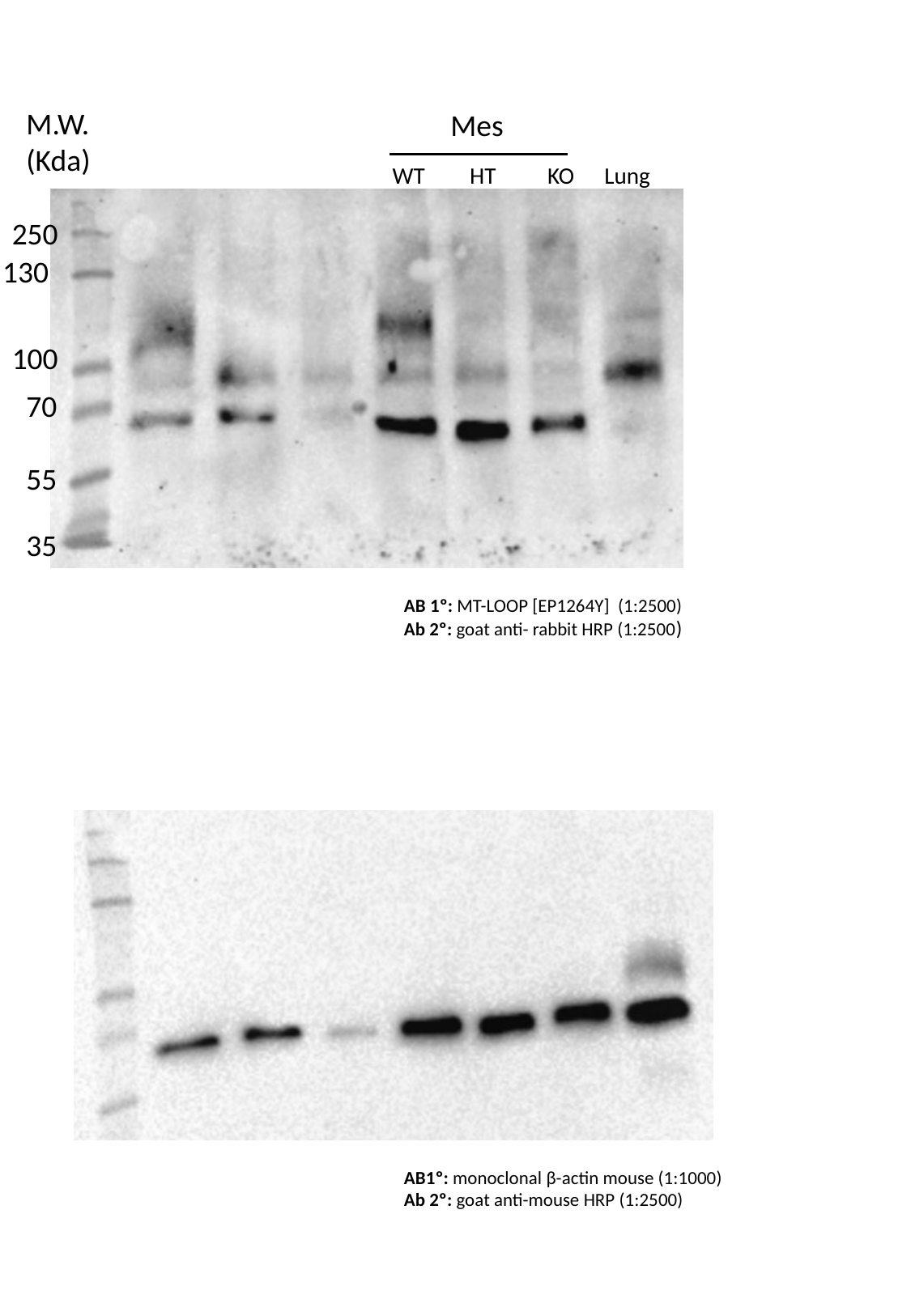

M.W. (Kda)
Mes
WT
HT
KO
Lung
250
130
100
70
55
35
AB 1º: MT-LOOP [EP1264Y] (1:2500)
Ab 2º: goat anti- rabbit HRP (1:2500)
AB1º: monoclonal β-actin mouse (1:1000)
Ab 2º: goat anti-mouse HRP (1:2500)

## Slide 7
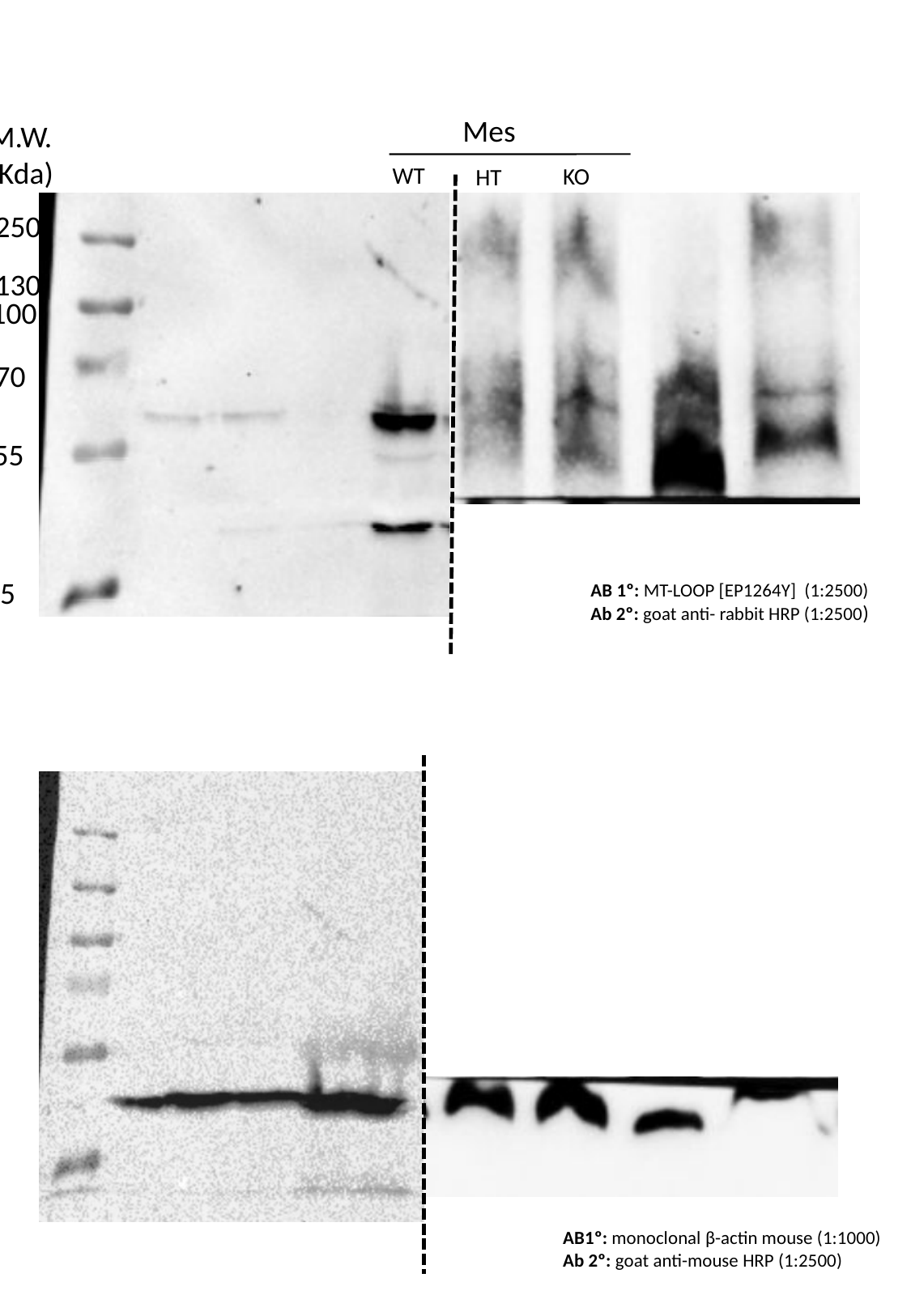

Mes
M.W. (Kda)
WT
KO
HT
250
130
100
70
55
35
AB 1º: MT-LOOP [EP1264Y] (1:2500)
Ab 2º: goat anti- rabbit HRP (1:2500)
AB1º: monoclonal β-actin mouse (1:1000)
Ab 2º: goat anti-mouse HRP (1:2500)

## Slide 8
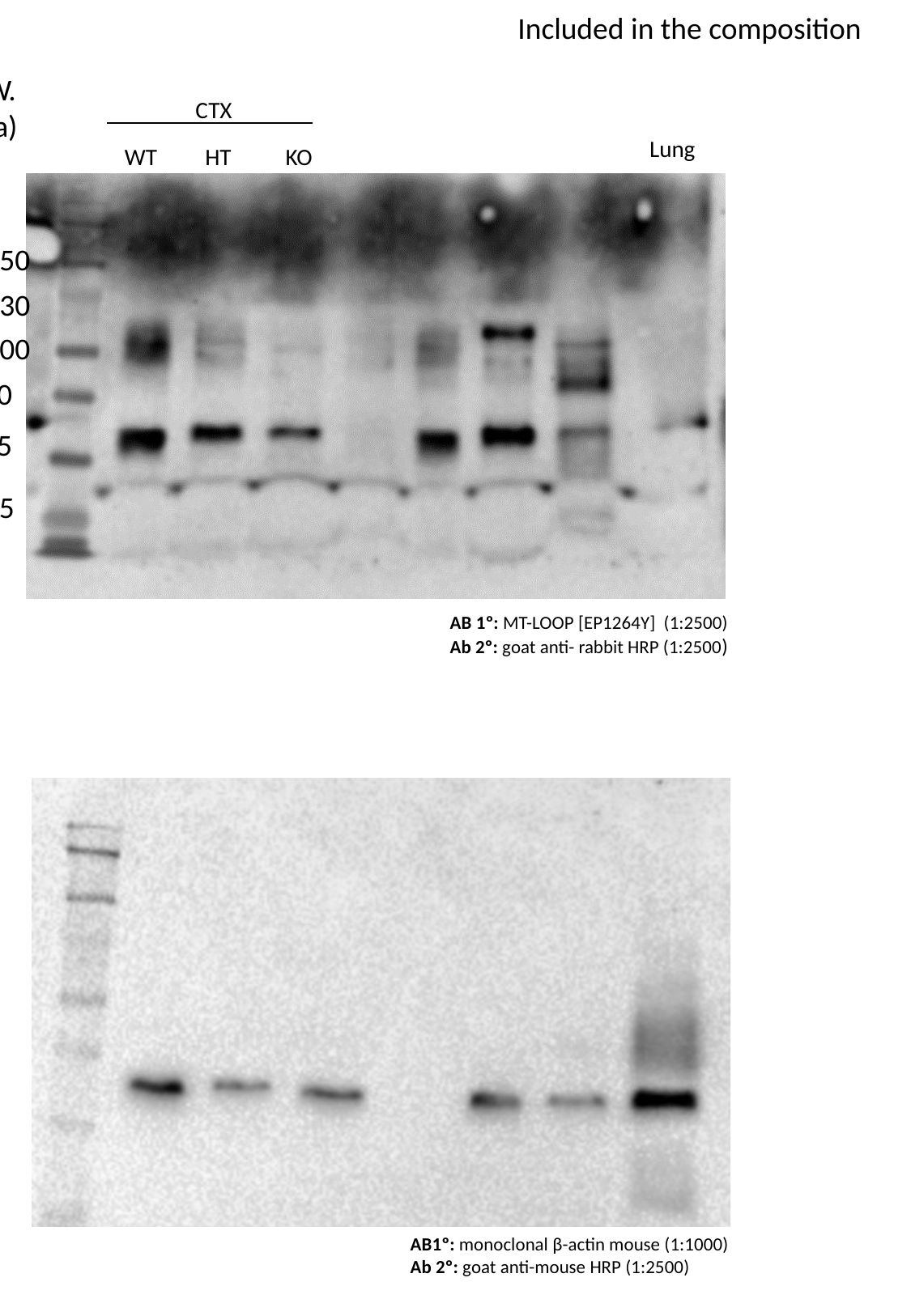

Included in the composition
M.W. (Kda)
CTX
WT
HT
KO
Lung
250
130
100
70
55
35
AB 1º: MT-LOOP [EP1264Y] (1:2500)
Ab 2º: goat anti- rabbit HRP (1:2500)
AB1º: monoclonal β-actin mouse (1:1000)
Ab 2º: goat anti-mouse HRP (1:2500)

## Slide 9
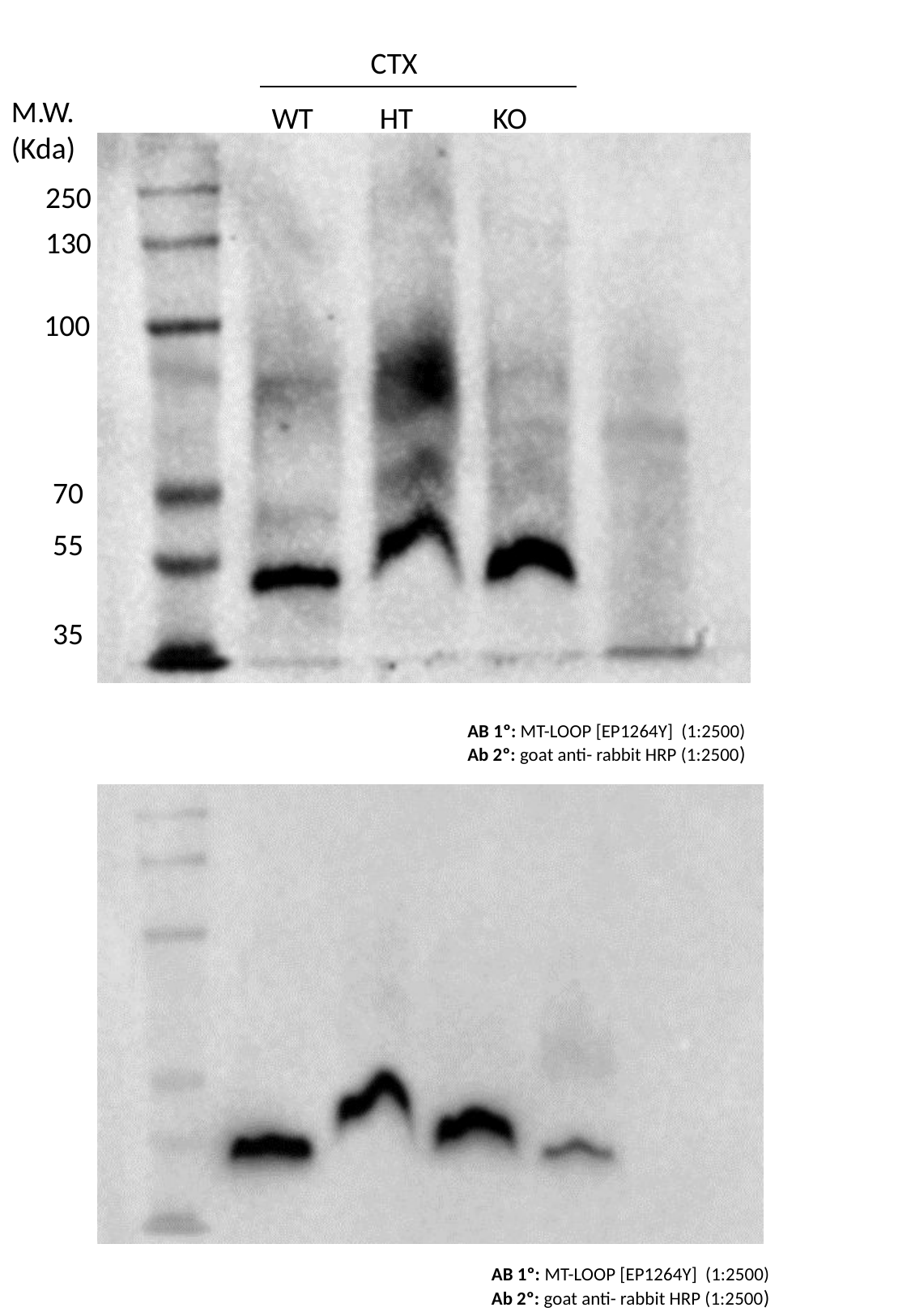

CTX
M.W. (Kda)
HT
KO
WT
250
130
100
70
55
35
AB 1º: MT-LOOP [EP1264Y] (1:2500)
Ab 2º: goat anti- rabbit HRP (1:2500)
AB 1º: MT-LOOP [EP1264Y] (1:2500)
Ab 2º: goat anti- rabbit HRP (1:2500)

## Slide 10
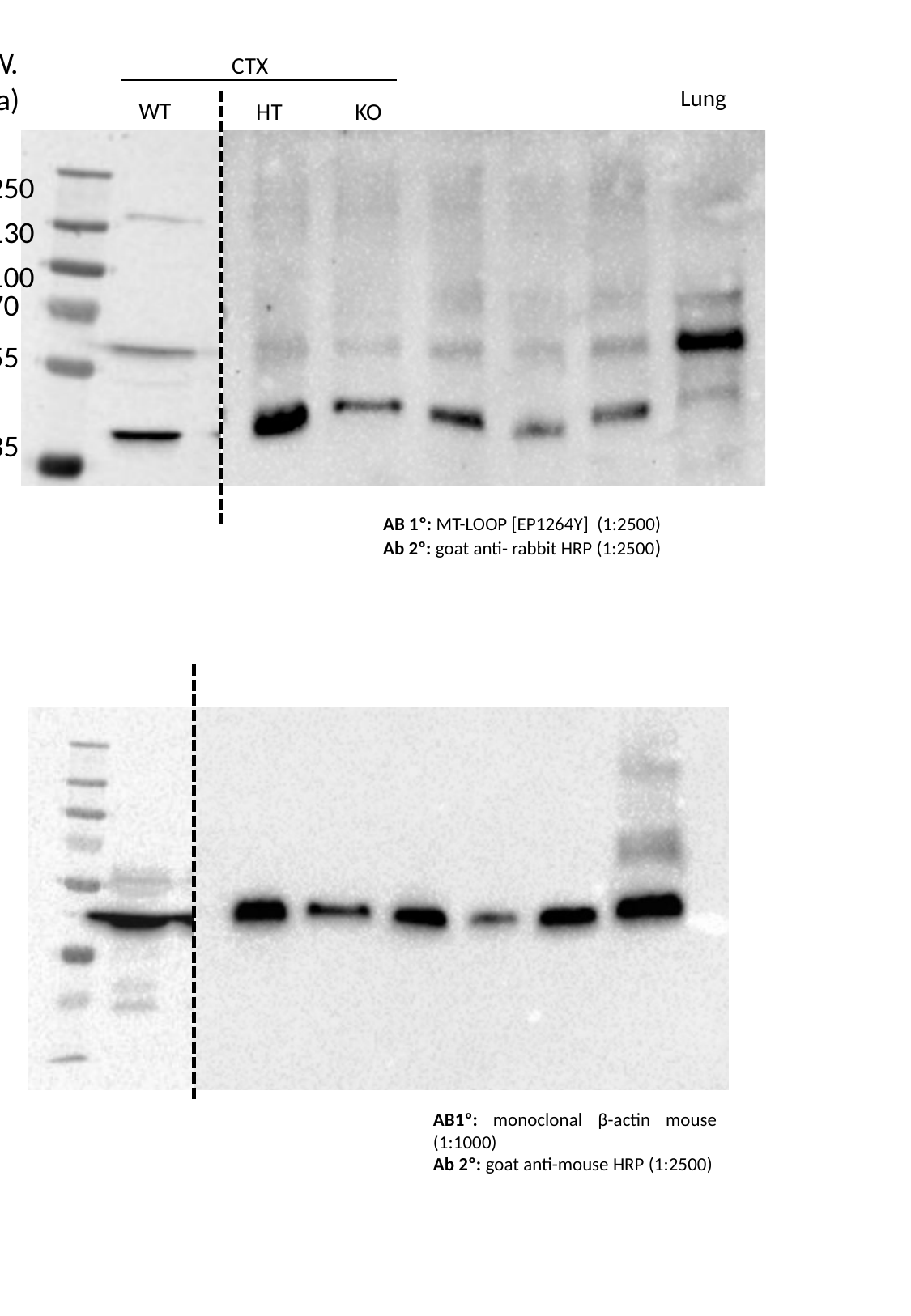

M.W. (Kda)
CTX
Lung
WT
HT
KO
250
130
100
70
55
35
AB 1º: MT-LOOP [EP1264Y] (1:2500)
Ab 2º: goat anti- rabbit HRP (1:2500)
AB1º: monoclonal β-actin mouse (1:1000)
Ab 2º: goat anti-mouse HRP (1:2500)

## Slide 11
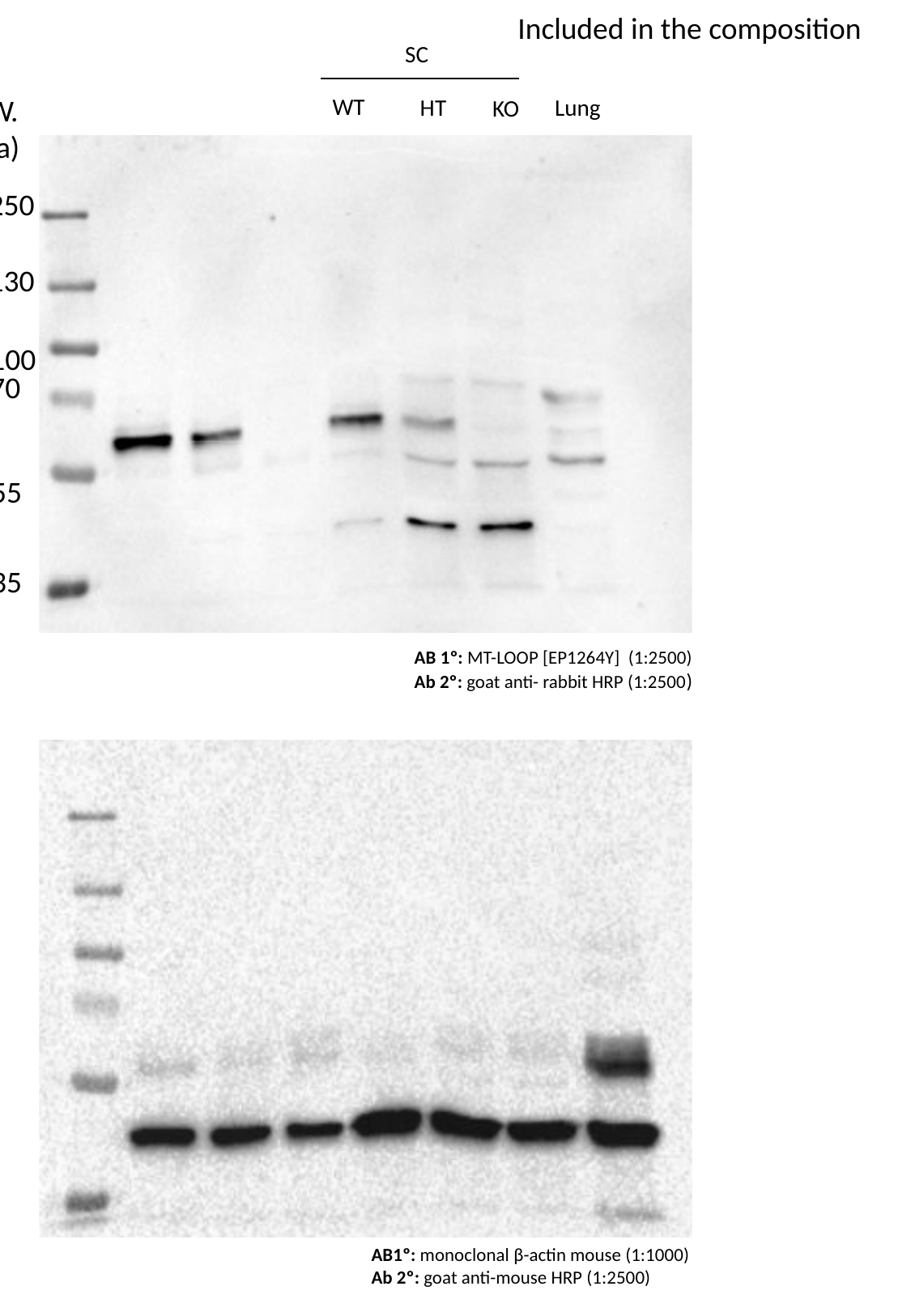

Included in the composition
SC
M.W. (Kda)
WT
Lung
HT
KO
250
130
100
70
55
35
AB 1º: MT-LOOP [EP1264Y] (1:2500)
Ab 2º: goat anti- rabbit HRP (1:2500)
AB1º: monoclonal β-actin mouse (1:1000)
Ab 2º: goat anti-mouse HRP (1:2500)

## Slide 12
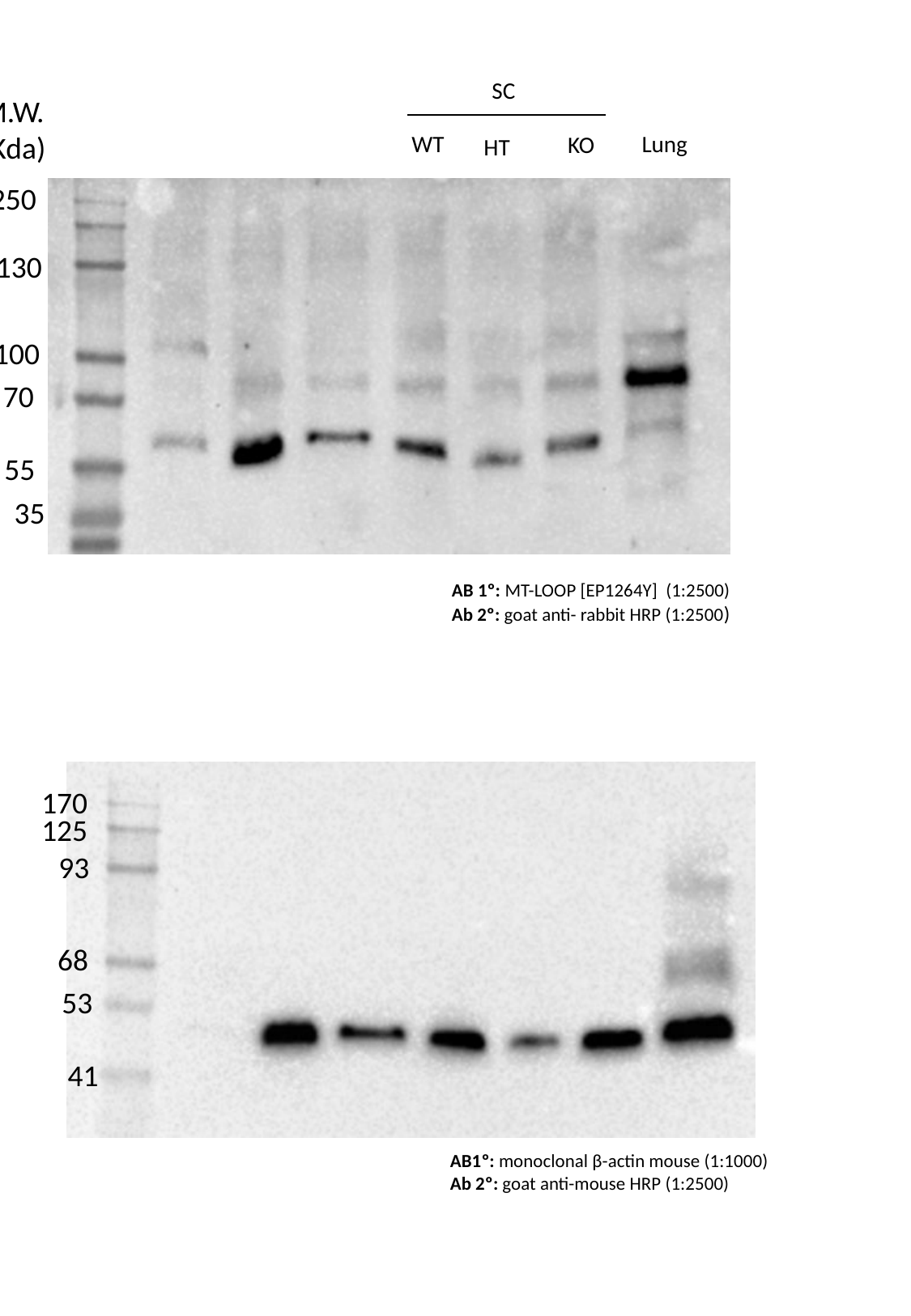

SC
M.W. (Kda)
WT
Lung
KO
HT
250
130
100
70
55
35
AB 1º: MT-LOOP [EP1264Y] (1:2500)
Ab 2º: goat anti- rabbit HRP (1:2500)
170
125
93
68
53
41
AB1º: monoclonal β-actin mouse (1:1000)
Ab 2º: goat anti-mouse HRP (1:2500)

## Slide 13
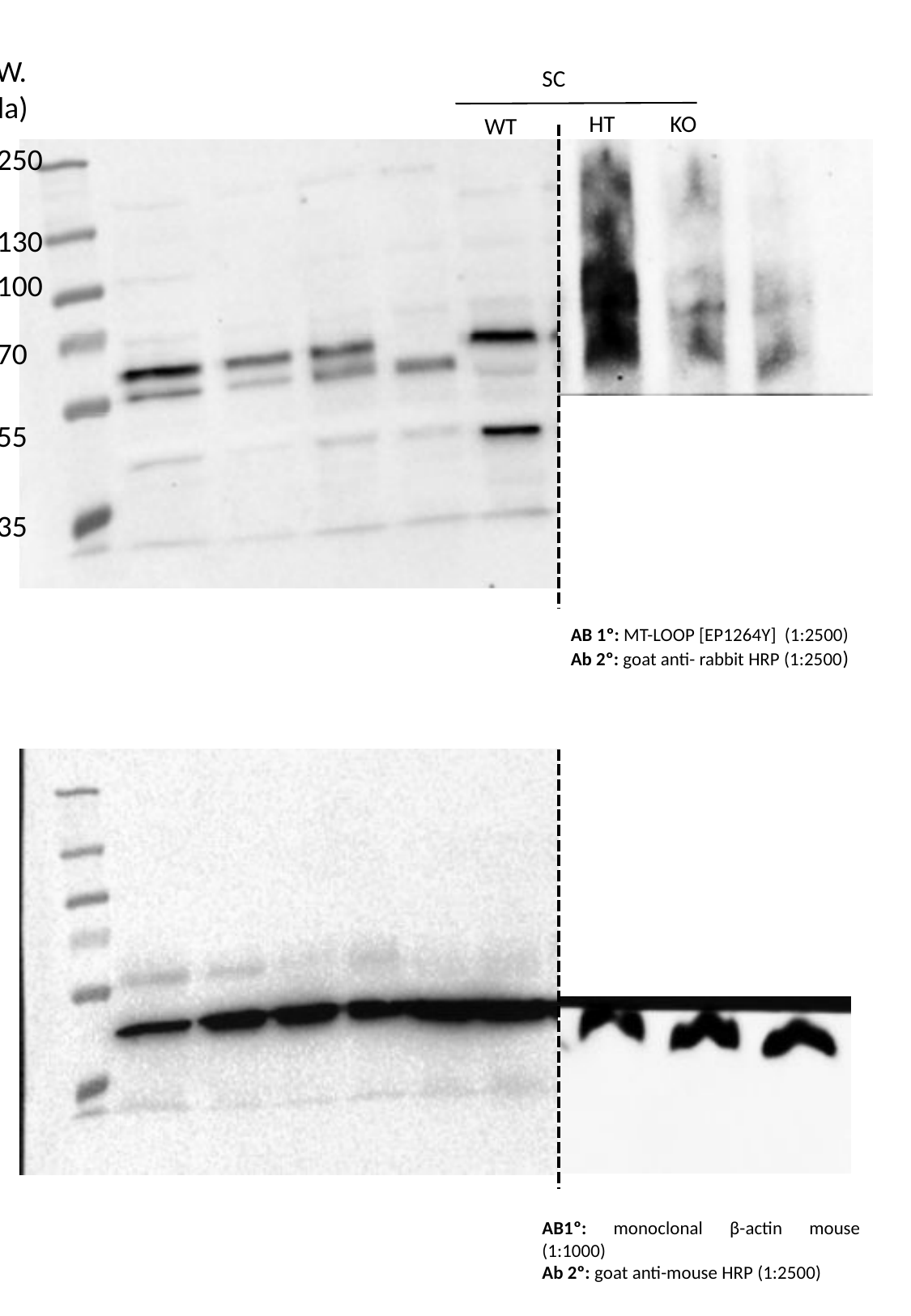

M.W. (Kda)
SC
KO
HT
WT
250
130
100
70
55
35
AB 1º: MT-LOOP [EP1264Y] (1:2500)
Ab 2º: goat anti- rabbit HRP (1:2500)
AB1º: monoclonal β-actin mouse (1:1000)
Ab 2º: goat anti-mouse HRP (1:2500)

## Slide 14
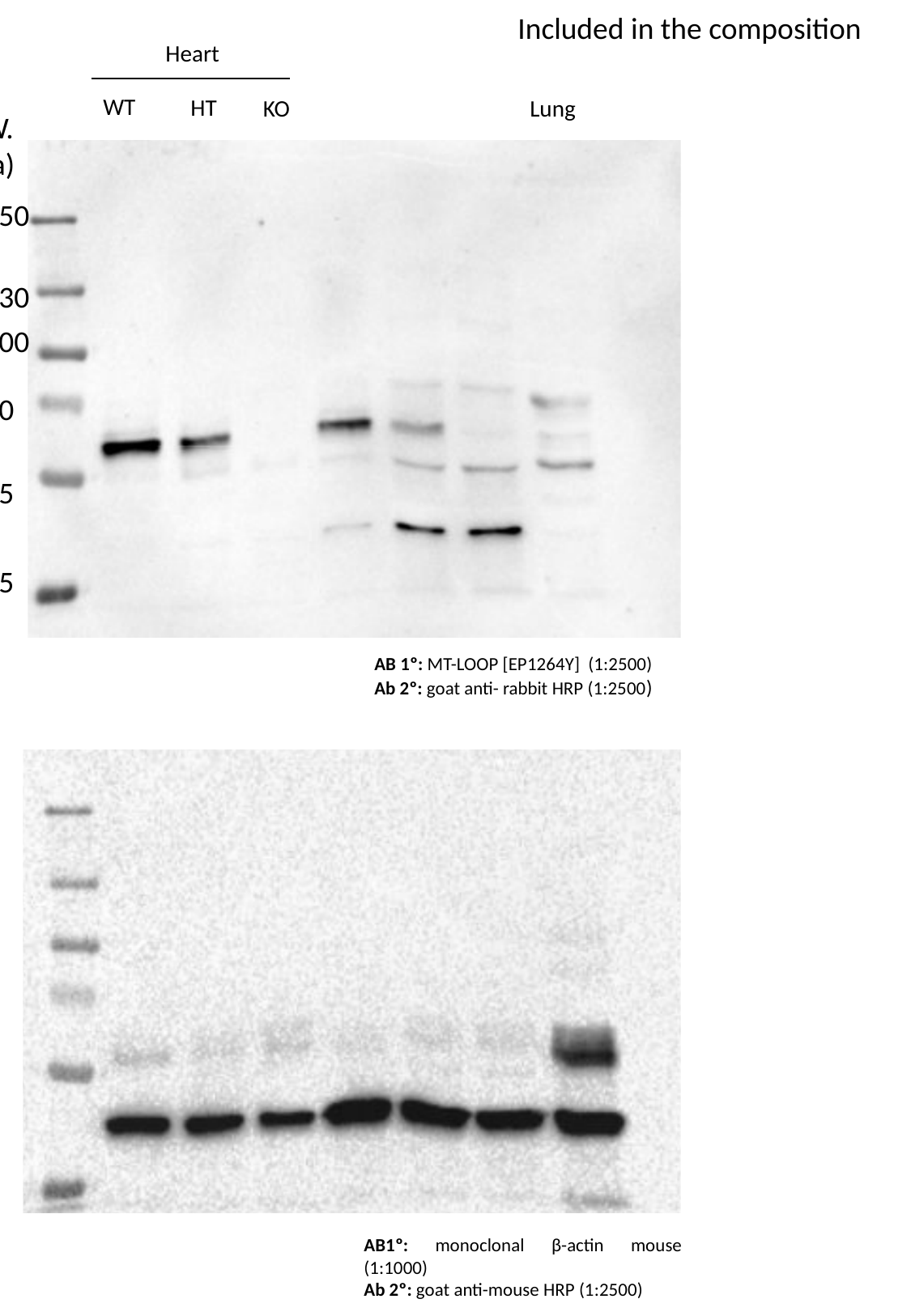

Included in the composition
Heart
WT
HT
Lung
KO
M.W. (Kda)
250
130
100
70
55
35
AB 1º: MT-LOOP [EP1264Y] (1:2500)
Ab 2º: goat anti- rabbit HRP (1:2500)
AB1º: monoclonal β-actin mouse (1:1000)
Ab 2º: goat anti-mouse HRP (1:2500)

## Slide 15
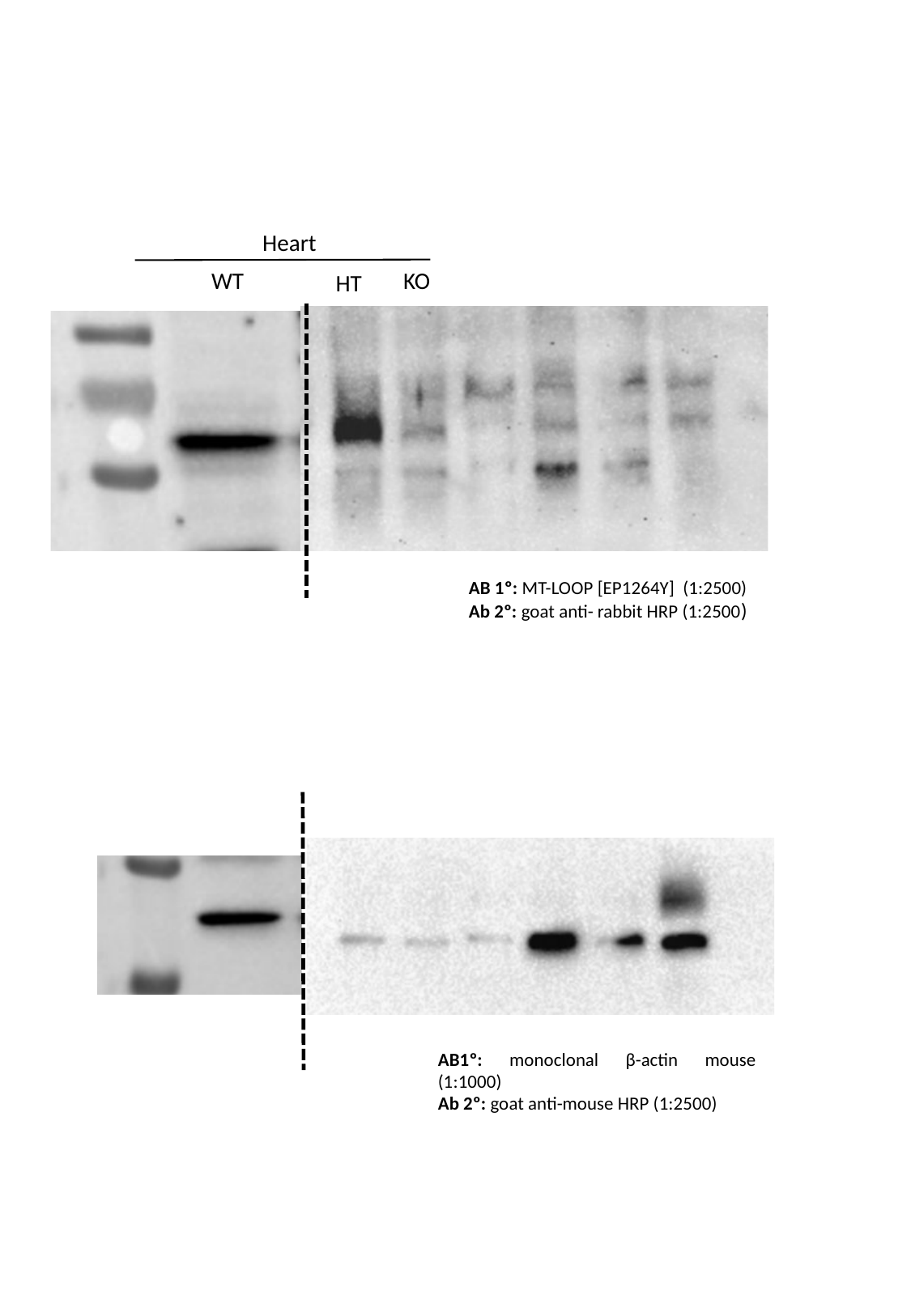

Heart
WT
KO
HT
AB 1º: MT-LOOP [EP1264Y] (1:2500)
Ab 2º: goat anti- rabbit HRP (1:2500)
AB1º: monoclonal β-actin mouse (1:1000)
Ab 2º: goat anti-mouse HRP (1:2500)

## Slide 16
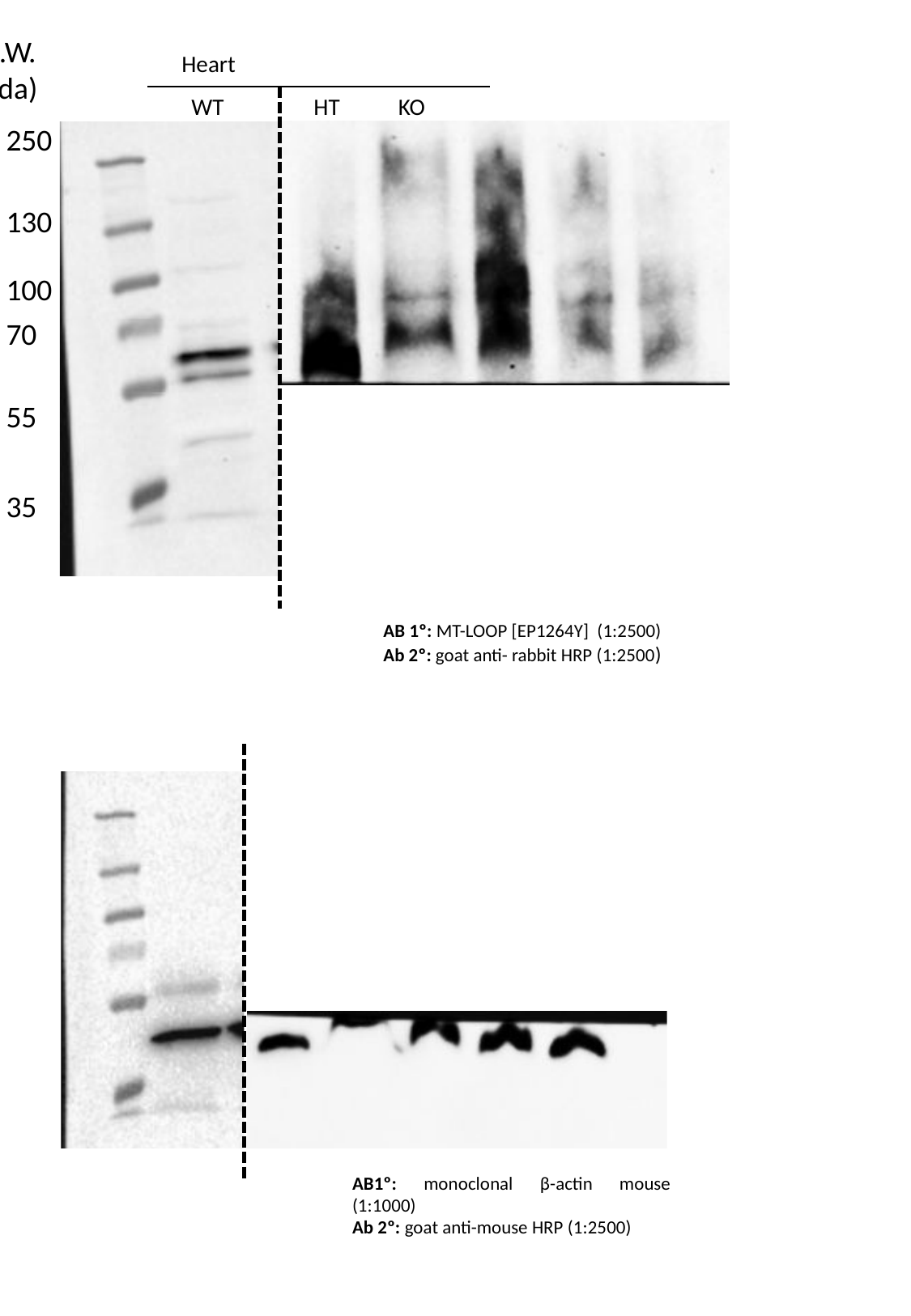

M.W. (Kda)
Heart
WT
HT
KO
250
130
100
70
55
35
AB 1º: MT-LOOP [EP1264Y] (1:2500)
Ab 2º: goat anti- rabbit HRP (1:2500)
AB1º: monoclonal β-actin mouse (1:1000)
Ab 2º: goat anti-mouse HRP (1:2500)

## Slide 17
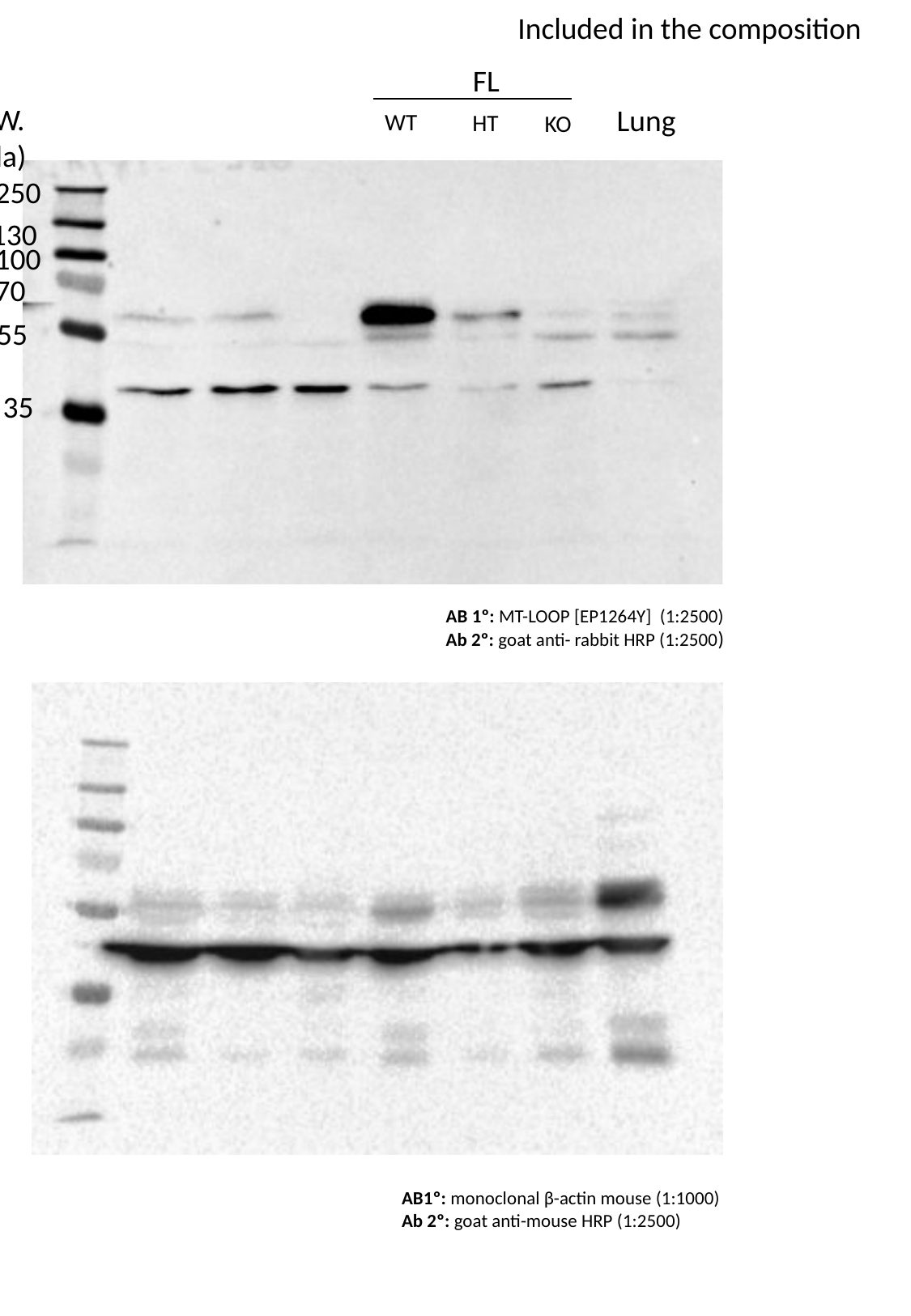

Included in the composition
FL
WT
HT
KO
M.W. (Kda)
Lung
250
130
100
70
55
35
AB 1º: MT-LOOP [EP1264Y] (1:2500)
Ab 2º: goat anti- rabbit HRP (1:2500)
AB1º: monoclonal β-actin mouse (1:1000)
Ab 2º: goat anti-mouse HRP (1:2500)

## Slide 18
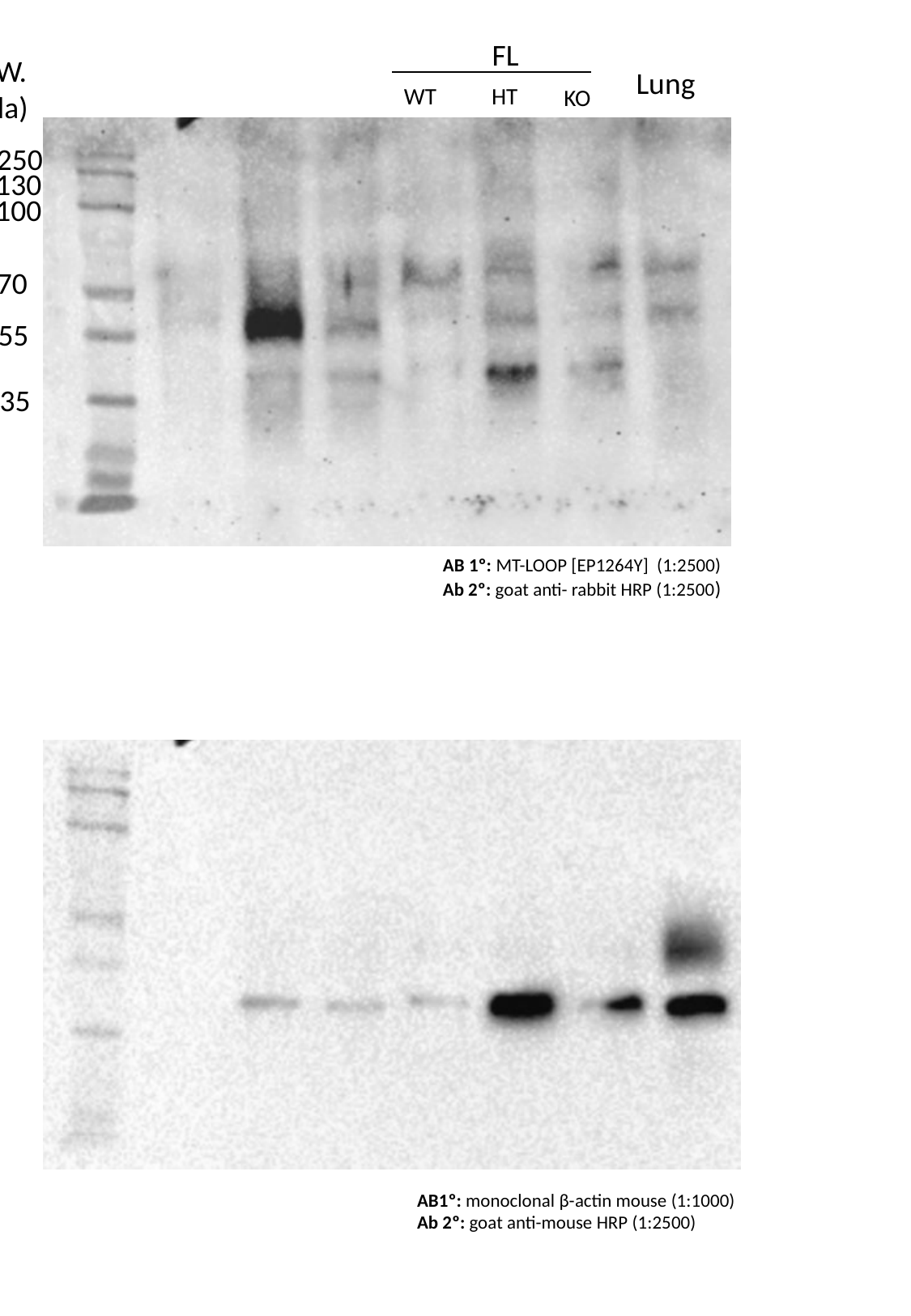

FL
WT
HT
KO
M.W. (Kda)
Lung
250
130
100
70
55
35
AB 1º: MT-LOOP [EP1264Y] (1:2500)
Ab 2º: goat anti- rabbit HRP (1:2500)
AB1º: monoclonal β-actin mouse (1:1000)
Ab 2º: goat anti-mouse HRP (1:2500)

## Slide 19
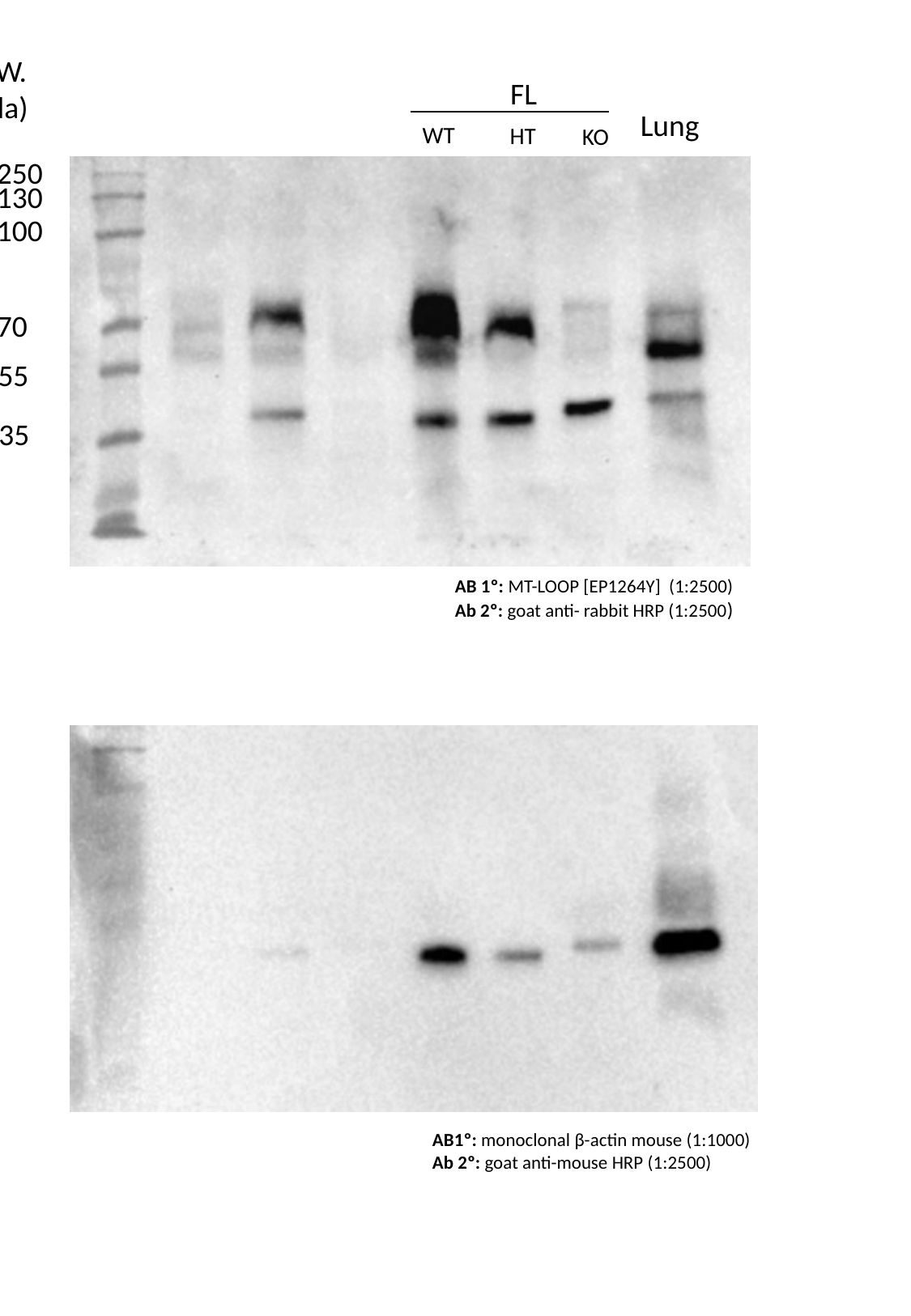

M.W. (Kda)
FL
WT
HT
KO
Lung
250
130
100
70
55
35
AB 1º: MT-LOOP [EP1264Y] (1:2500)
Ab 2º: goat anti- rabbit HRP (1:2500)
AB1º: monoclonal β-actin mouse (1:1000)
Ab 2º: goat anti-mouse HRP (1:2500)
