# Supplementary figures and images for: Dynamic Expression of Membrane Type 1-Matrix Metalloproteinase (Mt1-mmp/Mmp14) in the Mouse Embryo
Source: Cells. 2021 Sep 17;10(9):2448. doi: 10.3390/cells10092448 (PMC8465375; doi:10.3390/cells10092448)

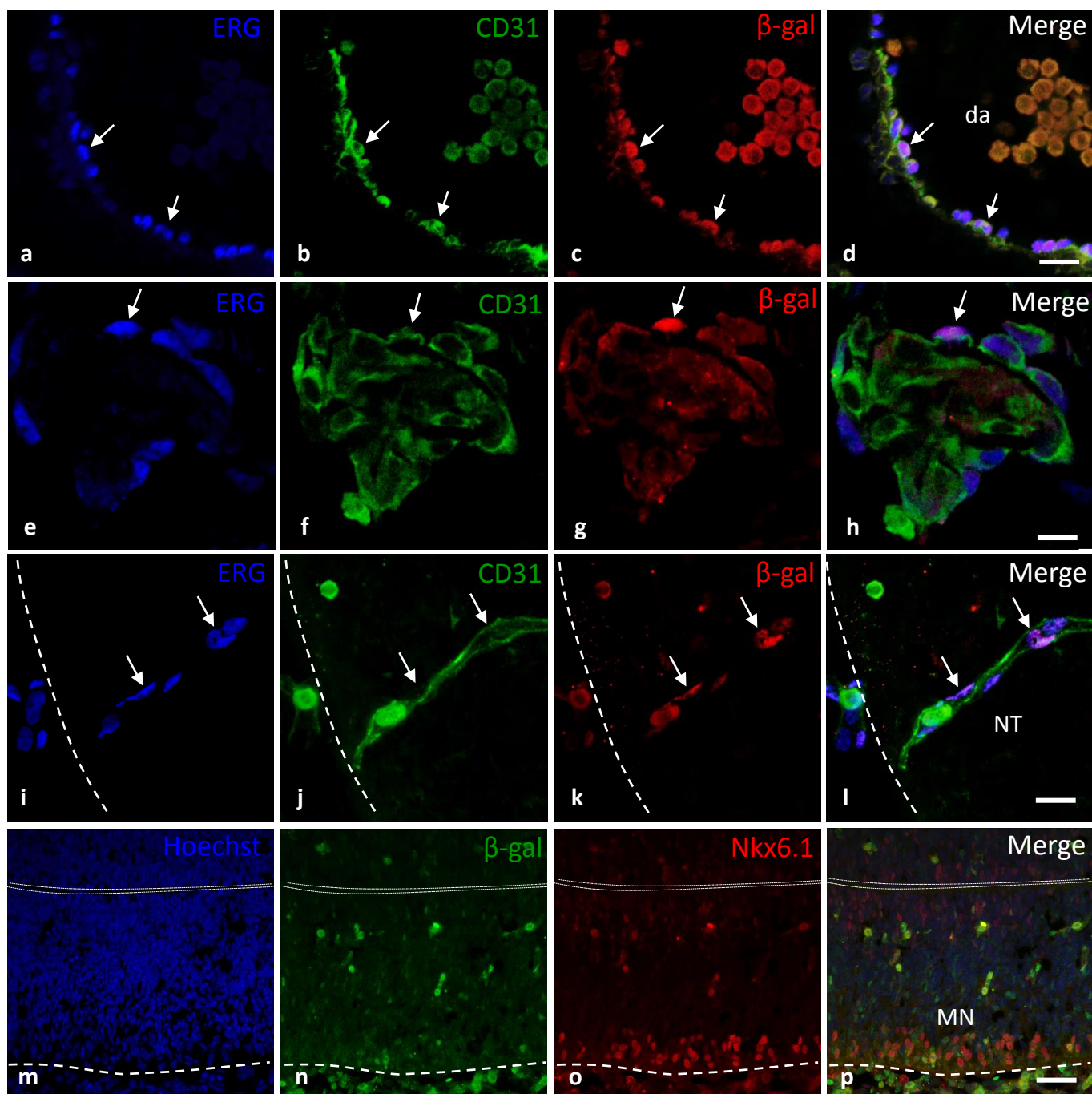

Supplement: Supplementary file 1 [file cells-10-02448-s001.zip › Sumplemmentary Material/Fig S1.pdf]

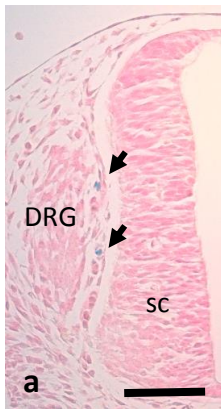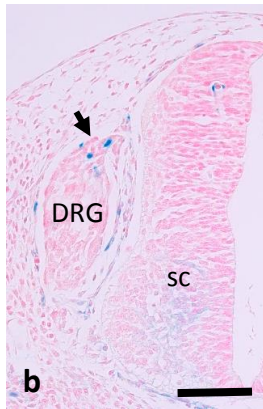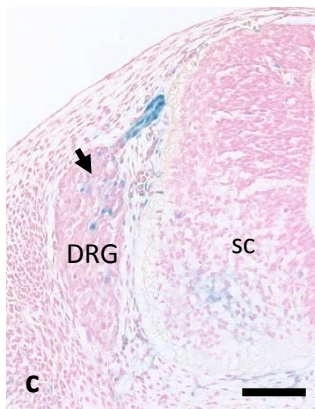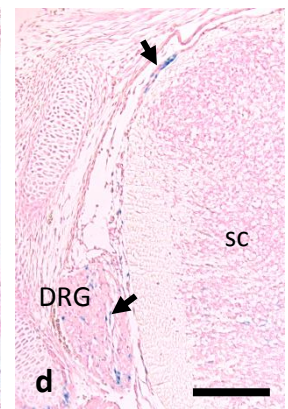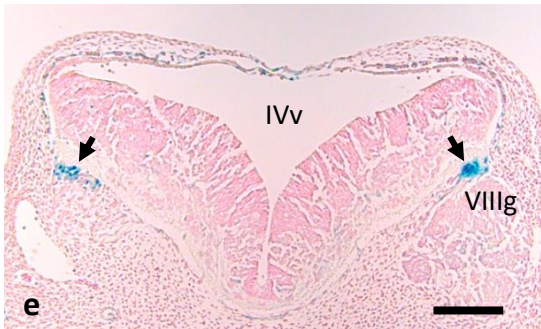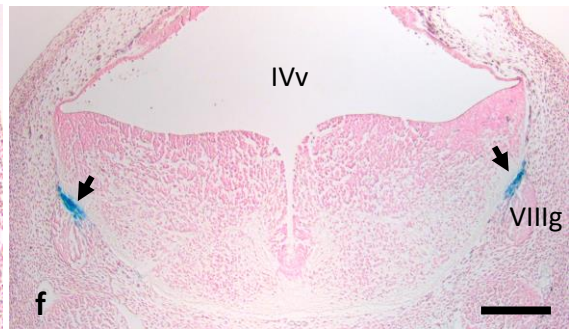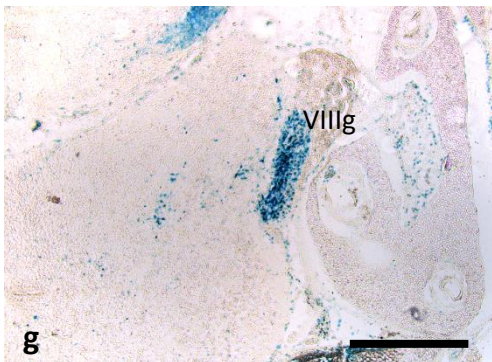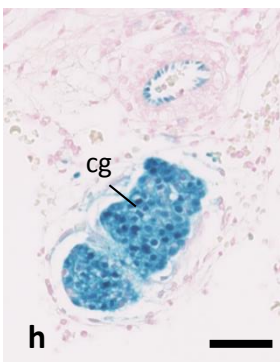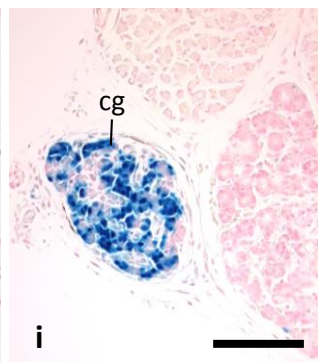

Supplement: Supplementary file 1 [file cells-10-02448-s001.zip › Sumplemmentary Material/Fig S2.pdf]
